# Palladium-catalyzed regioselective C1-selective nitration of carbazoles

**DOI:** 10.3762/bjoc.21.190

**Published:** 2025-11-10

**Authors:** Vikash Kumar, Jyothis Dharaniyedath, Aiswarya T P, Sk Ariyan, Chitrothu Venkatesh, Parthasarathy Gandeepan

**Affiliations:** 1 Department of Chemistry, Indian Institute of Technology Tirupati, Yerpedu- Venkatagiri Road, Yerpedu Post, Tirupati District, Andhra Pradesh 517619, India

**Keywords:** C–H activation, carbazole, catalysis, nitration, palladium

## Abstract

Carbazoles are ubiquitous and privileged heterocyclic scaffolds in various functional organic materials and naturally occurring products. Although extensive efforts have focused on developing synthetic strategies toward carbazole derivatives, direct regioselective functionalization of the carbazole core remains challenging due to the inherently higher reactivity at the C3/C6 positions. In this study, we report a palladium-catalyzed, directing group-assisted, regioselective C1–H nitration of carbazoles. The protocol features a removable directing group and is amenable to gram-scale synthesis. This strategy provides a valuable platform for the selective functionalization of carbazoles, offering potential applications in optoelectronics, functional organic materials, and related areas while contributing to the advancement of C–H activation methodologies.

## Introduction

Carbazole represents an important heterocyclic scaffold that is broadly present in many natural products, biologically active motifs, as well as optoelectronic and functional materials [1–8]. By virtue of its substantial application in various fields, significant attention has been devoted to the chemical synthesis of carbazole and its derivatives [9–14]. To access substituted carbazole cores for pharmacophores and functional materials, two main synthetic routes are: i) sequential multistep syntheses of selectively substituted carbazoles and ii) functionalization of the carbazole core. Traditional approaches for constructing diversified carbazoles and derivatives are Fischer–Borsche synthesis [15–16], Graebe–Ullmann synthesis [17–18], cyclization of biaryl nitrenes–Cadogan synthesis [19], electrocyclic reactions [20–21], and others [22–24]. These methodologies are greatly limited due to harsh reaction conditions that impact the scope of the reaction, poor yield, and regioselectivity issues. In sharp contrast, transition metal-catalyzed cross-coupling reactions promisingly improve the regioselectivity issues and substrate scope [25–28]. In addition to cross-coupling reactions, transition metal-catalyzed cyclization involving C–H activation approaches have also been reported [29–36]. Despite the significant advances in carbazole core constructions, the established protocols significantly lack access to selectively C1-decorated carbazoles. Consequently, functionalizing carbazoles via transition metal-catalyzed directed C–H activation becomes more attractive to introduce the desired functionality in a regioselective fashion. The C–H activation strategy is elegant in many ways since it utilizes nonfunctionalized or lesser functionalized starting materials, reduces organometallic waste generation, has a broad substrate scope and a higher functional group compatibility, and features better resource- and step-economies [37–47]. In this context, several recent studies enabled the regioselective functionalization of carbazoles via C–H activation [48–61].

**Scheme 1:**
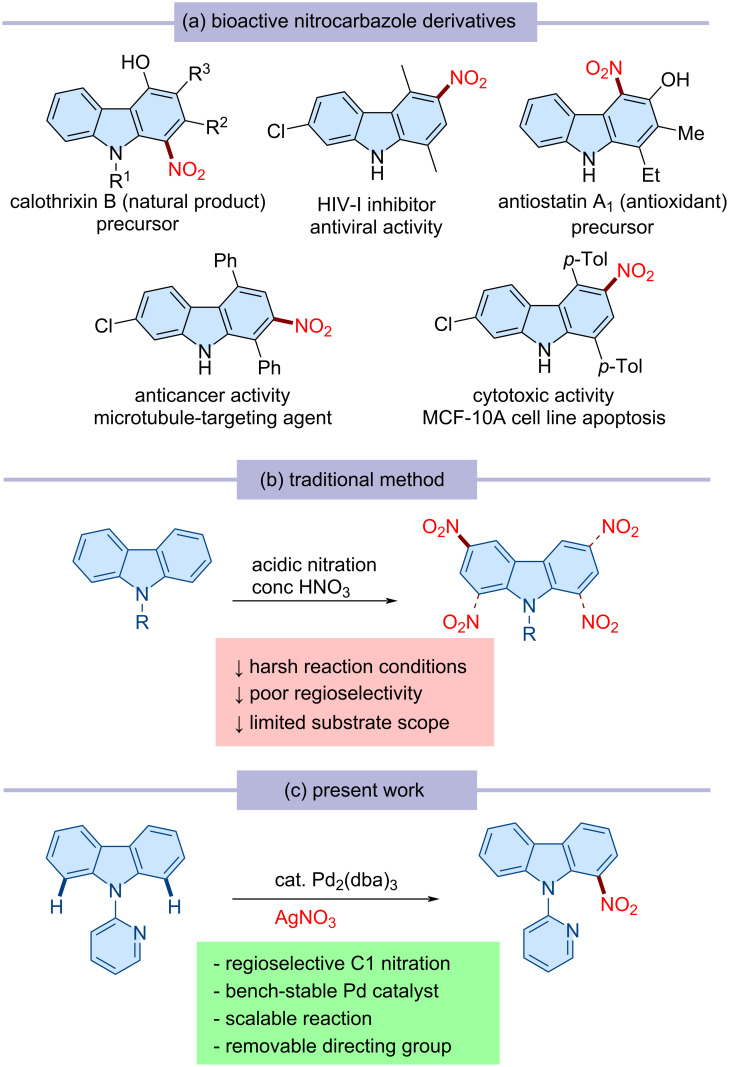
(a) Representative examples of bioactive nitrocarbazoles. (b) Traditional electrophilic aromatic substitution approach for the nitration of carbazole. (c) Present work: palladium-catalyzed directed C1-selective nitration reaction.

Among the variously substituted carbazole scaffolds, nitro-substituted carbazoles exhibit a diverse range of medicinal properties and serve as key starting materials for the synthesis of bioactive compounds and functional materials (Scheme 1a) [62–66]. Traditional electrophilic aromatic substitution methods for the nitration of carbazole typically result in a mixture of 1-nitro-, 2-nitro-, and 3-nitro-substituted isomers (Scheme 1b) [67]. Therefore, developing a method for the regioselective nitration of carbazole is highly desirable [68–69]. In this context, we envisioned utilizing a directing group-assisted regioselective C–H activation strategy to achieve the C1-selective nitration of carbazole. Through our efforts, we identified a palladium-catalyzed reaction system for C1 nitration of carbazole, which is presented in this study (Scheme 1c).

## Results and Discussion

### Optimization of the reaction parameters

**Figure 1:**
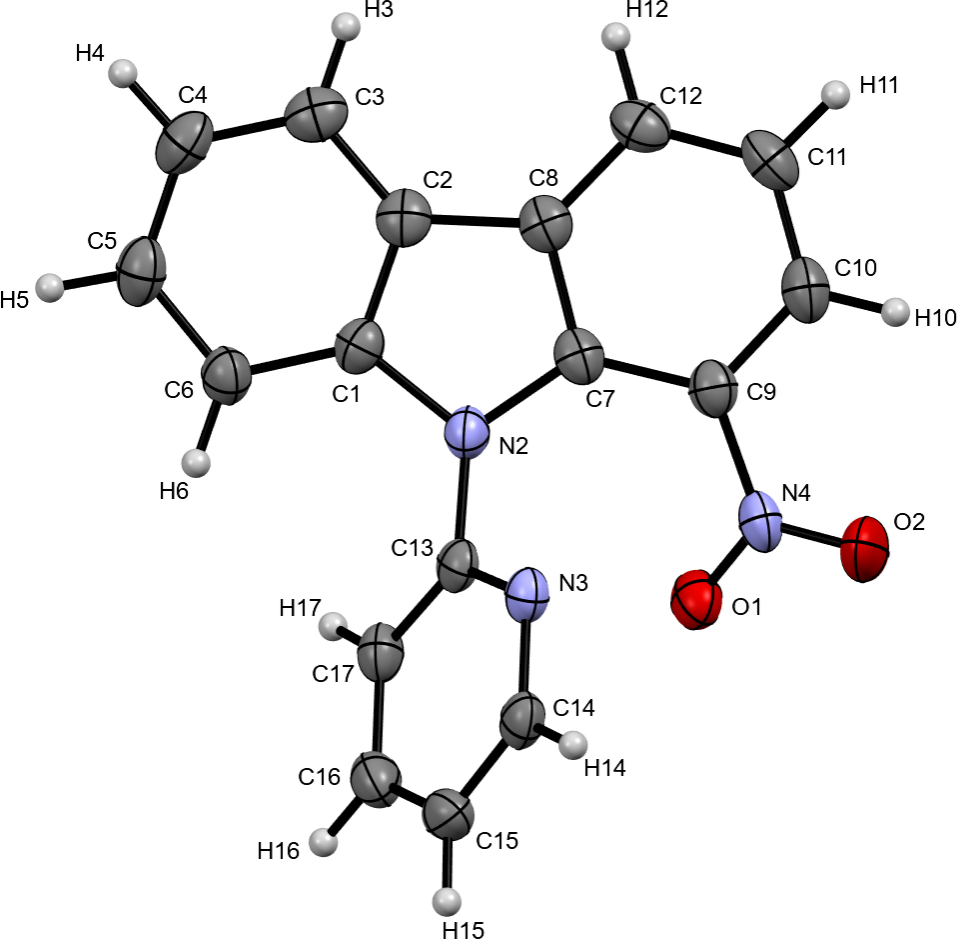
ORTEP diagram of compound **2a** (CCDC 2478298).

To evaluate the feasibility of the reaction, we commenced our studies by exploring palladium-catalyzed regioselective *ortho*-C–H nitration of the *N*-pyridylcarbazole **1a** as the model substrate, using silver nitrate as the nitrating agent (Table 1). After detailed optimization studies, we found that the treatment of **1a** with AgNO_3_ in the presence of Pd_2_(dba)_3_ as the catalyst in 1,4-dioxane afforded the desired C1-nitrated product **2a** in 69% isolated yield (Table 1, entry 1). Product **2a** was thoroughly characterized by ^1^H and ^13^C NMR spectroscopy, HRMS, and single-crystal X-ray diffraction analysis (Figure 1). A range of solvents was subsequently screened; however, none provided an improvement over 1,4-dioxane for this transformation (Table 1, entries 2 and 3). In addition to AgNO_3_, other silver and copper salts were examined as an oxidant to enhance the yield. Surprisingly, none of the tested silver salts afforded the desired product, while copper salts resulted in either trace amounts or a lower yield of **2a** (Table 1, entries 4 and 5). We also evaluated inorganic oxidants, but these oxidants were ineffective in improving the reaction outcome (Table 1, entry 6). Other palladium complexes promoted the formation of **2a** but with diminished efficiency (Table 1, entries 7 and 8). Varying the amount of AgNO_3_ did also not lead to a better yield (Table 1, entry 9), and alternative nitro sources also failed to enhance the product **2a** yield (Table 1, entry 10). Attempts to optimize the reaction temperature did not yield improvements either (Table 1, entry 11). Control experiments confirmed that both the palladium catalyst and AgNO_3_ are essential for the reaction to proceed, as omission of either reagent resulted in complete loss of reactivity (Table 1, entry 12). Further, extending the reaction time had no significant effect on the yield (Table 1, entry 13). Finally, our investigation using various 3d transition metal catalysts such as Ni(OAc)_2_, Cu(OAc)_2_, and Co(OAc)_2_ in place of Pd₂(dba)_3_ did not lead to the formation of the desired product (Table 1, entry 14).

**Table 1:** Optimization studies.^a^

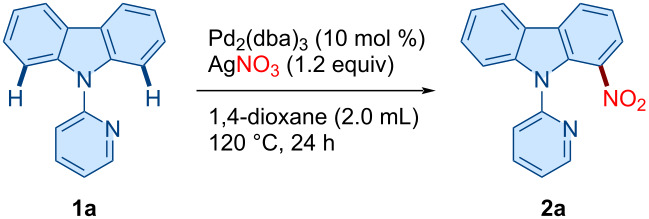		

entry	deviation from standard conditions	yield (%)^b,c^

1	no deviation	69
2	DCE/toluene/MeCN/GVL/cyrene	ND
3	THF/DMSO/MeOH/AcOH/DME	trace
4^d^	1.0 equiv AgOAc/Ag_2_O/Ag_2_CO_3_	trace
5^d^	1.0 equiv CuOAc/Cu(OAc)_2_/Cu(OAc)_2_⋅H_2_O	10/30/trace
6^d^	1.0 equiv K_2_S_2_O_8_/(NH_4_)_2_S_2_O_8_	57/33
7^e^	PdCl_2_/Pd(PPh_3_)_2_Cl_2_/Pd(PPh_3_)_4_/Pd(acac)_2_	25/24/(13)/(10)
8^e^	Pd(dba)_2_/Pd(OAc)_2_	(37)/34
9	1.5 equiv AgNO_3_	60
10^f^	1.2 equiv FeNO_3_⋅9H_2_O/AgNO_2_/HNO_3_/*t*-BuNO_2_/iBuNO_2_	43/31/48/46/46
11	100/140 °C reaction temperature	27/14
12	without Pd_2_(dba)_3_ catalyst or AgNO_3_	ND
13	reaction time 48 h	69
14	1.0 equiv Ni(OAc)_2_ or Cu(OAc)_2_ or Co(OAc)_2_ in place of Pd_2_(dba)_3_	ND

^a^Reaction conditions: 9-(pyridin-2-yl)-9*H*-carbazole (**1a,** 0.2 mmol, 1.0 equiv), Pd_2_(dba)_3_ (0.02 mmol, 10 mol %), AgNO_3_ (0.24 mmol, 1.2 equiv), and 1,4-dioxane (2.0 mL) at 120 °C for 24 h. ND = not detected. ^b^Yield of isolated product. ^c^Yield in parenthesis was determined by ^1^H NMR using mesitylene as internal standard. ^d^1.0 equiv of oxidant was used along with AgNO_3_. ^e^10 mol % of different palladium catalysts was used instead of Pd_2_(dba)_3_. ^f^1.2 equiv of the different nitro sources used instead of AgNO_3_.

**Scheme 2:**
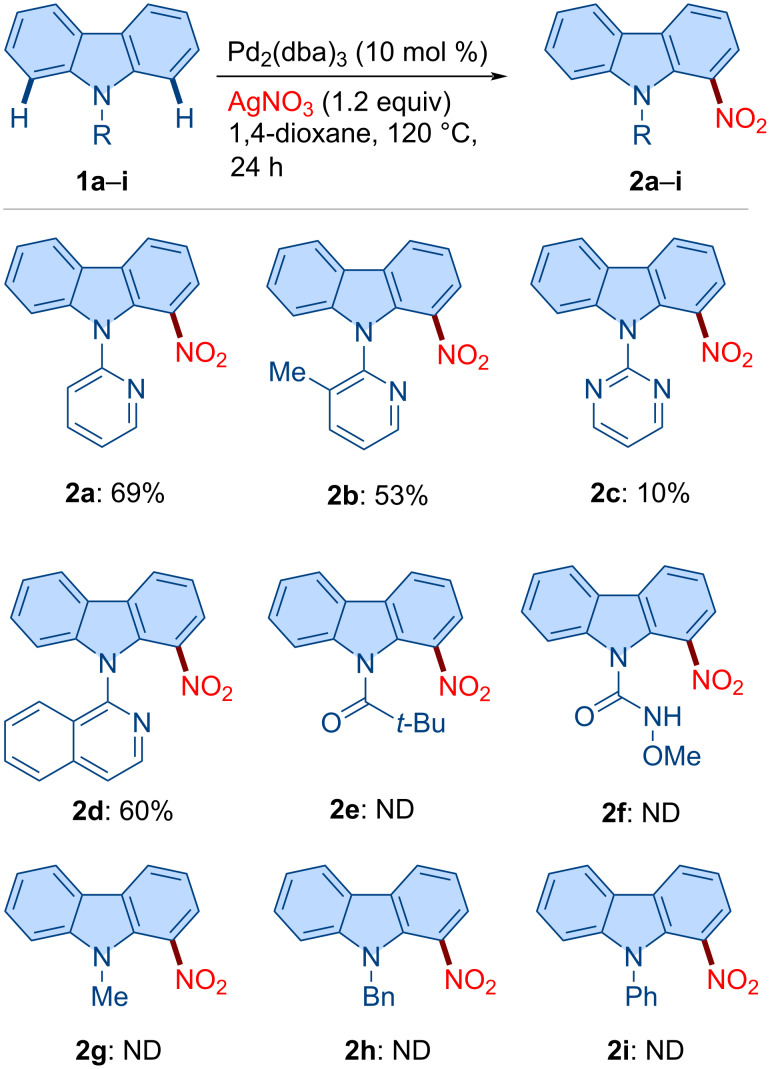
Effect of directing groups on the nitration of the carbazoles.

Building on the envisioned C1–H nitration of carbazoles, we further investigated the influence of various directing groups under the optimized reaction conditions (Scheme 2). Among the commonly employed directing groups tested, the 2-pyridyl group emerged as the most effective, enabling regioselective C1–H nitration to afford product **2a**. Notably, control experiments using *N*-aryl-/*N*-alkyl-protected carbazoles and *N*-unsubstituted carbazole under standard conditions failed to produce the desired product.

### Substrate scope

**Scheme 3:**
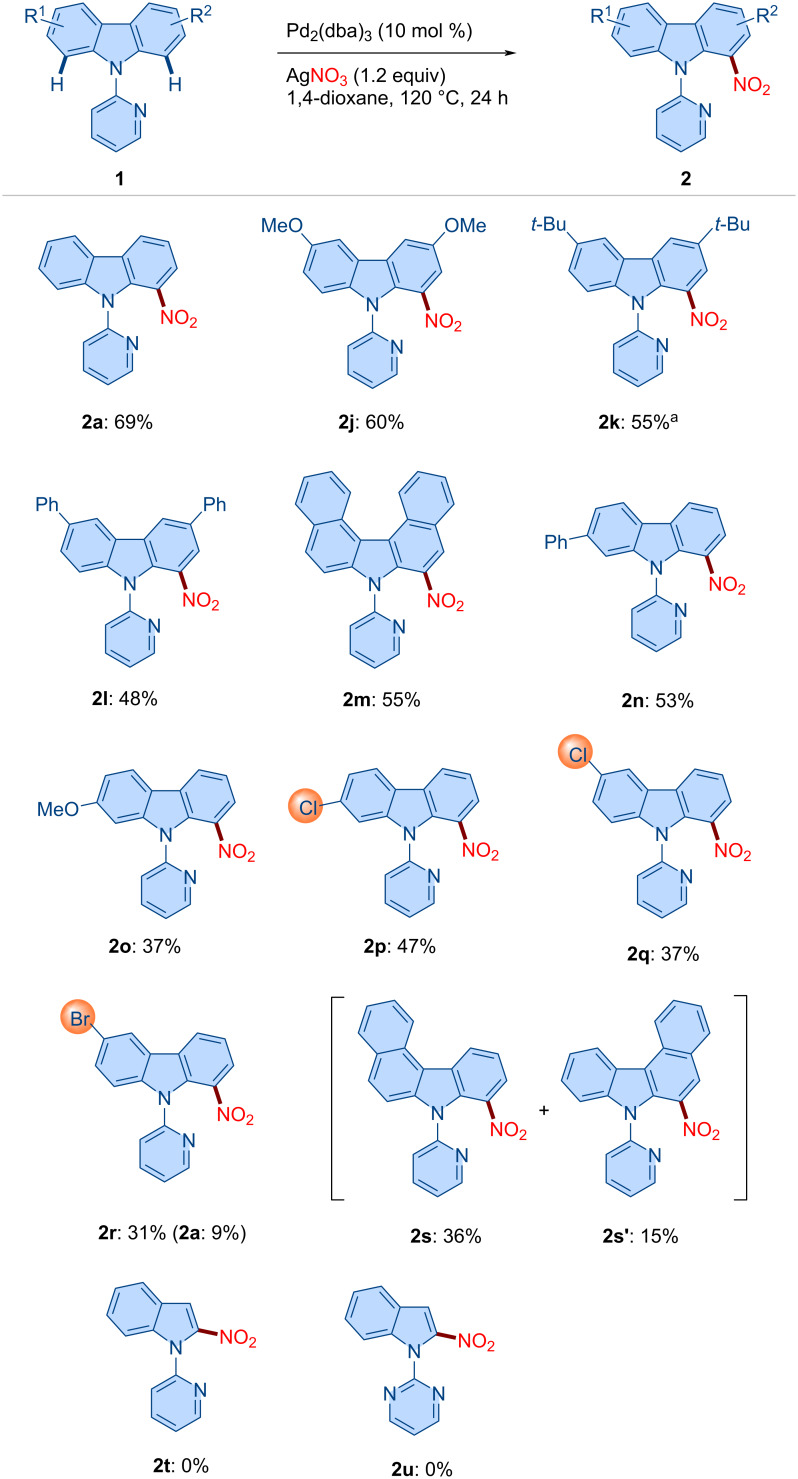
Scope of the method. Reaction conditions: **1** (0.2 mmol, 1.0 equiv), Pd_2_(dba)_3_ (0.02 mmol, 10 mol %), AgNO_3_ (0.24 mmol, 1.2 equiv), and 1,4-dioxane (2.0 mL) at 120 °C for 24 h. ^a^10 mol % of Pd(OAc)_2_ was used instead of Pd_2_(dba)_3_.

With the optimized reaction conditions in hand, we next explored the substrate scope (Scheme 3). The unsubstituted *N*-pyridylcarbazole **1a** delivered the desired C1-nitrated product **2a** in 69% yield. Carbazoles bearing 3,6-disubstitution (see **1j**–**l**) participated smoothly, furnishing products **2j**–**l** in good yield. The dibenzocarbazole derivative **1m** underwent nitration to afford **2m** in 55% yield. Likewise, 2-Ph-, 2-OMe-, and 2-Cl-substituted carbazoles were efficiently converted under the standard conditions, affording the corresponding C1 nitration products **2n**–**p** in good yield, whereby C–H activation selectively occurred at the less hindered site. Halogenated substrates **1q** and **1r** (3-Cl and 3-Br substitution) delivered products **2q** (37%) and **2r** (31%) in moderate yield. Notably, the reaction of **1r** also furnished **2a** (9%), indicating competitive debromination. Finally, benzocarbazole substrate **1s** afforded a mixture of regioisomers **2s** (36%) and **2s'** (15%). It is worth noting that under the standard reaction conditions, indole substrates **1t** and **1u** failed to afford the desired products **2t** and **2u**.

**Scheme 4:**
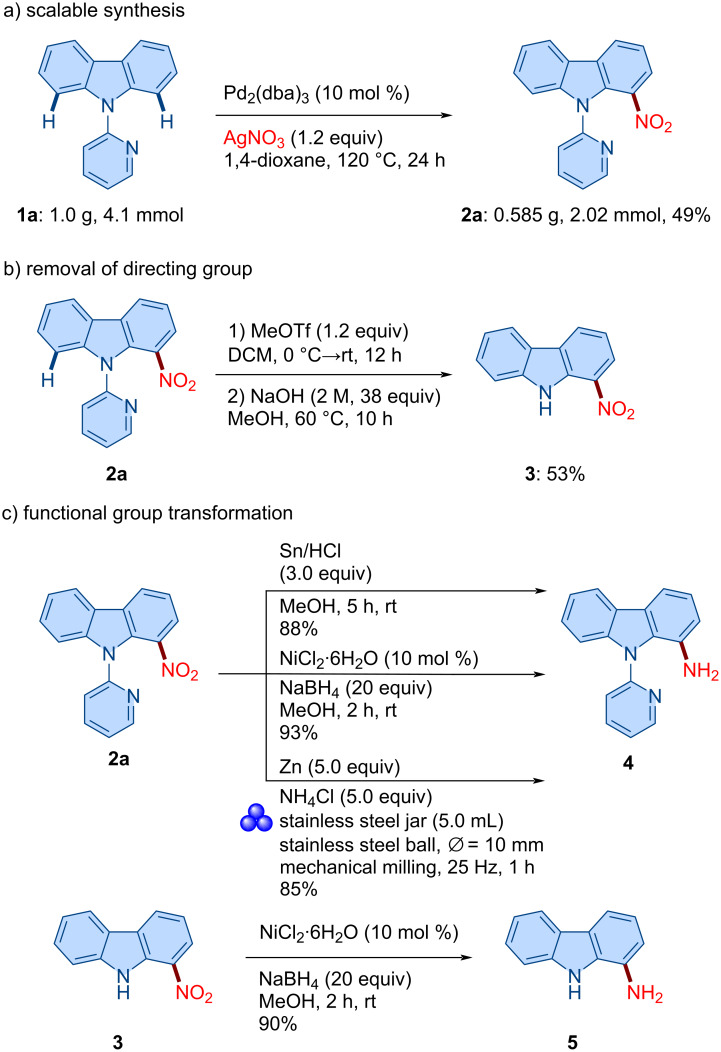
Gram-scale synthesis, directing group removal, and synthetic utility of our method.

Encouraged by these results, and to further demonstrate the synthetic utility of the established reaction protocol, we carried out a gram-scale synthesis under the optimized conditions. The reaction of **1a** was performed on a 4.1 mmol (1.0 g) scale, yielding the C1–H-nitrated carbazole product **2a** in 49% (0.585 g, 2.02 mmol) isolated yield (Scheme 4a). Given the importance of nitro-functionalized (hetero)arenes, we sought to access the NH-unsubstituted carbazole bearing a nitro group by removing the pyridyl directing group [58]. Treatment of compound **2a** with methyl triflate, followed by hydrolysis with sodium hydroxide, successfully delivered the deprotected carbazole **3** in 53% yield (Scheme 4b).

Next, we demonstrated the reduction of the nitro group in compound **2a** (Scheme 4c) [70–73]. Three distinct reaction conditions were found to be the most suitable to afford product **4** from **2a**. Thus, the treatment of **2a** with Sn/HCl gave 1-aminocarbazole derivative **4** in 88% yield. Furthermore, the reduction was also achieved using NiCl_2_/NaBH_4_ in methanol at room temperature, affording the corresponding amine **4** in 93% yield. To align with green chemistry principles, we employed a recently reported mechanochemical protocol by Ito and co-workers for the reduction of nitro compounds to amines [72]. Using this solvent-free method, compound **2a** was successfully reduced to the corresponding amine **4** in 85% yield. Similarly, the nitro group of compound **3** was also reduced under the NiCl_2_/NaBH_4_-promoted conditions, affording 9*H*-carbazol-1-amine (**5**) in 90% yield.

### Mechanistic studies

**Scheme 5:**
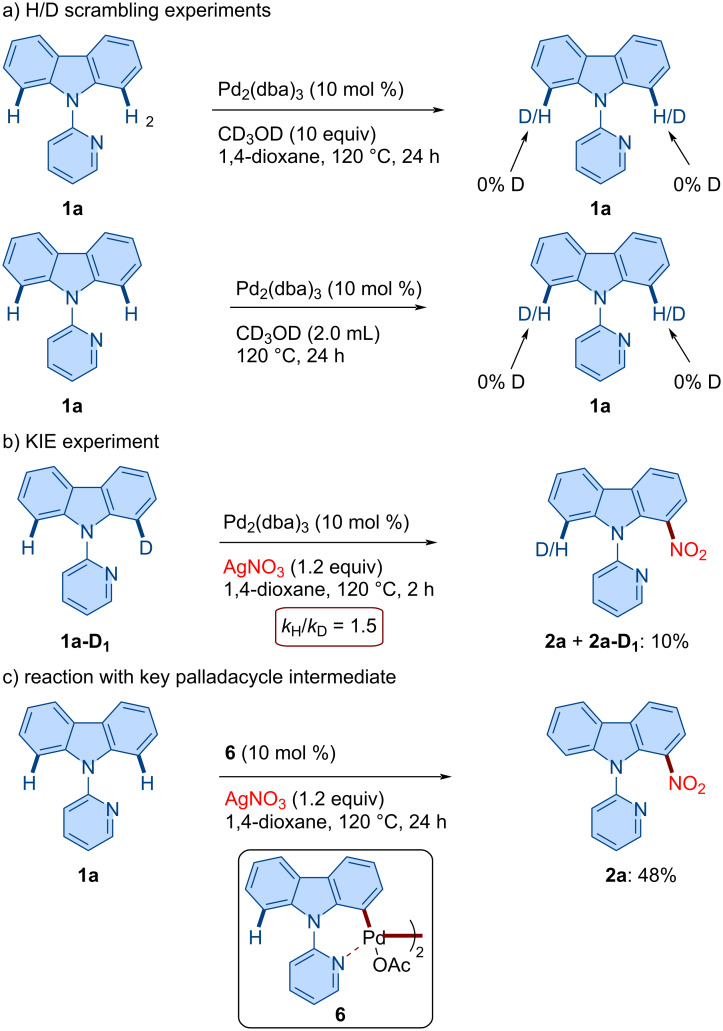
Key mechanistic studies.

Next, we sought to gain mechanistic insights into the catalytic pathway through a series of experiments (Scheme 5). To probe the nature of the C–H activation step, the reaction was conducted in fully deuterated methanol as both cosolvent and solvent (Scheme 5a). No H/D scrambling was observed at the C1 position of the recovered starting material **1a**, suggesting that the cyclopalladation step is irreversible. To further investigate the nature of the C–H activation step, an intramolecular kinetic isotope effect (KIE) experiment was performed using monodeuterated substrate **1a-D_1_**. A modest KIE value of *k*_H_/*k*_D_ = 1.5 was observed under standard reaction conditions after 2 hours, indicating that C–H bond cleavage is kinetically relevant and likely involved in the rate-determining step (Scheme 5b). To gain additional mechanistic insight, we synthesized palladacycle intermediate **6** following the reported procedure [58]. Then, the reaction was carried out using palladacycle **6** as the catalyst, and the desired nitrated product **2a** was obtained in 48% yield (Scheme 5c). This result supports the involvement of palladacycle intermediate **6** in the catalytic cycle.

### Proposed mechanism

**Figure 2:**
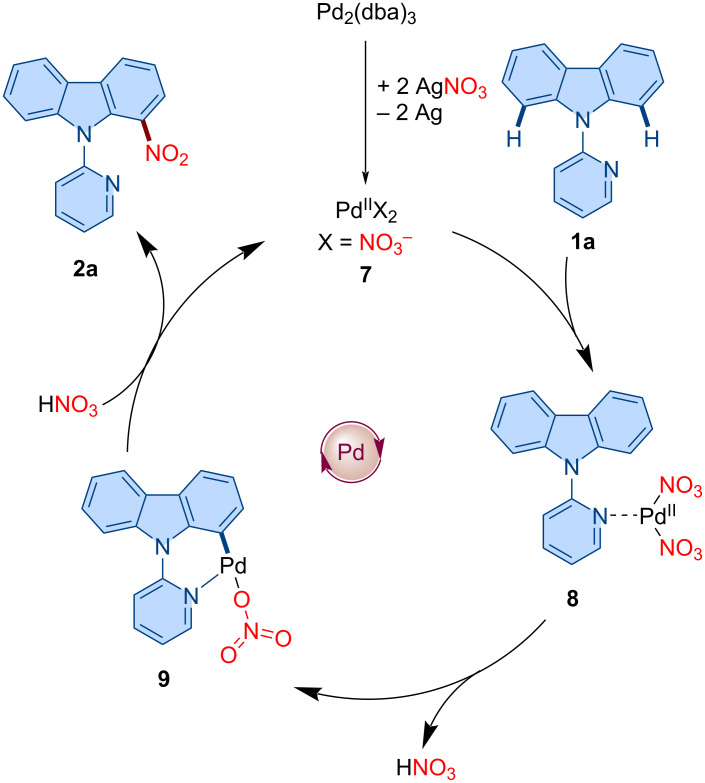
Plausible catalytic cycle.

Based on our experimental results and related literature precedents [68–69,74–80], a plausible catalytic cycle is proposed (Figure 2). The catalytic cycle commences with the formation of active palladium(II) species **7** in the presence of AgNO_3_. Coordination of the pyridyl group of **1a** to Pd(NO₃)₂ is followed by irreversible C–H bond cleavage via cyclopalladation to form a six-membered palladacycle intermediate **9**. Subsequent reaction with in situ-generated HNO_3_ facilitates nitro group incorporation to form the C1-nitrated carbazole product **2a** and regeneration of the active palladium catalyst **7**, thereby completing the catalytic cycle.

## Conclusion

In summary, we have developed a regioselective protocol for the direct C1–H nitration of carbazoles, an important class of heterocycles with wide-ranging applications in materials science and natural products chemistry. The transformation proceeds using commercially available Pd_2_(dba)_3_ as the catalyst and silver nitrate as the nitro source. The catalytic system demonstrates a satisfactory substrate scope and excellent regioselectivity. The scalability of the reaction was demonstrated, further underscoring the robustness of the protocol. Overall, this study highlights the potential of palladium-catalyzed C–H activation strategies in streamlining access to nitro-functionalized carbazoles for applications in organic synthesis and materials science.

## Experimental

A 15 mL pressure tube was charged with Pd_2_(dba)_3_ (18.3 mg, 0.02 mmol, 10 mol %), *N*-(pyridin-2-yl)-9*H*-carbazole **1** (0.2 mmol, 1.0 equiv), and AgNO_3_ (41 mg, 0.24 mmol, 1.2 equiv). Then, the solvent 1,4-dioxane (2.0 mL) was added, and the reaction mixture was allowed to stir in a preheated oil bath at 120 °C for 24 h. Upon completion of the reaction time, the reaction mixture was cooled to room temperature and diluted with dichloromethane (10 mL). The reaction mixture was filtered through a Celite pad, and the filtrate was concentrated using a rotary evaporator. The crude residue was purified through silica gel column chromatography using *n*-hexane/EtOAc 99:1 as eluent to give the pure C1-nitrated carbazole **2**.

## Supporting Information

File 1 Experiment details, characterization data, copy of NMR spectra of synthesized compounds, and single-crystal X-ray diffraction data.

File 2 CIF file for **2a**.

## Data Availability

All data that supports the findings of this study is available in the published article and/or the supporting information of this article.
