# Supplementary material for: Palladium-catalyzed regioselective C1-selective nitration of carbazoles
Source: Beilstein J Org Chem. 2025 Nov 10;21:2479–88. doi: 10.3762/bjoc.21.190 (PMC12621620; doi:10.3762/bjoc.21.190)

BEILSTEIN JOURNAL OF ORGANIC CHEMISTRY

Supporting Information

for

Palladium-catalyzed regioselective C1-selective nitration of carbazoles

Vikash Kumar, Jyothis Dharaniyedath, Aiswarya T P, Sk Ariyan, Chitrothu Venkatesh and Parthasarathy Gandeepan

Beilstein J. Org. Chem. **2025,** *21,* 2479–2488. doi:10.3762/bjoc.21.190

Experiment details, characterization data, copy of NMR spectra of synthesized compounds, and single-crystal X-ray diffraction data

License and Terms: This is a supporting information file under the terms of the Creative Commons Attribution License (https://creativecommons.org/licenses/by/4.0). Please note that the reuse, redistribution and reproduction in particular requires that the author(s) and source are credited and that individual graphics may be subject to special legal provisions.
The license is subject to the *Beilstein Journal of Organic Chemistry* terms and conditions: (https://www.beilstein-journals.org/bjoc/terms)

<u>Table of contents</u>

**Contents**	**Page no.**
1. General information	S2
2. Starting materials	S3
3. Optimization studies	S4
4. Experimental section	S11
5. Synthetic applications of nitrated carbazoles	S12
6. Mechanistic studies	S15
7. Cyclic voltammetry studies	S18
8. Spectral data	S19
9. Crystal X-ray diffraction data of **2a**	S35
10. References	S41
11. NMR spectra	S42

1. General information

Unless otherwise mentioned, all catalysts, starting materials and solvents were purchased from commercial sources (Sigma, TCI, Avra, SRL, Spectrochem, BLD Pharm) and used as received. All the reactions were carried out in a screw cap reaction tube (15 mL) with magnetic stirring under air atmosphere in flame-dried glassware in preheated oil bath at 80 °C. In case air- or moisture sensitive reagents were used, reactions were performed under N_2 atmosphere using standard Schlenk techniques. Yields refer to isolated compounds, estimated to be >95% pure, as determined by H NMR. Thin layer chromatogram (TLC) was performed on Merck TLC Silica gel 60 F254, TLC plates; detection under UV light at 254 nm. The column chromatographic purifications were performed using Silica gel (100−200 mesh ASTM) from Merck, if not mentioned otherwise. Melting points were determined in capillary tubes using a Stuart melting point apparatus SMP10, the reported values are not corrected. Nuclear Magnetic Resonance (NMR) spectra ^{1}H NMR (400 MHz), ^{13}C NMR (101 MHz) were measured with a Bruker AVANCE NEO 400 MHz spectrometer using TMS as an internal standard and $CDCl_3$ as the solvent. Chemical shifts (δ) for ^{1}H and ^{13}C NMR spectra are given in ppm relative to tetramethylsilane (TMS) or the NMR solvents [δ 7.26 for ^{1}H (chloroform-*d*), δ 77.0 for ^{13}C (chloroform-*d*). Mass Spectrometer in electrospray ionization mode (ESI+) and an Agilent 6546 accurate–mass Q-TOF LC/MS (Agilent Technologies, U.S.A.) mass spectrometer in electrospray ionization (ESI+) mode. All IR spectra were recorded on a PerkinElmer Spectrum Two™ FT-IR-ATR device.

The cyclic voltammetry (CV) studies are carried out by using Metrohm Multi Autolab mo24 potentiostat instrument.

2. Starting materials

The substrate **1a–d, 1j–s**[1], **1e, 1h**[2], **1f**[3], **1g and 1i**[4] are prepared by using the reported procedure.

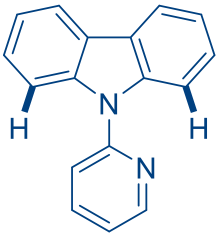

1a

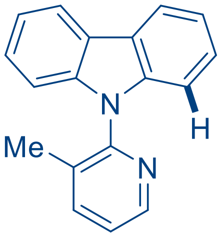

1b

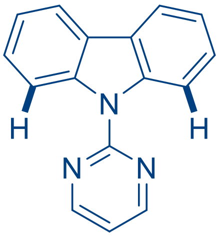

1c

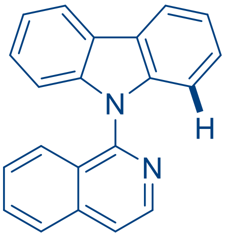

1d

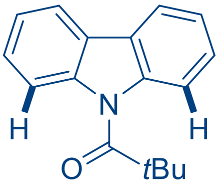

1e

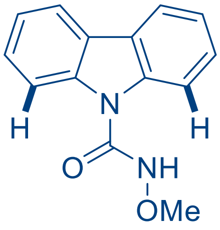

1f

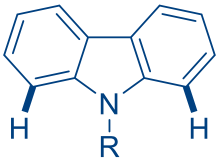

R = Me; **1g**
R = Bn; **1h**
R = Ph; **1i**

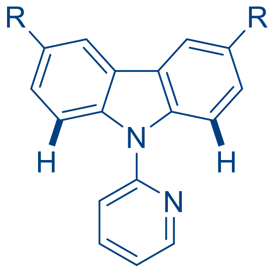

R = OMe; **1j**
R = *t*Bu; **1k**
R = Ph; **1l**

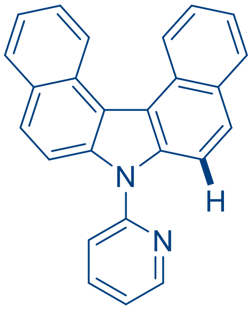

1m

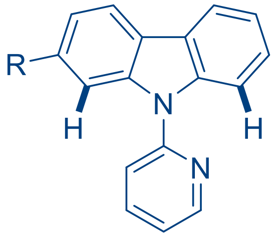

R = Ph; **1n**
R = OMe; **1o**
R = Cl; **1p**

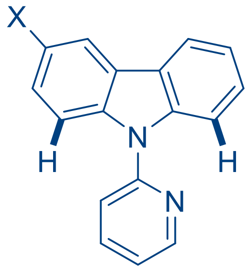

X = Cl; **1q**
X = Br; **1r**

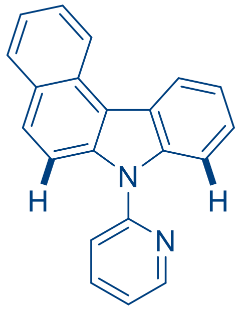

1s

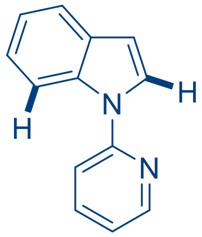

1t

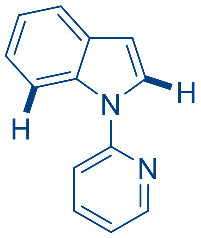

1u

3. Optimization studies

Table S1. Optimization of the solvent.[a]

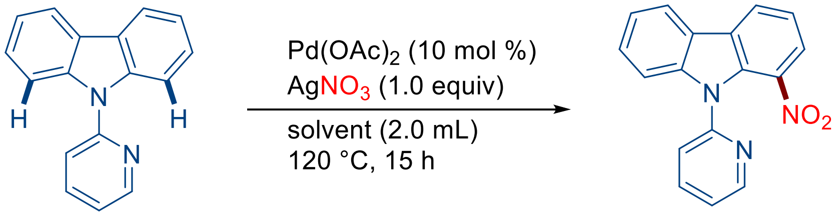

1a **2a**

entry	solvent (2.0 mL)	yield % (2a)[b]
1	DCE	no reaction
**2**	**1,4-dioxane**	**34**
3	DMF	trace conversion
4	toluene	ND
5	THF	trace conversion
6	DMSO	trace conversion
7	methanol	trace conversion
8	ACN	ND
9	acetic acid	trace conversion
10	GVL	ND
11	2-Me THF	trace conversion
12	DME	trace conversion
13	PEG 300	trace conversion
14	Cyrene	ND

[a] Reaction conditions: 9-(Pyridin-2-yl)-9*H*-carbazole (**1a**, 49 mg, 0.2 mmol, 1.0 equiv), $AgNO_3$ (34 mg, 0.2 mmol, 1.0 equiv), $Pd(OAc)_2$ (4.5 mg, 0.02 mmol, 10 mol %), and solvent (2.0 mL) at 120 °C for 15 h. [b] Isolated yields. ND = not detected.

Table S2. Optimization of the catalyst.[a]

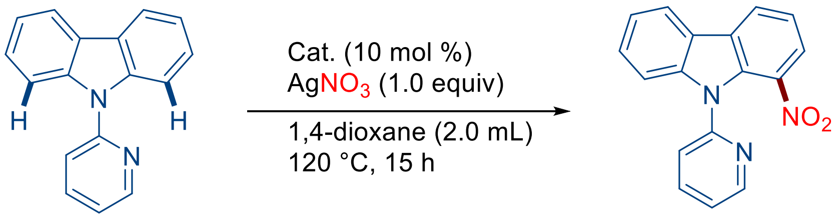

1a **2a**

entry	cat. (10 mol %)	yield % (2a)[b]
1	$Pd(OAc)_2$	34
2	$PdCl_2$	25[c]
3	$Pd(PPh_3)_2Cl_2$	24[c]
4	$Pd(PPh_3)_4$	13[c]
5	$Pd(acac)_2$	9[c]
6	$Pd(dba)_2$	37[c]
**7**	**$Pd_2(dba)_3$**	**58**
**8**	$Pd(OAc)_2$	49[d]
8	-	ND

[a] Reaction conditions: 9-(Pyridin-2-yl)-9*H*-carbazole (**1a**, 49 mg, 0.2 mmol, 1.0 equiv), $AgNO_3$ (34 mg, 0.2 mmol, 1.0 equiv), cat. (0.02 mmol, 10 mol %), and 1,4-dioxane (2.0 mL) at 120 °C for 15 h. [b] Isolated yields. [c] Yields were determined by ^{1}H NMR analysis of the crude reaction mixture using mesitylene as internal standard. [d] 20 mol % of $Pd(OAc)_2$ was used. ND = not detected.

Table S3. Optimization of the additive.[a]

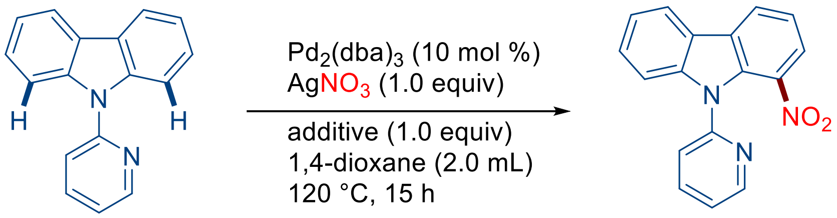

1a **2a**

entry	additive (1.0 equiv)	yield % (2a)[b]
1	AgOAc	trace
2	Ag_2O	trace
3	Ag_2CO_3	trace
4	$K_2S_2O_8$	57
5	$(NH_4)_2S_2O_8$	33
6	Na_2CO_3	ND
7	K_2CO_3	ND
8	$NaHCO_3$	ND
9	NaOAc	ND
10	KOAc	ND
11	NaO*t*-Bu	ND
12	LiO*t*-Bu	ND
13	Cu(OAc)	10
14	$Cu(OAc)_2$	30
15	$Cu(OAc)_2{\cdot}H_2O$	trace
16	benzoquinone	21
17	benzoyl peroxide	34[c]
18	di-*t*-butyl peroxide	33[c]
19	*t*-butyl hydrogen peroxide	38[c]

[a] Reaction conditions: 9-(Pyridin-2-yl)-9*H*-carbazole (**1a**, 49 mg, 0.2 mmol, 1.0 equiv), $AgNO_3$ (34 mg, 0.2 mmol, 1.0 equiv), $Pd_2(dba)_3$ (18.3 mg, 0.02 mmol, 10 mol %), additive (0.2 mmol, 1.0 equiv), and 1,4-dioxane (2.0 mL) at 120 °C for 15 h. [b] Isolated yields. [c] Yields were determined by ^{1}H MR analysis of the crude reaction mixture using mesitylene as internal standard. ND = not detected.

Table S4. Optimization of the reaction temperature.[a]

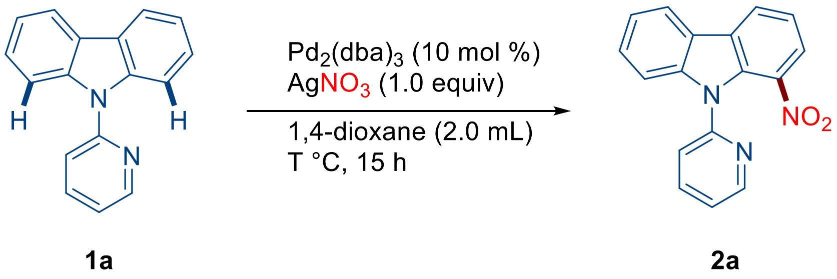

entry	*T* (°C)	Yield % (2a)[b]
1	100	27
**2**	**120**	**58**
3	140	14

[a] Reaction conditions: 9-(Pyridin-2-yl)-9*H*-carbazole (**1a**, 49 mg, 0.2 mmol, 1.0 equiv), $AgNO_3$ (34 mg, 0.2 mmol, 1.0 equiv), $Pd_2(dba)_3$ (18.3 mg, 0.02 mmol, 10 mol %), and 1,4-dioxane (2.0 mL) at *T* °C for 15 h. [b] Isolated yields. ND = not detected.

Table S5. Optimization of the ligand.[a]

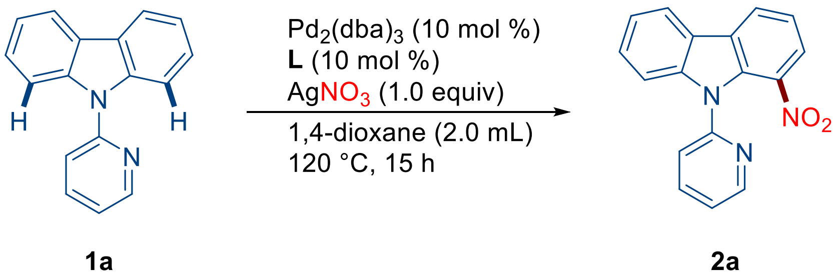

entry	L (10 mol %)	yield % (2a)[b]
1	DPPF	9
2	Xantphos	23
3	*rac*-BINAP	22
4	X-Phos	9
5	PPh_3	6
6	Brett Phos	7

[a] Reaction conditions: 9-(Pyridin-2-yl)-9*H*-carbazole (**1a**, 49 mg, 0.2 mmol, 1.0 equiv), $AgNO_3$ (34 mg, 0.2 mmol, 1.0 equiv), $Pd_2(dba)_3$ (18.3 mg, 0.02 mmol, 10 mol %), **L** (0.02 mmol, 10 mol %) and 1,4-dioxane (2.0 mL) at 120 °C for 15 h. [b] Isolated yields. ND = not detected.

Table S6. Optimization of the $AgNO_3$ amount.[a]

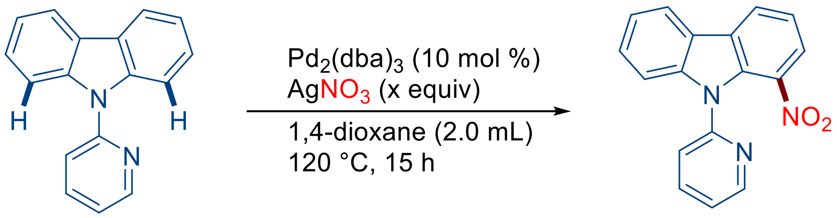

1a **2a**

entry	$AgNO_3$ (equiv)	yield % (2a)[b]
1	1.0	58
**2**	**1.2**	**69**
3	1.5	60
4	2.0	57
5	3.0	54

[a] Reaction conditions: 9-(Pyridin-2-yl)-9*H*-carbazole (**1a**, 49 mg, 0.2 mmol, 1.0 equiv), $AgNO_3$ (x equiv), $Pd_2(dba)_3$ (18.3 mg, 0.02 mmol, 10 mol %), and 1,4-dioxane (2.0 mL) at 120 °C for 15 h. [b] Isolated yields. ND = not detected.

Table S7. Optimization of the NO_2 source.[a]

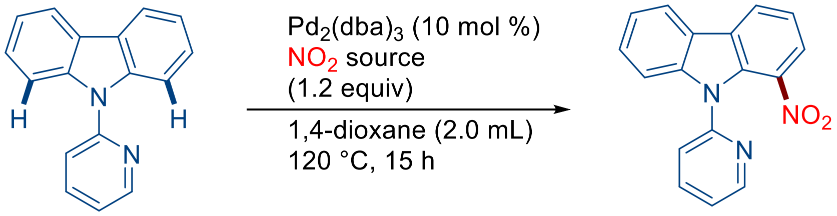

1a **2a**

entry	NO_2 source (1.2 equiv)	yield % (2a)[b]
**1**	**$AgNO_3$**	**69**
2	$FeNO_3{\cdot}9H_2O$	43
3	*t*-$BuNO_2$	46
4	$iBuNO_2$	46
5	HNO_3	48
6	$AgNO_2$	31
7	$NaNO_2$	ND
8	$NaNO_3$	ND
9	$Pb(NO_3)_2$	ND
10	-	ND

[a] Reaction conditions: 9-(Pyridin-2-yl)-9*H*-carbazole (**1a**, 49 mg, 0.2 mmol, 1.0 equiv), NO_2 source (0.24 mmol, 1.2 equiv), $Pd_2(dba)_3$ (18.3 mg, 0.02 mmol, 10 mol %), and 1,4-dioxane (2.0 mL) at 120 °C for 15 h. [b] Isolated yields. ND = not detected.

Table S8. Optimization of the reaction time.[a]

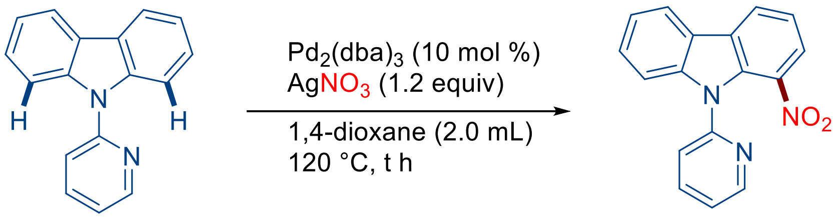

1a **2a**

entry	*t* (hours)	yield % (2a)[b]
1	15	58
**2**	**24**	**69**
3	48	69

[a] Reaction conditions: 9-(pyridin-2-yl)-9*H*-carbazole (**1a**, 49 mg, 0.2 mmol, 1.0 equiv), $AgNO_3$ (41 mg, 0.24 mmol, 1.2 equiv), $Pd_2(dba)_3$ (18.3 mg, 0.02 mmol, 10 mol %), and 1,4-dioxane (2.0 mL) at 120 °C for t h. [b] Isolated yields. ND = not detected.

4. Experimental section

4.1 General procedure (GP) for C1 nitration of carbazoles

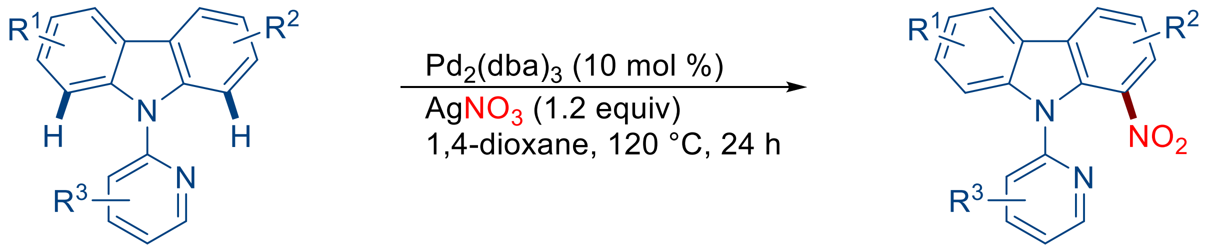

1 **2**

A 15 mL pressure tube was charged with $Pd_2(dba)_3$ (18.3 mg, 0.02 mmol, 10 mol %), *N*-(pyridin-2-yl)-9*H*-carbazole **1** (0.2 mmol, 1.0 equiv), and $AgNO_3$ (41 mg, 0.24 mmol, 1.2 equiv). Then, 1,4-dioxane solvent (2.0 mL) was added, and the reaction mixture allowed to stir in preheated oil bath at 120 ºC for 24 h. On completion of the reaction time, 10 mL of DCM was added for dilution of the reaction mixture. The crude mixture was filtered through a Celite pad, and the filtrate was concentrated using rotary evaporator. The crude residue was purified through silica gel column chromatography using *n*-hexane/EtOAc (99:1) as eluent to give the pure C1-nitrated carbazoles **2**.

4.2 Scalable synthesis

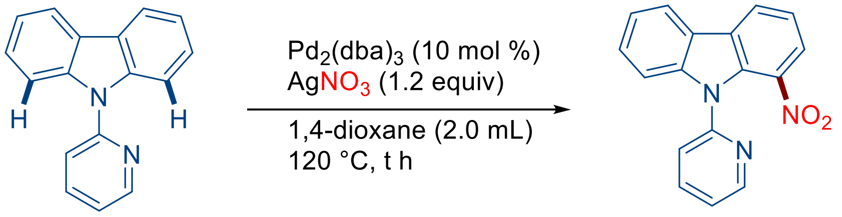

1a **2a**

Following the **GP**, a 100 mL pressure tube was charged with $Pd_2(dba)_3$ (375 mg, 0.409 mmol, 10 mol %), *N*-(pyridin-2-yl)-9*H*-carbazole (**1a**, 4.09 mmol, 1.0 equiv), and $AgNO_3$ (835 mg, 4.91 mmol, 1.2 equiv). Then, 1,4-dioxane solvent (40 mL) was added, and the reaction mixture allowed to stir in preheated oil bath at 120 ºC for 24 h. On completion of the reaction time, 50 mL of DCM was added for dilution of the reaction mixture. The crude was filtered through a Celite pad, and the filtrate was concentrated using rotary evaporator. The crude residue was purified through silica gel column chromatography using *n*-hexane/EtOAc (99:1) as eluent to give the pure product **2a** (0.585 g, 2.02 mmol) in 49% yield.

5. Synthetic applications of nitrated carbazoles

5.1 Removal of directing group[1]

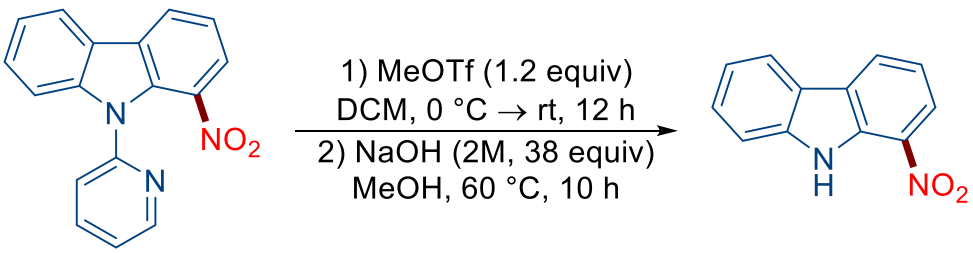

2a **3**: 53%

A 10 mL round bottom flask charged with 1-nitro-9-(pyridin-2-yl)-9*H*-carbazole (**2a**, 29 mg, 0.1 mmol, 1.0 equiv), and DCM (1.0 mL) was allowed to stir at 0 °C and slow addition of methyl trifluoromethanesulfonate (14.0 µL, 21.0 mg, 0.13 mmol, 1.2 equiv) was done using micro syringe. The reaction allowed to vigorously stir at room temperature for 12 h. The solvent was evaporated using reduced pressure, and the residue was further dissolved in methanol (2.0 mL). 2.0 M aqueous NaOH solution (1.2 mL) was added into it and the reaction continued at preheated oil bath at 60 ºC for 10 h. After reaction completion, the crude reaction mixture was concentrated under reduced pressure. The residue was quenched with H_2O (10 mL) and extracted with ethyl aetate (10 mL × 3). The combined organic layers were washed with brine, dried over Na_2SO_4, and concentrated using rotary evaporator. The desired product 1-nitro-9*H*-carbazole (**3**) was obtained in 53% (11 mg) yield by silica gel chromatography by using *n*-hexane/EtOAc (99:1) as eluent.

5.2 Synthesis of 9-(Pyridin-2-yl)-9*H*-carbazol-1-amine (4)

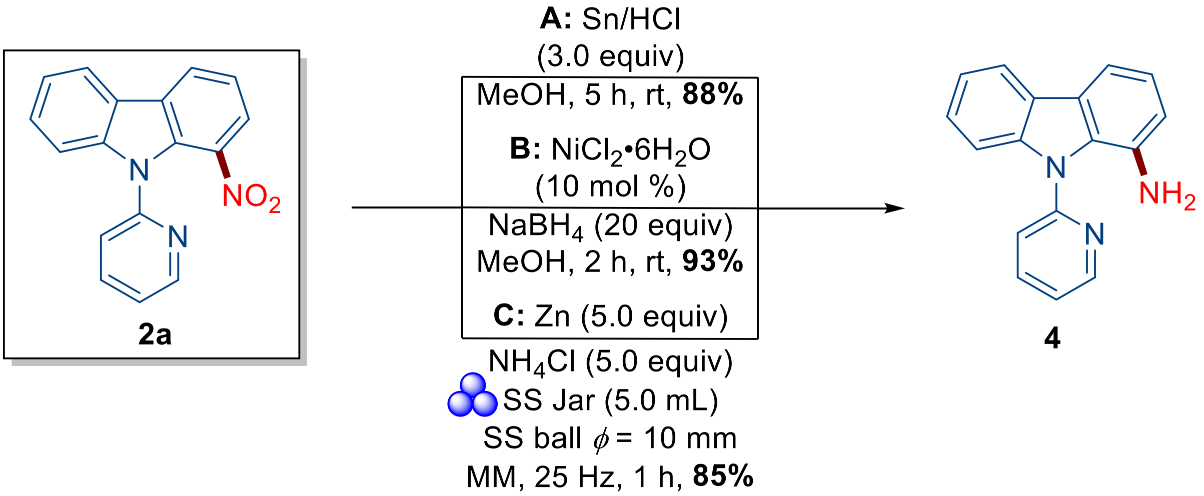

Condition A: A 15 mL schlenk tube charged with 1-nitro-9-(pyridin-2-yl)-9*H*-carbazole (**2a**, 29 mg, 0.1 mmol, 1.0 equiv), Sn powder (36 mg, 0.3 mmol, 3.0 equiv), and HCl (11 mg, 9 µL, 0.3 mmol, 3.0 equiv), and MeOH (1.5 mL). The reaction maintained to stir at room temperature (25 °C) for 5 h. Upon completion of reaction time, reaction mixture monitored by TLC, the crude mixture was concentrated using rotary evaporator and purified using silica gel column chromatography using *n*-hexane/EtOAc (2:1) to afford the desired product 9-(pyridin-2-yl)-9*H*-carbazol-1-amine (**4**) in 88% (23 mg) yield.[5]

Condition B: A 15 mL schlenk tube charged with 1-nitro-9-(pyridin-2-yl)-9*H*-carbazole (**2a**, 29 mg, 0.1 mmol, 1.0 equiv), $NiCl_2$•$6H_2O$ (2.4 mg, 0.01 mmol, 10 mol %), and $NaBH_4$ (76 mg, 2.0 mmol, 20 equiv), and MeOH (1.5 mL). The reaction maintained to stir at room temperature (25 °C) for 2 h. Upon completion of reaction time, reaction mixture monitored by TLC, the crude mixture was concentrated using rotary evaporator and purified using silica gel column chromatography using *n*-hexane/EtOAc (2:1) to afford the desired product 9-(pyridin-2-yl)-9*H*-carbazol-1-amine (**4**) in 93% (24 mg) yield.[6]

Condition C: A stainless-steel milling vessel (5.0 mL internal volume) was charged with 1-nitro-9-(pyridin-2-yl)-9*H*-carbazole (**2a**, 29 mg, 0.1 mmol, 1.0 equiv), Zn powder (33 mg, 0.5 mmol, 5.0 equiv), and NH_4Cl (27 mg, 0.5 mmol, 5.0 equiv), and a stainless-steel ball (10 mm diameter). The vessel was mounted into the holding station of a mixer mill and milling was conducted at 25 Hz frequency for 1 h. The vessel was then unmounted from the mixer mill and the crude reaction mixture was washed out with DCM. The crude mixture was concentrated using rotary evaporator and purified using silica gel column chromatography using *n*-

hexane/EtOAc (2:1) to afford the desired product 9-(pyridin-2-yl)-9*H*-carbazol-1-amine (**4**) in 85% (22 mg) yield.[7]

5.3 Synthesis of 9*H*-carbazol-1-amine (5)[6]

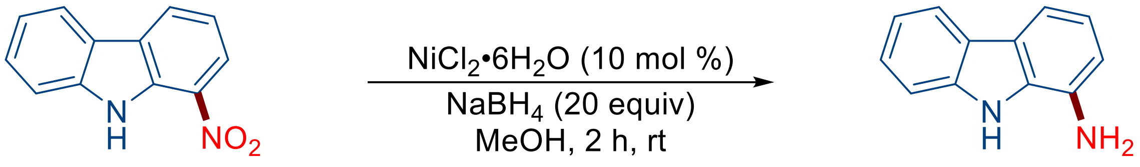

3 **5, 90%**

A 15 mL schlenk tube charged with 1-nitro-9*H*-carbazole (**3**, 21 mg, 0.1 mmol, 1.0 equiv), $NiCl_2•6H_2O$ (2.4 mg, 0.01 mmol, 10 mol %), and $NaBH_4$ (76 mg, 2.0 mmol, 20 equiv), and MeOH (1.5 mL). The reaction maintained to stir at room temperature (25 °C) for 2 h. Upon completion of reaction time, reaction mixture monitored by TLC, the crude mixture was concentrated using rotary evaporator and purified using silica gel column chromatography using *n*-hexane/EtOAc (2:1) to afford the desired product 9-(pyridin-2-yl)-9*H*-carbazol-1-amine (**5**) in 90% (16 mg) yield.

6. Mechanistic studies

6.1 H/D exchange studies

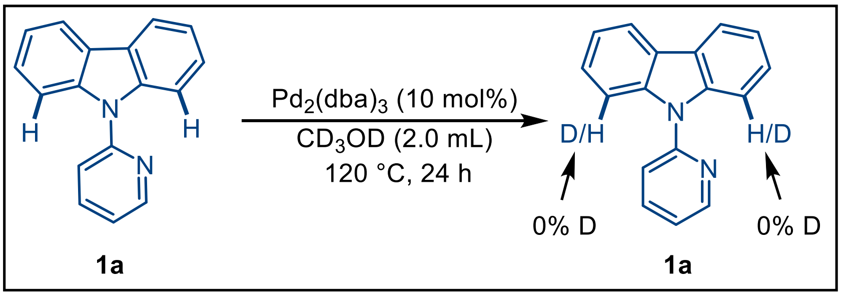

A 15 mL pressure tube was charged with $Pd_2(dba)_3$ (18.3 mg, 0.02 mmol, 10 mol %), corresponding *N*-(pyridin-2-yl)-9*H*-carbazole (**1a**, 49 mg, 0.2 mmol, 1.0 equiv). Then, CD_3OD (2.0 mL) was added, and the reaction mixture allowed to stir in preheated oil bath at 120 ºC for 24 h. On completion of reaction time, 5.0 mL of DCM was added for dilution of reaction mixture. The crude was filtered through a Celite pad, and the filtrate was concentrated using rotary evaporator. The crude residue was purified through a silica gel column chromatography using *n*-hexane/EtOAc (1:1) as eluent to give **1a** in 95% yield. The ^{1}H NMR analysis showed no deuterium incorporation observed at the C1/C8 position.

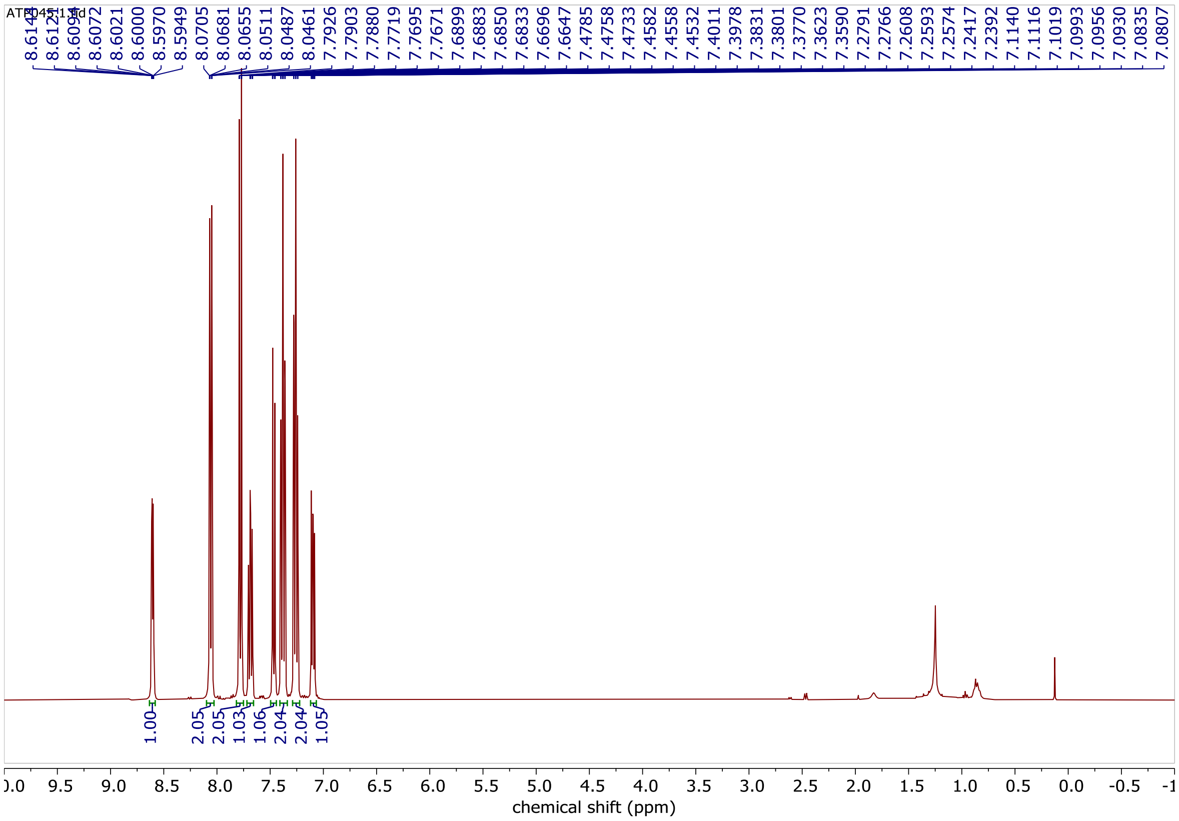

6.2 Intramolecular kinetic isotopic effect studies

Following the **GP**, a reaction was performed using *N*-(pyridin-2-yl)-9*H*-carbazole-d (**1a-D$_1$,** 49 mg, 0.2 mmol, 1.0 equiv) and silver nitrate (41 mg, 0.24, 1.2 equiv) at pre-heated oil bath at 120 °C for 2 h. Product **3a + 3a-D$_1$** was isolated by silica gel column chromatography using n-hexane/EtOAc (99:1) as the eluent in 10% yield and k_H/k_D value was calculated by the ^{1}H NMR analysis of the isolated compound **3a + 3a-D$_1$**.

Kinetic isotopic effect (KIE) = $k_H/k_D \approx 1.5$

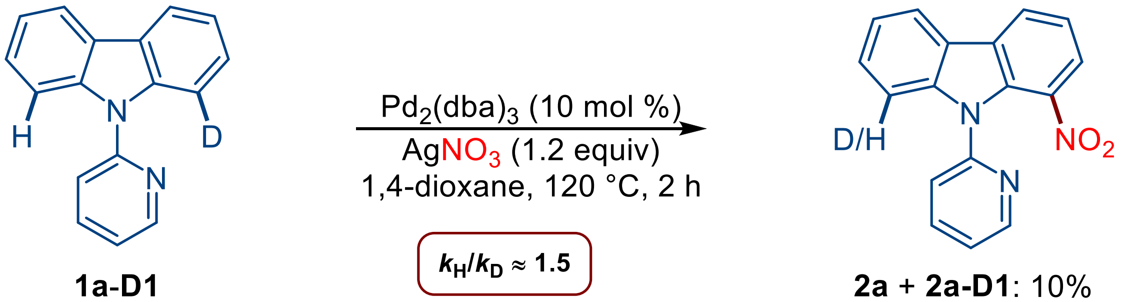

1a-D1 **2a + 2a-D1**: 10%

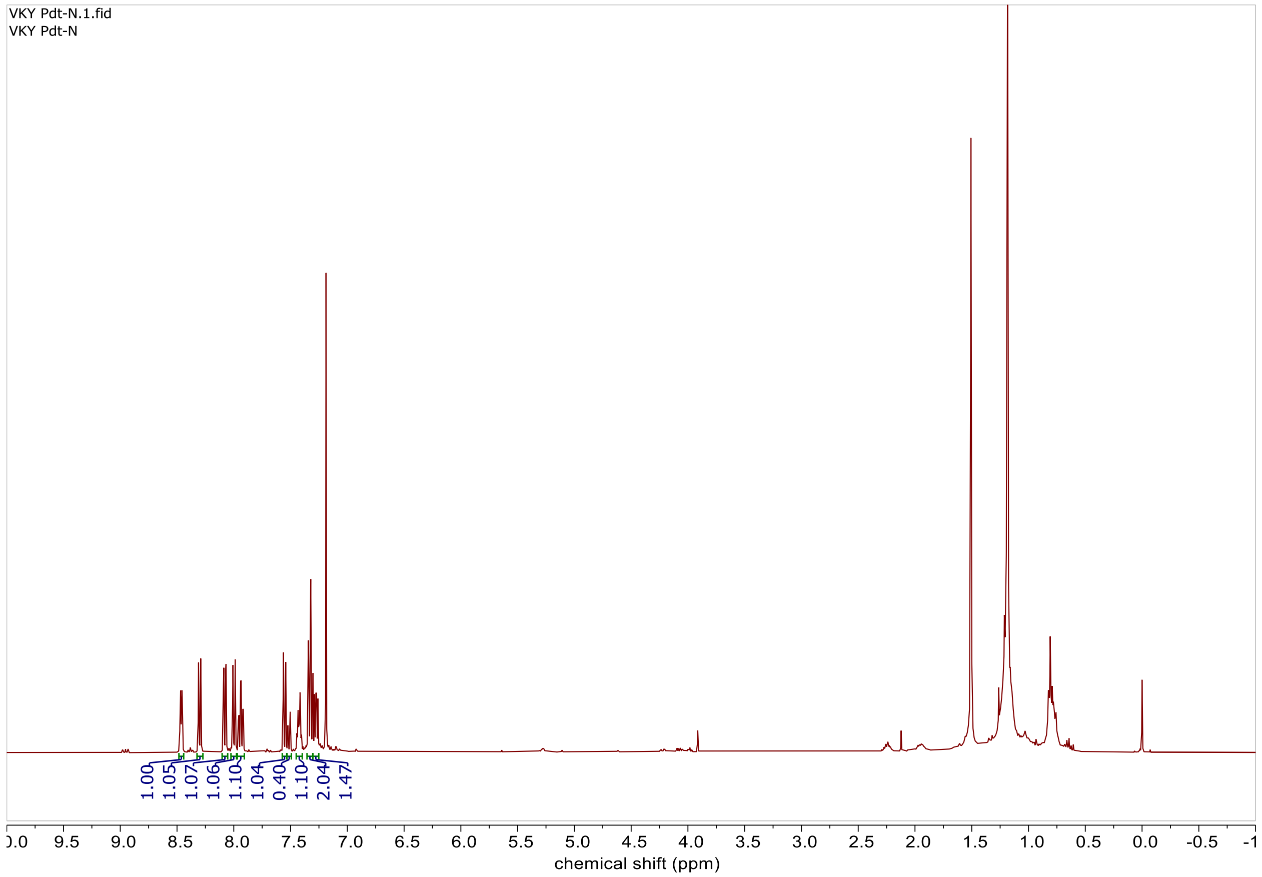

6.3 Isolation of palladacycle key intermediate (6) [1]

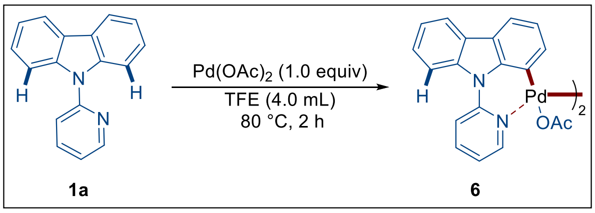

By following a reported procedure from our laboratory, a 15 mL Schlenk tube was charged with $Pd(OAc)_2$ (100 mg, 0.45 mmol, 1.0 equiv), *N*-(pyridin-2-yl)-9*H*-carbazole (**1a**, 109 mg, 0.45 mmol, 1.0 equiv), TFE (4.0 mL), and was stirred in preheated oil bath at 80 °C for 2 h. After completion of the reaction time, the solution was evaporated under reduced pressure and the crude residue was washed several times with Et_2O to remove the unreacted *N*-pyridyl carbazole, afforded the pure cyclometalated species **6** in 95% (173 mg) yield. The crystal seeding done by slow evaporation of DCM at room temperature.

6.4 Reaction with key palladacycle intermediate

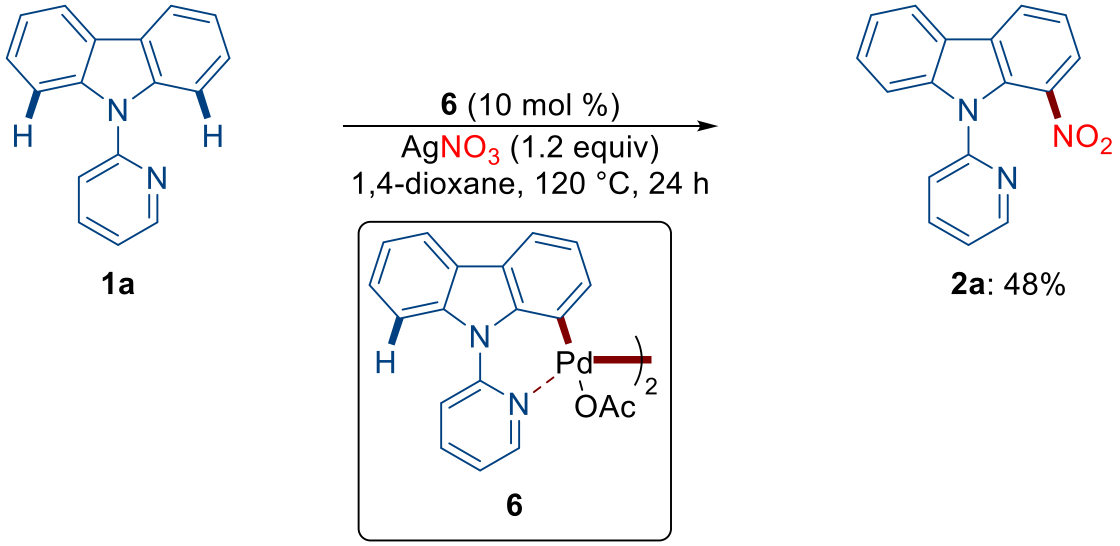

A 15 mL pressure tube was charged with palladacycle intermediate **6** (11 mg, 0.02 mmol, 10 mol %), *N*-(pyridin-2-yl)-9*H*-carbazole (**1a**, 45 mg, 0.2 mmol, 1.0 equiv), and $AgNO_3$ (41 mg, 0.24 mmol, 1.2 equiv). Then, 1,4-dioxane solvent (2.0 mL) was added, and the reaction mixture allowed to stir in pre-heated oil bath at 120 ºC for 24 h. On completion of reaction time, 10 mL of DCM was added for dilution of reaction mixture. The crude was filtered through a Celite pad, and the filtrate was concentrated using rotary evaporator. The crude residue was purified through a silica gel column chromatography using *n*-hexane/EtOAc (99:1) as eluent to give the pure product 1-nitro-9-(pyridin-2-yl)-9*H*-carbazole (**2a**) in 48% (28 mg) yield.

7. Comparative cyclic voltammetry (CV) studies of 9*H*-carbazole and 1-Nitro-9*H*-carbazole (3)

Cyclic voltammetry (CV) scans for 9*H*-carbazole and 1-nitro-9*H*-carbazole (**3**) vs Ag/AgCl. All data were collected with 20 mM of analyte concentration with tetrabutylammonium hexafluoro phosphate as the supporting electrolyte (100 mM) using anhydrous, degassed MeCN as the solvent (5.0 mL). The solution was purged with N_2 for 10 minutes prior to measurement to further minimize the dissolved oxygen. The CVs are recorded at 100 mV/s scan rate with a 3.0 mm GC disk electrode as working electrode, Pt as counter electrode and Ag/AgCl (3.0 M) as reference electrode. The potential range was scanned from -2.5 V to +2.5 V vs. Ag/AgCl.

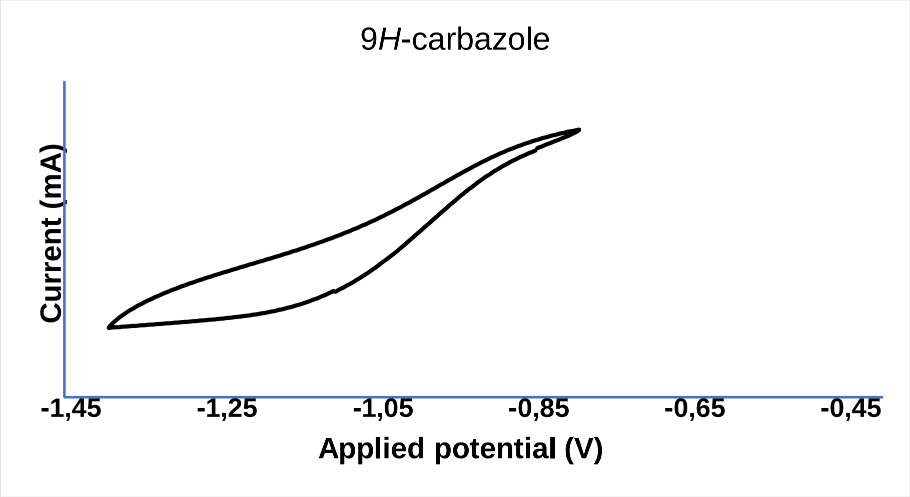

Figure S1. CV diagram of the 9*H*-carbazole.

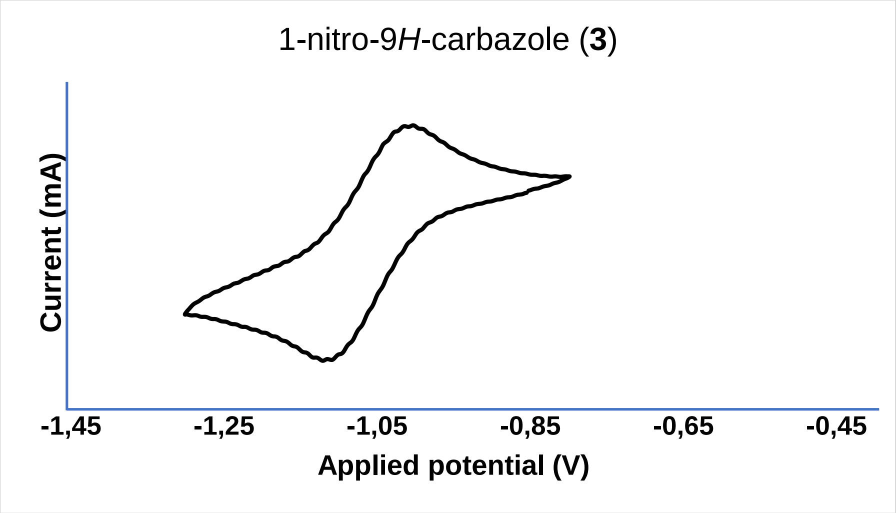

Figure S2. CV diagram of the 1-nitro-9*H*-carbazole.

8. Spectral data

1-Nitro-9-(pyridin-2-yl)-9*H*-carbazole (2a)

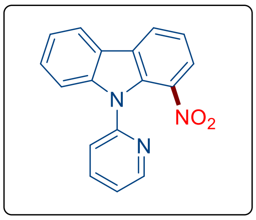

Compound **2a** was prepared according to the **GP-1** using 9-(pyridin-2-yl)-9*H*-carbazole (**1a**, 45 mg, 0.184 mmol, 1.0 equiv).

Appearance: Yellow solid

Yield: 69% (37 mg)

M. p.: 133-135 ºC

^{1}H NMR (400 MHz, $CDCl_3$): δ 8.53 (dd, *J* = 7.9, 2.0, Hz, 1H), 8.35 (dd, *J* = 7.7, 1.1 Hz, 1H), 8.17 – 8.10 (m, 1H), 8.06 (dd, *J* = 8.0, 1.1 Hz, 1H), 8.00 (dd, *J* = 7.7, 1.9 Hz, 1H), 7.62-7.56 (m, 2H), 7.49 (dd, *J* = 8.4, 1.3 Hz, 1H), 7.44 – 7.37 (m, 2H), 7.36 – 7.31 (m, 1H).

^{13}C{^{1}H} NMR (101 MHz, $CDCl_3$): δ 151.7 (C_q), 149.4 (*C*H), 141.7 (C_q), 138.7 (*C*H), 136.5 (C_q), 131.7 (C_q), 128.7 (C_q), 127.8 (*C*H), 125.5 (*C*H), 123.1 (C_q), 122.8 (*C*H), 122.2 (*C*H), 120.6 (*C*H), 120.3 (*C*H), 120.2 (*C*H), 119.9 (*C*H), 110.8 (*C*H).

HRMS (ESI-Q-TOF)**:** *m/z* $[M + H]^+$ calcd for $C_{17}H_{12}N_3O_2$ 290.0924; found 290.0923.

IR (ATR): 3073, 1589, 1519, 1470, 1339, 1277, 1189, 1087, 746, 429 cm^{-1}.

9-(3-Methylpyridin-2-yl)-1-nitro-9*H*-carbazole (2b)

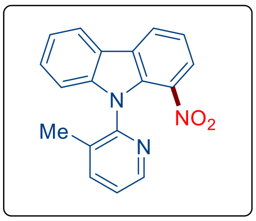

Compound **2b** was prepared according to the **GP-1** using 9-(3-methylpyridin-2-yl)-9*H*-carbazole (**1b**, 52 mg, 0.2 mmol, 1.0 equiv).

Appearance: Yellow solid

Yield: 53% (32 mg)

M. p.: 123-125 ºC

^{1}H NMR (400 MHz, $CDCl_3$): δ 8.38 (dd, *J* = 7.7, 1.1 Hz, 1H), 8.33 (dd, *J* = 4.9, 1.8 Hz, 1H), 8.15 (d, *J* = 7.8 Hz, 1H), 8.10 (dd, *J* = 8.1, 1.1 Hz, 1H), 7.86 (dd, *J* = 7.6, 0.9 Hz, 1H), 7.45 (dd, *J* = 8.3, 1.3 Hz, 1H), 7.41 – 7.28 (m, 3H), 7.02 (d, *J* = 8.2 Hz, 1H), 2.38 (s, 3H).

^{13}C{^{1}H} NMR (101 MHz, $CDCl_3$): δ 150.9 (C_q), 147.2 (C_q), 141.8 (C_q), 140.4 (*C*H), 135.8 (C_q), 132.9 (C_q), 130.9 (C_q), 128.8 (C_q), 127.7 (*C*H), 125.8 (*C*H), 123.9 (*C*H), 122.9 (*C*H), 122.8 (*C*H), 121.9 (*C*H), 120.6 (*C*H), 119.8 (*C*H), 111.1 (*C*H), 17.7 (CH_3).

HRMS (ESI-Q-TOF)**:** *m/z* $[M + H]^+$ calcd for $C_{18}H_{14}N_3O_2$ 304.1081; found 304.1083.

IR (ATR): 3034, 1663, 1529, 1487, 1335, 1237, 1129, 1057, 846, 579 cm^{-1}.

1-Nitro-9-(pyrimidin-2-yl)-9*H*-carbazole (2c)

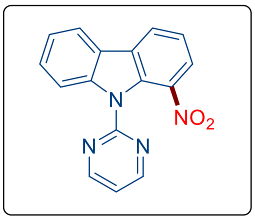

Compound **2c** was prepared according to the **GP-1** using 9-(pyrimidin-2-yl)-9*H*-carbazole (**1c**, 49 mg, 0.2 mmol, 1.0 equiv).

Appearance: Yellow solid

Yield: 10% (6 mg)

M. p.: 103-105 ºC

^{1}H NMR (400 MHz, CDCl$_3$): δ 8.81 (d, *J* = 4.8 Hz, 2H), 8.46 (d, *J* = 8.4 Hz, 1H), 8.34 (dd, *J* = 7.7, 1.1 Hz, 1H), 8.11 (dd, *J* = 7.9, 0.7 Hz, 1H), 8.09 – 8.05 (m, 1H), 7.56 (dd, *J* = 8.5, 1.3 Hz, 1H), 7.49 – 7.39 (m, 2H), 7.22 (t, *J* = 4.9 Hz, 1H).

^{13}C{^{1}H} NMR (101 MHz, CDCl$_3$): δ 158.1 (2*C*H), 141.1 (C_q), 130.8 (C_q), 129.5 (C_q), 129.2 (C_q), 128.2 (*C*H), 128.0 (C_q), 125.1 (*C*H), 123.9 (C_q), 123.1 (*C*H), 122.5 (*C*H), 121.5 (*C*H), 120.1 (*C*H), 118.3 (*C*H), 113.9 (*C*H).

HRMS (ESI-Q-TOF)**:** *m/z* [M + H]$^+$ calcd for $C_{16}H_{11}N_4O_2$ 291.0877; found 291.0878.

IR (ATR): 3039, 1633, 1549, 1467, 1315, 1217, 1139, 1027, 956, 469 cm^{-1}.

9-(Isoquinolin-1-yl)-1-nitro-9*H*-carbazole (2d)

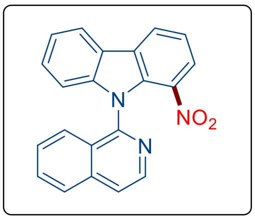

Compound **2d** was prepared according to the **GP-1** using 9-(isoquinolin-1-yl)-9*H*-carbazole (**1d**, 59 mg, 0.2 mmol, 1.0 equiv).

Appearance: Yellow solid

Yield: 60% (41 mg)

M. p.: 162-164 ºC

^{1}H NMR (400 MHz, CDCl$_3$): δ 8.34 (dd, *J* = 7.7, 1.2 Hz, 1H), 8.28 (d, *J* = 5.7 Hz, 1H), 8.11 – 8.07 (m, 1H), 8.03 (dd, *J* = 8.0, 1.2 Hz, 1H), 7.95 – 7.88 (m, 2H), 7.75 – 7.65 (m, 2H), 7.51 (dd, *J* = 8.3, 1.2 Hz, 1H), 7.36 – 7.21 (m, 3H), 6.89 – 6.80 (m, 1H).

^{13}C{^{1}H} NMR (101 MHz, CDCl$_3$): δ 151.3 (C_q), 142.9 (C_q), 141.5 (*C*H), 138.4 (C_q), 135.9 (C_q), 133.7 (C_q), 131.2 (*C*H), 129.1 (C_q), 128.4 (*C*H), 127.6 (*C*H), 127.4 (*C*H), 125.9 (*C*H),

125.4 (*C*H), 125.2 (C_q), 123.1 (C_q), 123.0 (*C*H), 122.1 (*C*H), 121.8 (*C*H), 120.5 (*C*H), 120.3 (*C*H), 112.0 (*C*H).

HRMS (ESI-Q-TOF)**:** *m/z* $[M + H]^+$ calcd for $C_{21}H_{14}N_3O_2$ 340.1081; found 340.1084.

IR (ATR): 3044, 1673, 1539, 1437, 1367, 1287, 1149, 1077, 976, 389 cm^{-1}.

3,6-Dimethoxy-1-nitro-9-(pyridin-2-yl)-9*H*-carbazole (2j)

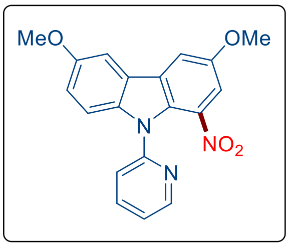

Compound **2j** was prepared according to the **GP-1** using 3,6-dimethoxy-9-(pyridin-2-yl)-9*H*-carbazole (**1j**, 61 mg, 0.2 mmol, 1.0 equiv).

Appearance: Yellow solid

Yield: 60% (42 mg)

M. p.: 143-145 °C

^{1}H NMR (400 MHz, $CDCl_3$): δ 8.48 (dd, *J* = 4.9, 2.0 Hz, 1H), 7.95 (dd, *J* = 7.9, 2.0 Hz, 1H), 7.80 (d, *J* = 2.5 Hz, 1H), 7.65 (d, *J* = 2.5 Hz, 1H), 7.54 (d, *J* = 7.9 Hz, 1H), 7.53 – 7.42 (m, 2H), 7.35 – 7.23 (m, 1H), 7.07 (dd, *J* = 9.0, 2.6 Hz, 1H), 3.96 (s, 3H), 3.92 (s, 3H).

$^{13}C\{^1H\}$ NMR (101 MHz, $CDCl_3$): δ 155.5 (C_q), 153.5 (C_q), 152.1 (C_q), 149.2 (*C*H), 138.6 (*C*H), 137.2 (C_q), 136.1 (C_q), 129.6 (C_q), 127.5 (C_q), 123.6 (C_q), 122.2 (*C*H), 119.3 (*C*H), 116.8 (*C*H), 111.9 (*C*H), 110.7 (*C*H), 109.5 (*C*H), 103.1 (*C*H), 56.5 (CH_3), 55.9 (CH_3).

HRMS (ESI-Q-TOF)**:** *m/z* $[M + H]^+$ calcd for $C_{19}H_{16}N_3O_4$ 350.1135; found 350.1139.

IR (ATR): 3053, 1571, 1533, 1416, 1379, 1214, 1156, 1027, 946, 529 cm^{-1}.

3,6-Di-*tert*-butyl-1-nitro-9-(pyridin-2-yl)-9*H*-carbazole (2k)

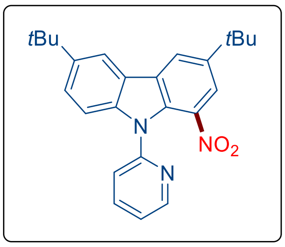

Compound **2k** was prepared according to the **GP-1** using 3,6-di-*tert*-butyl-9-(pyridin-2-yl)-9*H*-carbazole (**1k**, 71 mg, 0.2 mmol, 1.0 equiv).

Appearance: Yellow solid

Yield: 55% (44 mg)

M. p.: 153-155 ºC

^{1}H NMR (400 MHz, $CDCl_3$): δ 8.50 (dd, *J* = 4.9, 1.9 Hz, 1H), 8.37 (d, *J* = 1.9 Hz, 1H), 8.12 (d, *J* = 1.1 Hz, 1H), 8.11 (d, *J* = 1.8 Hz, 1H), 7.98 (dd, *J* = 7.7, 1.9 Hz, 1H), 7.59 (d, *J* = 8.0 Hz, 1H), 7.57 – 7.49 (m, 2H), 7.29 (dd, *J* = 7.4, 4.9 Hz, 1H), 1.49 (s, 9H), 1.45 (s, 9H).

^{13}C{^{1}H} NMR (101 MHz, $CDCl_3$): δ 152.1 (C_q), 149.3 (*C*H), 145.3 (C_q), 143.9 (C_q), 143.4 (C_q), 140.2 (C_q), 138.6 (*C*H), 128.9 (C_q), 128.4 (C_q), 125.5 (*C*H), 123.1 (C_q), 122.3 (*C*H), 122.1 (*C*H), 120.2 (*C*H), 119.5 (*C*H), 116.5 (*C*H), 110.4 (*C*H), 34.9 (C_q), 34.8 (C_q), 31.9 (3CH_3), 31.7 (3CH_3).

HRMS (ESI-Q-TOF)**:** *m/z* $[M + H]^+$ calcd for $C_{25}H_{28}N_3O_2$ 402.2176; found 402.2182.

IR (ATR): 3059, 1576, 1523, 1476, 1351, 1274, 1186, 1077, 846, 579 cm^{-1}.

The spectral data are in accordance with the reported literature data.[8]

1-Nitro-3,6-diphenyl-9-(pyridin-2-yl)-9*H*-carbazole (2l)

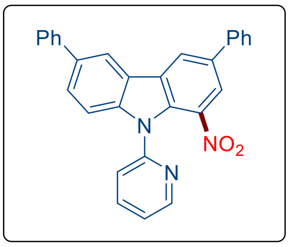

Compound **2l** was prepared according to the **GP-1** using 3,6-diphenyl-9-(pyridin-2-yl)-9*H*-carbazole (**1l**, 79 mg, 0.2 mmol, 1.0 equiv).

Appearance: Yellow solid

Yield: 48% (42 mg)

M. p.: 163-165 ºC

^{1}H NMR (400 MHz, CDCl$_3$): δ 8.61 (s, 1H), 8.56 (dd, *J* = 5.0, 1.9 Hz, 1H), 8.38 (s, 1H), 8.32 (s, 1H), 8.03 (dd, *J* = 7.7, 1.9 Hz, 1H), 7.77 – 7.71 (m, 3H), 7.72 – 7.61 (m, 4H), 7.54 (d, *J* = 7.5 Hz, 2H), 7.50 (dd, *J* = 6.9, 6.4, 2.0 Hz, 2H), 7.46 – 7.33 (m, 3H).

^{13}C{^{1}H} NMR (101 MHz, CDCl$_3$): δ 151.8 (C$_q$), 149.6 (*C*H), 141.7 (C$_q$), 141.2 (C$_q$), 139.5 (C$_q$), 138.9 (*C*H), 136.8 (C$_q$), 136.0 (C$_q$), 134.4 (C$_q$), 131.5 (C$_q$), 129.6 (C$_q$), 129.3 (2*C*H), 129.1 (2*C*H), 127.9 (*C*H), 127.5 (2*C*H), 127.4 (2*C*H), 127.3 (*C*H), 127.3 (*C*H), 123.9 (C$_q$), 123.8 (*C*H), 122.9 (*C*H), 121.9 (*C*H), 119.8 (*C*H), 119.1 (*C*H), 111.4 (*C*H).

HRMS (ESI-Q-TOF)**:** *m/z* [M + H]$^+$ calcd for $C_{29}H_{20}N_3O_2$ 442.1550; found 442.1556.

IR (ATR): 3069, 1676, 1533, 1466, 1361, 1284, 1116, 1047, 646, 539 cm^{-1}.

6-Nitro-7-(pyridin-2-yl)-7*H*-dibenzo[*c*,*g*]carbazole (2m)

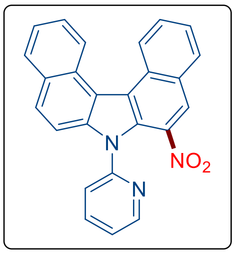

Compound **2m** was prepared according to the **GP-1** using 7-(pyridin-2-yl)-7*H*-dibenzo[*c*,*g*]carbazole (**1m**, 69 mg, 0.2 mmol, 1.0 equiv).

Appearance: Yellow solid

Yield: 55% (43 mg)

M. p.: 128-130 ºC

^{1}H NMR (400 MHz, $CDCl_3$): δ 9.19 (d, *J* = 8.6 Hz, 1H), 9.04 (d, *J* = 8.4 Hz, 1H), 8.58 (dd, *J* = 5.0, 1.9 Hz, 1H), 8.52 (s, 1H), 8.12 (dd, *J* = 8.1, 1.4 Hz, 1H), 8.07-8.02 (m, 2H), 7.96 – 7.88 (m, 1H), 7.82 – 7.74 (m, 2H), 7.73 – 7.67 (m, 2H), 7.66 – 7.52 (m, 2H), 7.48 – 7.37 (m, 1H).

^{13}C{^{1}H} NMR (101 MHz, $CDCl_3$): δ 151.4 (C_q), 149.8 (*C*H), 138.9 (*C*H), 138.8 (C_q), 130.8 (C_q), 130.6 (C_q), 130.5 (*C*H), 129.2 (*C*H), 128.7 (*C*H), 128.6 (*C*H), 128.5 (C_q), 128.1 (C_q), 127.0 (*C*H), 125.9 (*C*H), 125.4 (*C*H), 125.4 (*C*H), 124.4 (*C*H), 123.9 (*C*H), 123.7 (C_q), 123.3 (C_q), 123.3 (*C*H), 120.5 (*C*H), 117.6 (C_q), 112.2 (C_q), 111.6 (*C*H).

HRMS (ESI-Q-TOF)**:** *m/z* $[M + H]^+$ calcd for $C_{25}H_{16}N_3O_2$ 390.1237; found 390.1231.

IR (ATR): 3059, 1637, 1543, 1426, 1327, 1287, 1152, 1027, 742, 529 cm^{-1}.

1-Nitro-7-phenyl-9-(pyridin-2-yl)-9*H*-carbazole (2n)

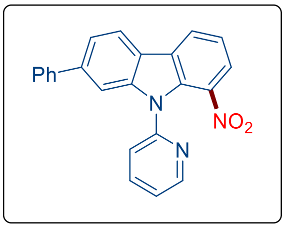

Compound **2n** was prepared according to the **GP-1** using 2-phenyl-9-(pyridin-2-yl)-9*H*-carbazole (**1n**, 64 mg, 0.2 mmol, 1.0 equiv).

Appearance: Yellow solid

Yield: 53% (38 mg)

M. p.: 127-129 ºC

^{1}H NMR (400 MHz, CDCl$_3$): δ 8.56 (dd, *J* = 4.9, 2.0 Hz, 1H), 8.35 (dd, *J* = 7.7, 1.1 Hz, 1H), 8.17 (d, *J* = 8.1 Hz, 1H), 8.06 (dd, *J* = 8.1, 1.1 Hz, 1H), 8.01 (dd, *J* = 7.7, 1.9 Hz, 1H), 7.74 (s, 1H), 7.68 – 7.58 (m, 4H), 7.53 – 7.43 (m, 2H), 7.43 – 7.31 (m, 3H).

^{13}C{^{1}H} NMR (101 MHz, CDCl$_3$): δ 151.8 (C_q), 149.6 (*C*H), 142.4 (C_q), 141.5 (C_q), 141.4 (C_q), 138.9 (*C*H), 136.5 (C_q), 132.3 (C_q), 128.9 (2*C*H), 128.6 (C_q), 127.7 (*C*H), 127.7 (2*C*H), 125.5 (*C*H), 122.9 (*C*H), 122.8 (*C*H), 122.3 (C_q), 122.0 (*C*H), 120.9 (*C*H), 120.4 (*C*H), 120.0 (*C*H), 109.4 (*C*H).

HRMS (ESI-Q-TOF)**:** *m/z* $[M + H]^+$ calcd for $C_{23}H_{16}N_3O_2$ 366.1237; found 366.1241.

IR (ATR): 3043, 1569, 1509, 1477, 1319, 1274, 1159, 1057, 733, 414 cm^{-1}.

7-Methoxy-1-nitro-9-(pyridin-2-yl)-9*H*-carbazole (2o)

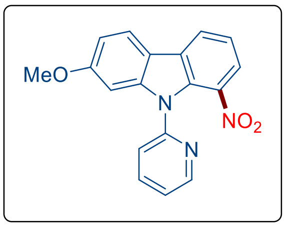

Compound **2o** was prepared according to the **GP-1** using 2-methoxy-9-(pyridin-2-yl)-9*H*-carbazole (**1o**, 55 mg, 0.2 mmol, 1.0 equiv).

Appearance: Yellow solid

Yield: 37% (24 mg)

M. p.: 137-139 ºC

^{1}H NMR (400 MHz, $CDCl_3$): δ 8.55 (dd, *J* = 4.9, 2.0 Hz, 1H), 8.24 (dd, *J* = 7.7, 1.1 Hz, 1H), 8.06 – 7.92 (m, 3H), 7.57 (d, *J* = 8.0 Hz, 1H), 7.40 – 7.28 (m, 2H), 7.05 (d, *J* = 7.8 Hz, 1H), 6.98 (dd, *J* = 8.6, 2.2 Hz, 1H), 3.86 (s, 3H).

^{13}C{^{1}H} NMR (101 MHz, $CDCl_3$): δ 160.4 (C_q), 151.8 (C_q), 149.5 (*C*H), 143.2 (C_q), 138.8 (*C*H), 136.3 (C_q), 131.8 (C_q), 128.9 (C_q), 124.5 (*C*H), 122.7 (*C*H), 121.4 (*C*H), 121.3 (*C*H), 120.3 (*C*H), 119.7 (*C*H), 116.7 (C_q), 110.4 (*C*H), 95.5 (*C*H), 55.7 (*C*H_3).

HRMS (ESI-Q-TOF)**:** *m/z* $[M + H]^+$ calcd for $C_{18}H_{14}N_3O_3$ 320.1030; found 320.1035.

IR (ATR): 3057, 2929, 1637, 1589, 1432, 1387, 1277, 1128, 764, 552 cm^{-1}.

7-Chloro-1-nitro-9-(pyridin-2-yl)-9*H*-carbazole (2p)

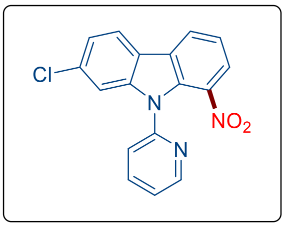

Compound **2p** was prepared according to the **GP-1** using 2-chloro-9-(pyridin-2-yl)-9*H*-carbazole (**1p**, 58 mg, 0.2 mmol, 1.0 equiv).

Appearance: Yellow solid

Yield: 47% (30 mg)

M. p.: 107-109 ºC

^{1}H NMR (400 MHz, CDCl$_3$): δ 8.54 (dd, *J* = 4.9, 2.0 Hz, 1H), 8.29 (dd, *J* = 7.7, 1.1 Hz, 1H), 8.11 – 7.97 (m, 3H), 7.58 (s, 1H), 7.57 – 7.53 (m, 1H), 7.44 – 7.31 (m, 3H).

^{13}C{^{1}H} NMR (101 MHz, CDCl$_3$): δ 151.3 (C_q), 149.7 (*C*H), 142.3 (C_q), 139.1 (*C*H), 136.6 (C_q), 133.7 (C_q), 132.0 (C_q), 128.1 (C_q), 125.5 (*C*H), 123.2 (*C*H), 123.1 (*C*H), 122.9 (*C*H), 121.7 (C_q), 121.5 (*C*H), 120.8 (*C*H), 119.8 (*C*H), 111.3 (*C*H).

HRMS (ESI-Q-TOF)**:** *m/z* $[M + H]^+$ calcd for $C_{17}H_{11}ClN_3O_2$ 324.0534; found 324.0533.

IR (ATR): 3027, 2928, 1677, 1579, 1422, 1377, 1267, 1138, 754, 452 cm^{-1}.

6-Chloro-1-nitro-9-(pyridin-2-yl)-9*H*-carbazole (2q)

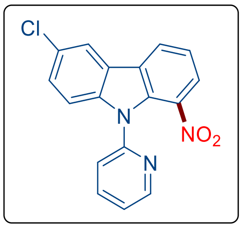

Compound **2q** was prepared according to the **GP-1** using 2-chloro-9-(pyridin-2-yl)-9*H*-carbazole (**1q**, 58 mg, 0.2 mmol, 1.0 equiv).

Appearance: Yellow solid

Yield: 37% (24 mg)

M. p.: 111-113 ºC

^{1}H NMR (400 MHz, CDCl$_3$): δ 8.53 (dd, *J* = 4.9, 2.0 Hz, 1H), 8.32 – 8.26 (m, 1H), 8.15 – 8.03 (m, 2H), 8.03 – 7.97 (m, 1H), 7.60 – 7.52 (m, 1H), 7.49 (d, *J* = 8.8 Hz, 1H), 7.45 – 7.38 (m, 2H), 7.35 (dd, *J* = 7.5, 4.9 Hz, 1H).

^{13}C{^{1}H} NMR (101 MHz, CDCl$_3$): δ 151.3 (C_q), 149.7 (*C*H), 142.3 (C_q), 139.1 (*C*H), 136.6 (C_q), 133.7 (C_q), 132.0 (C_q), 128.1 (C_q), 125.5 (*C*H), 123.2 (*C*H), 123.1 (*C*H), 122.9 (*C*H), 121.7 (C_q), 121.5 (*C*H), 120.8 (*C*H), 119.8 (*C*H), 111.3 (*C*H).

HRMS (ESI-Q-TOF)**:** *m/z* $[M + H]^+$ calcd for $C_{17}H_{11}ClN_3O_2$ 324.0534; found 324.0537.

IR (ATR): 3029, 2948, 1657, 1519, 1472, 1357, 1292, 1168, 954, 652 cm^{-1}.

6-Bromo-1-nitro-9-(pyridin-2-yl)-9*H*-carbazole (2r)

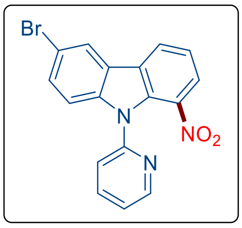

Compound **2r** was prepared according to the **GP-1** using 3-bromo-9-(pyridin-2-yl)-9*H*-carbazole (**1r**, 65 mg, 0.2 mmol, 1.0 equiv).

Appearance: Yellow solid

Yield: 31% (23 mg)

M. p.: 121-123 ºC

^{1}H NMR (400 MHz, CDCl$_3$): δ 8.53 (dd, *J* = 4.9, 1.9 Hz, 1H), 8.30 (dd, *J* = 7.7, 1.1 Hz, 1H), 8.24 (s, 1H), 8.08 (dd, *J* = 8.0, 1.1 Hz, 1H), 8.00 (d, *J* = 7.8, 1H), 7.60 – 7.51 (m, 2H), 7.48 – 7.39 (m, 2H), 7.39 – 7.33 (m, 1H).

^{13}C{^{1}H} NMR (101 MHz, CDCl$_3$): δ 151.4 (C_q), 149.6 (*C*H), 140.5 (C_q), 139.0 (*C*H), 136.6 (C_q), 132.0 (C_q), 130.6 (*C*H), 127.6 (C_q), 125.8 (*C*H), 124.9 (C_q), 123.6 (*C*H), 123.4 (*C*H), 123.1 (*C*H), 120.7 (*C*H), 119.8 (*C*H), 115.2 (C_q), 112.5 (*C*H).

HRMS (ESI-Q-TOF)**:** *m/z* $[M + H]^+$ calcd for $C_{17}H_{11}BrN_3O_2$ 368.0029; found 368.0032.

IR (ATR): 3057, 2958, 1667, 1517, 1452, 1357, 1227, 1178, 986, 752 cm^{-1}.

8-Nitro-7-(pyridin-2-yl)-7*H*-benzo[*c*]carbazole (2s)

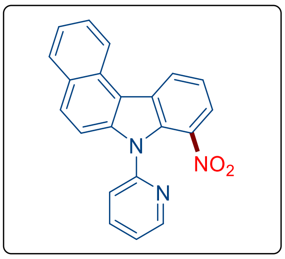

Compound **2s** was prepared according to the **GP-1** using 7-(pyridin-2-yl)-7*H*-benzo[*c*]carbazole (**1s**, 59 mg, 0.2 mmol, 1.0 equiv).

Appearance: Yellow solid

Yield: 36% (22 mg)

M. p.: 103-105 ºC

^{1}H NMR (400 MHz, $CDCl_3$): δ 8.86 (dd, *J* = 8.1, 1.1 Hz, 1H), 8.77 (dd, *J* = 8.5, 1.0 Hz, 1H), 8.59 (dd, *J* = 4.9, 2.0 Hz, 1H), 8.06 (dd, *J* = 8.0, 1.1 Hz, 1H), 8.03-7.99 (m, 2H), 7.89 (d, *J* = 9.0 Hz, 1H), 7.76 (dd, *J* = 8.4, 6.9 Hz, 1H), 7.68 (d, *J* = 9.0 Hz, 1H), 7.63 (d, *J* = 7.9 Hz, 1H), 7.55 (dd, *J* = 8.1, 6.9 Hz, 1H), 7.49 (d, *J* = 8.0 Hz, 1H), 7.39 (dd, *J* = 7.5, 1.1 Hz, 1H).

^{13}C{^{1}H} NMR (101 MHz, $CDCl_3$): δ 151.6 (C_q), 149.7 (*C*H), 139.9 (C_q), 138.9 (*C*H), 136.8 (C_q), 130.9 (C_q), 130.4 (C_q), 129.6 (C_q), 129.4 (C_q), 129.3 (*C*H), 129.2 (*C*H), 127.8 (*C*H), 127.4 (*C*H), 124.4 (*C*H), 123.3 (*C*H), 123.2 (*C*H), 121.4 (*C*H), 120.7 (*C*H), 120.6 (*C*H), 115.8 (C_q), 111.7 (*C*H).

HRMS (ESI-Q-TOF)**:** *m/z* $[M + H]^+$ calcd for $C_{21}H_{14}N_3O_2$ 340.1081; found 340.1085.

IR (ATR): 3059, 1646, 1583, 1496, 1341, 1277, 1156, 1087, 946, 439 cm^{-1}.

6-Nitro-7-(pyridin-2-yl)-7*H*-benzo[*c*]carbazole (2s')

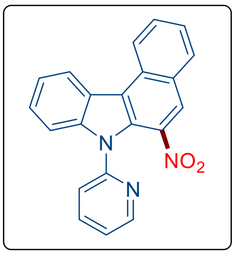

Compound **2s'** was prepared according to the **GP-1** using 7-(pyridin-2-yl)-7*H*-benzo[*c*]carbazole (**1s**, 59 mg, 0.2 mmol, 1.0 equiv).

Appearance: Yellow solid

Yield: 15% (10 mg)

M. p.: 108-110 ºC

^{1}H NMR (400 MHz, CDCl$_3$): δ 8.89 (d, *J* = 8.5 Hz, 1H), 8.69 – 8.61 (m, 1H), 8.54 (dd, *J* = 4.9, 1.9 Hz, 1H), 8.51 (s, 1H), 8.10 (dd, *J* = 8.3, 1.3 Hz, 1H), 8.04 (dd, *J* = 7.7, 1.9 Hz, 1H), 7.86 (dd, *J* = 8.4, 1.4 Hz, 1H), 7.79 – 7.67 (m, 2H), 7.61 (dd, *J* = 8.2, 1.1 Hz, 1H), 7.53-7.50 (m, 2H), 7.40 – 7.33 (m, 1H).

^{13}C{^{1}H} NMR (101 MHz, CDCl$_3$): δ 151.8 (C$_q$), 149.6 (*C*H), 140.7 (C$_q$), 138.8 (*C*H), 137.5 (C$_q$), 131.4 (C$_q$), 130.7 (*C*H), 130.1 (*C*H), 128.5 (C$_q$), 127.8 (C$_q$), 126.3 (*C*H), 125.2 (*C*H), 124.4 (*C*H), 123.9 (C$_q$), 123.5 (*C*H), 123.4 (*C*H), 122.9 (*C*H), 122.5 (*C*H), 121.4 (C$_q$), 120.0 (*C*H), 111.1 (*C*H).

HRMS (ESI-Q-TOF)**:** *m/z* [M + H]$^+$ calcd for $C_{21}H_{14}N_3O_2$ 340.1081; found 340.1085.

IR (ATR): 3049, 1647, 1563, 1476, 1321, 1267, 1155, 1077, 942, 429 cm^{-1}.

1-Nitro-9*H*-carbazole (3)

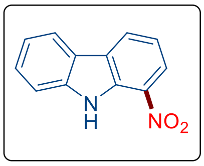

Compound **3** was prepared according to the mentioned procedure in section **5.1** using 1-nitro-9-(pyridin-2-yl)-9*H*-carbazole (**2a**, 29 mg, 0.1 mmol, 1.0 equiv).

Appearance: Yellow solid

Yield: 53% (11 mg)

M. p.: 193-195 ºC

^{1}H NMR (400 MHz, $CDCl_3$): δ 10.00 (s, N*H*), 8.36-8.32 (m, 2H), 8.10 (d, *J* = 7.9 Hz, 1H), 7.64 – 7.48 (m, 2H), 7.39 – 7.24 (m, 2H).

^{13}C{^{1}H} NMR (101 MHz, $CDCl_3$): δ 139.8 (C_q), 133.7 (C_q), 132.1 (C_q), 127.7 (*C*H), 127.5 (*C*H), 127.4 (C_q), 122.2 (C_q), 121.9 (*C*H), 121.3 (*C*H), 120.7 (*C*H), 118.7 (*C*H), 111.7 (*C*H).

HRMS (ESI-Q-TOF)**:** *m/z* $[M + H]^+$ calcd for $C_{12}H_9N_2O_2$ 213.0659; found 213.0660.

IR (ATR): 2942, 2863, 2146, 1454, 1423, 1319, 1231, 881, 628, 565 cm^{-1}.

The spectral data are in accordance with the reported literature data.[9]

9-(Pyridin-2-yl)-9*H*-carbazol-1-amine (4)

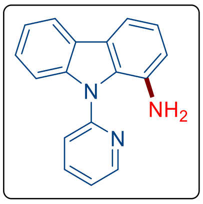

Compound **4** was prepared according to the mentioned procedure in section **5.2** using 1-nitro-9-(pyridin-2-yl)-9*H*-carbazole (**2a**, 29 mg, 0.1 mmol, 1.0 equiv).

Appearance: Yellow solid

Yield: 88% (23 mg) Condition A, 93% (24 mg) Condition B, 85% (22 mg) Condition C

M. p.: 113-115 °C

^{1}H NMR (400 MHz, $CDCl_3$): δ 8.60 (dd, *J* = 4.9, 2.0 Hz, 1H), 8.04 – 7.91 (m, 1H), 7.82 (dd, *J* = 8.0, 2.0 Hz, 1H), 7.55 (dd, *J* = 7.7, 1.1 Hz, 1H), 7.47-7.41 (m, 2H), 7.32 – 7.21 (m, 4H), 7.19 (dd, *J* = 7.2, 1.0 Hz, 1H), 7.09 (t, *J* = 7.7 Hz, 1H), 6.72 (dd, *J* = 7.6, 1.1 Hz, 1H).

^{13}C{^{1}H} NMR (101 MHz, $CDCl_3$): δ 150.2 (C_q), 147.3 (*C*H), 139.2 (C_q), 136.5 (*C*H), 131.4 (C_q), 126.7 (C_q), 124.5 (C_q), 124.2 (*C*H), 122.9 (C_q), 120.2 (*C*H), 120.0 (*C*H), 119.9 (*C*H), 119.1 (*C*H), 118.5 (*C*H), 112.6 (*C*H), 109.2 (*C*H), 108.3 (*C*H).

HRMS (ESI-Q-TOF)**:** *m/z* $[M + H]^+$ calcd for $C_{17}H_{14}N_3$ 260.1182; found 260.1195.

IR (ATR): 3339, 3292, 2936, 1678, 1424, 1258, 1120, 666, 534 cm^{-1}.

9*H*-carbazol-1-amine (5)

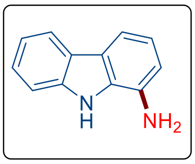

Compound **5** was prepared according to the mentioned procedure in section **5.3** using 1-nitro-9*H*-carbazole (**3**, 21 mg, 0.1 mmol, 1.0 equiv).

Appearance: Dark brown sticky solid

Yield: 90% (16 mg)

M. p.: 123-125 ºC

^{1}H NMR (400 MHz, $CDCl_3$): δ 8.09 (dd, *J* = 7.8, 0.9 Hz, 1H), 8.05 (d, *J* = 7.8 Hz, 1H), 7.87 (s, 1H), 7.53 (d, *J* = 7.9 Hz, 1H), 7.40 – 7.25 (m, 3H), 7.25 – 7.09 (m, 1H), 7.02 (dd, *J* = 7.7, 1.4 Hz, 1H), 6.75 (d, *J* = 7.5 Hz, 1H).

^{13}C{^{1}H} NMR (101 MHz, $CDCl_3$): δ 139.7 (C_q), 130.3 (C_q), 125.8 (*C*H), 125.7 (*C*H), 124.3 (C_q), 123.3 (C_q), 120.6 (C_q), 120.3 (*C*H), 119.6 (*C*H), 119.4 (*C*H), 112.2 (*C*H), 111.1 (*C*H).

HRMS (ESI-Q-TOF)**:** *m/z* $[M + H]^+$ calcd for $C_{12}H_{11}N_2$ 183.0917; found 183.0924.

IR (ATR): 3335, 3282, 2956, 1698, 1454, 1288, 1122, 696, 434 cm^{-1}.

The spectral data are in accordance with the reported literature data.[10,11]

9. Crystal X-ray diffraction data of compound 2a (CCDC 2478298)

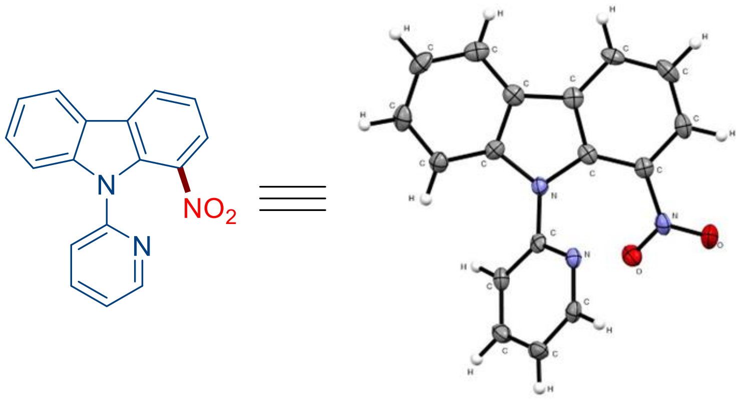

Table S9. Crystal data and structure refinement for pg1_120k_rep_0ma.

Identification code	pg1_120k_rep_0ma
Empirical formula	$C_{17}H_{11}N_3O_2$
Formula weight	289.296
Temperature/K	120.00
Crystal system	monoclinic
Space group	$P2_1/c$
a/Å	10.8187(6)
b/Å	12.0444(7)
c/Å	10.7835(6)
α/°	90
β/°	105.219(2)
γ/°	90
Volume/Å^3	1355.86(13)
Z	4
ρ_{calc}g/cm^3	1.417
μ/mm^{-1}	0.096
F(000)	600.4
Crystal size/mm^3	0.07 × 0.06 × 0.05
Radiation	Mo Kα (λ = 0.71073)
2Θ range for data collection/°	5.16 to 52.92
Index ranges	$-13 \le h \le 13$, $-15 \le k \le 15$, $-13 \le l \le 13$

Reflections collected	29150
Independent reflections	2788 [R_{int} = 0.0811, R_{sigma} = 0.0383]
Data/restraints/parameters	2788/0/210
Goodness-of-fit on F^2	1.063
Final R indexes [I>=2σ (I)]	R_1 = 0.0952, wR_2 = 0.1823
Final R indexes [all data]	R_1 = 0.1054, wR_2 = 0.1890
Largest diff. peak/hole / e Å^{-3}	0.57/-0.60

Table S10. Fractional atomic coordinates (×10^4) and equivalent isotropic displacement parameters (Å^2×10^3) for pg1_120k_rep_0ma. U_{eq} is defined as 1/3 of the trace of the orthogonalised U_{IJ} tensor.

**Atom**	***x***	***y***	***z***	**U(eq)**
O(1)	7790(3)	3172(2)	1193(3)	26.5(6)
O(2)	7967(3)	4311(3)	-303(3)	40.1(8)
N(1)	7263(3)	4422(3)	3413(3)	20.0(7)
N(2)	5592(3)	4289(3)	1549(3)	19.6(7)
N(3)	8051(3)	4086(3)	827(3)	23.9(7)
C(13)	6197(3)	3819(3)	2656(3)	19.1(7)
C(14)	5851(4)	2805(3)	3087(4)	22.5(8)
C(7)	8279(3)	5646(3)	4973(4)	22.6(8)
C(16)	4181(4)	2718(3)	1151(4)	27.0(9)
C(6)	8812(3)	5757(3)	3884(4)	21.0(8)
C(12)	7324(3)	4834(3)	4652(3)	20.3(8)
C(1)	8163(3)	5001(3)	2948(4)	19.6(8)
C(5)	9799(4)	6426(3)	3685(4)	24.7(8)
C(8)	8518(4)	6186(3)	6154(4)	26.6(9)
C(15)	4818(4)	2261(3)	2315(4)	26.7(9)
C(2)	8527(3)	4937(3)	1786(4)	21.0(8)
C(17)	4604(3)	3727(3)	804(4)	22.7(8)
C(4)	10116(4)	6356(3)	2531(4)	27.2(9)
C(3)	9471(4)	5636(3)	1585(4)	25.6(8)
C(11)	6588(4)	4542(3)	5487(4)	23.8(8)
C(9)	7801(4)	5902(4)	6993(4)	30.2(9)
C(10)	6847(4)	5090(4)	6655(4)	29.5(9)

Table S11. Anisotropic displacement parameters ($Å^2×10^3$) for pg1_120k_rep_0ma. The anisotropic displacement factor exponent takes the form: -$2\pi^2[h^2a^{*2}U_{11}+2hka^*b^*U_{12}+…]$.

Atom	U_{11}	U_{22}	U_{33}	U_{12}	U_{13}	U_{23}
O(1)	28.3(14)	21.1(14)	34.0(15)	1.6(11)	15.1(12)	-1.0(12)
O(2)	48.2(19)	53(2)	22.8(15)	-16.0(16)	16.6(14)	-0.6(14)
N(1)	23.5(16)	19.2(15)	18.7(15)	-0.7(13)	8.0(12)	1.8(12)
N(2)	20.7(15)	20.7(16)	19.8(15)	2.7(13)	9.6(12)	0.9(13)
N(3)	18.9(15)	30.8(18)	25.6(17)	0.3(13)	12.5(13)	2.2(14)
C(13)	20.3(17)	18.8(18)	21.6(18)	3.6(15)	11.7(14)	-2.8(15)
C(14)	25.9(19)	23.9(19)	20.7(18)	3.9(16)	11.4(15)	5.5(15)
C(7)	19.4(18)	20.1(19)	27.1(19)	5.5(15)	3.9(15)	4.7(16)
C(16)	20.1(19)	32(2)	31(2)	-7.3(16)	10.1(16)	-8.9(18)
C(6)	20.8(18)	16.9(18)	24.7(19)	8.2(14)	4.6(15)	4.8(15)
C(12)	18.5(17)	21.4(19)	19.9(18)	5.1(15)	3.2(14)	-0.6(15)
C(1)	17.8(17)	15.4(17)	26.1(19)	5.4(14)	6.9(14)	5.3(15)
C(5)	21.9(19)	16.4(18)	34(2)	-2.2(15)	4.2(16)	0.7(16)
C(8)	25.0(19)	21.0(19)	31(2)	5.0(16)	1.5(16)	-2.2(16)
C(15)	30(2)	20.7(19)	33(2)	-5.6(16)	14.5(17)	-2.1(17)
C(2)	19.2(17)	21.1(18)	22.7(19)	4.0(15)	5.7(14)	3.3(15)
C(17)	21.2(18)	30(2)	19.4(18)	3.1(16)	9.7(15)	1.0(16)
C(4)	18.8(18)	23(2)	41(2)	-1.1(16)	8.8(16)	10.6(18)
C(3)	23.9(19)	29(2)	28(2)	4.8(16)	13.2(16)	7.0(17)
C(11)	20.7(18)	28(2)	23.1(19)	1.8(16)	6.4(15)	1.1(16)
C(9)	31(2)	35(2)	24(2)	8.6(18)	5.6(17)	-7.9(17)
C(10)	23(2)	40(2)	28(2)	9.1(18)	12.9(17)	2.8(18)

Table S12. Bond lengths for pg1_120k_rep_0ma.

Atom	Atom	Length/Å	Atom	Atom	Length/Å
O(1)	N(3)	1.227(4)	C(16)	C(15)	1.379(6)
O(2)	N(3)	1.228(4)	C(16)	C(17)	1.384(6)

Table S12. Bond lengths for pg1_120k_rep_0ma.

Atom	Atom	Length/Å	Atom	Atom	Length/Å
N(1)	C(13)	1.424(5)	C(6)	C(1)	1.403(5)
N(1)	C(12)	1.411(5)	C(6)	C(5)	1.399(5)
N(1)	C(1)	1.393(5)	C(12)	C(11)	1.395(5)
N(2)	C(13)	1.328(5)	C(1)	C(2)	1.410(5)
N(2)	C(17)	1.340(5)	C(5)	C(4)	1.377(6)
N(3)	C(2)	1.452(5)	C(8)	C(9)	1.380(6)
C(13)	C(14)	1.393(5)	C(2)	C(3)	1.384(5)
C(14)	C(15)	1.372(5)	C(4)	C(3)	1.381(6)
C(7)	C(6)	1.444(5)	C(11)	C(10)	1.384(6)
C(7)	C(12)	1.399(5)	C(9)	C(10)	1.398(6)
C(7)	C(8)	1.393(5)			

Table S13 Bond angles for pg1_120k_rep_0ma.

Atom	Atom	Atom	Angle/°	Atom	Atom	Atom	Angle/°
C(12)	N(1)	C(13)	123.4(3)	C(7)	C(12)	N(1)	109.1(3)
C(1)	N(1)	C(13)	125.9(3)	C(11)	C(12)	N(1)	129.1(3)
C(1)	N(1)	C(12)	107.5(3)	C(11)	C(12)	C(7)	121.8(3)
C(17)	N(2)	C(13)	116.8(3)	C(6)	C(1)	N(1)	109.4(3)
O(2)	N(3)	O(1)	123.6(3)	C(2)	C(1)	N(1)	132.5(3)
C(2)	N(3)	O(1)	118.2(3)	C(2)	C(1)	C(6)	118.1(3)
C(2)	N(3)	O(2)	118.1(3)	C(4)	C(5)	C(6)	119.0(4)
N(2)	C(13)	N(1)	115.6(3)	C(9)	C(8)	C(7)	118.8(4)
C(14)	C(13)	N(1)	120.3(3)	C(16)	C(15)	C(14)	119.7(4)
C(14)	C(13)	N(2)	124.2(3)	C(1)	C(2)	N(3)	122.8(3)
C(15)	C(14)	C(13)	117.6(4)	C(3)	C(2)	N(3)	117.1(3)
C(12)	C(7)	C(6)	106.9(3)	C(3)	C(2)	C(1)	119.7(4)
C(8)	C(7)	C(6)	133.1(4)	C(16)	C(17)	N(2)	123.3(4)
C(8)	C(7)	C(12)	120.0(4)	C(3)	C(4)	C(5)	120.6(4)
C(17)	C(16)	C(15)	118.4(4)	C(4)	C(3)	C(2)	121.1(4)

Table S13 Bond angles for pg1_120k_rep_0ma.

Atom	Atom	Atom	Angle/°	Atom	Atom	Atom	Angle/°
C(1)	C(6)	C(7)	107.1(3)	C(10)	C(11)	C(12)	116.9(4)
C(5)	C(6)	C(7)	131.5(4)	C(10)	C(9)	C(8)	120.4(4)
C(5)	C(6)	C(1)	121.4(3)	C(9)	C(10)	C(11)	122.0(4)

Table S14. Torsion angles for pg1_120k_rep_0ma.

A	B	C	D	Angle/°	A	B	C	D	Angle/°
O(1)	N(3)	C(2)	C(1)	-31.0(4)	N(3)	C(2)	C(1)	C(6)	171.3(3)
O(1)	N(3)	C(2)	C(3)	142.5(3)	N(3)	C(2)	C(3)	C(4)	-170.1(3)
O(2)	N(3)	C(2)	C(1)	151.1(3)	C(13)	C(14)	C(15)	C(16)	1.0(4)
O(2)	N(3)	C(2)	C(3)	-35.4(4)	C(14)	C(15)	C(16)	C(17)	-0.7(4)
N(1)	C(13)	N(2)	C(17)	178.4(3)	C(7)	C(6)	C(1)	C(2)	-179.1(3)
N(1)	C(13)	C(14)	C(15)	-179.8(3)	C(7)	C(6)	C(5)	C(4)	179.7(4)
N(1)	C(12)	C(7)	C(6)	1.1(3)	C(7)	C(12)	C(11)	C(10)	-0.3(4)
N(1)	C(12)	C(7)	C(8)	179.6(3)	C(7)	C(8)	C(9)	C(10)	-0.3(4)
N(1)	C(12)	C(11)	C(10)	-179.5(4)	C(6)	C(1)	C(2)	C(3)	-2.1(4)
N(1)	C(1)	C(6)	C(7)	-0.6(3)	C(6)	C(5)	C(4)	C(3)	-0.1(4)
N(1)	C(1)	C(6)	C(5)	178.0(3)	C(12)	C(11)	C(10)	C(9)	0.1(4)
N(1)	C(1)	C(2)	N(3)	-6.8(5)	C(1)	C(2)	C(3)	C(4)	3.6(4)
N(1)	C(1)	C(2)	C(3)	179.9(4)	C(5)	C(4)	C(3)	C(2)	-2.5(5)
N(2)	C(13)	C(14)	C(15)	-0.0(4)	C(8)	C(9)	C(10)	C(11)	0.2(5)
N(2)	C(17)	C(16)	C(15)	-0.8(4)					

Table S15. Hydrogen atom coordinates (Å×10^4) and isotropic displacement parameters (Å^2×10^3) for pg1_120k_rep_0ma.

Atom	*x*	*y*	*z*	U(eq)
H(14)	6313(4)	2500(3)	3888(4)	7(8)
H(16)	3470(4)	2349(3)	599(4)	26(11)
H(5)	10244(4)	6921(3)	4335(4)	14(9)

Table S15. Hydrogen atom coordinates (Å×10^4) and isotropic displacement parameters (Å^2×10^3) for pg1_120k_rep_0ma.

**Atom**	***x***	***y***	***z***	**U(eq)**
H(8)	9163(4)	6740(3)	6379(4)	21(10)
H(15)	4543(4)	1573(3)	2581(4)	28(11)
H(17)	4171(3)	4037(3)	-3(4)	14(9)
H(4)	10784(4)	6807(3)	2385(4)	32(12)
H(3)	9679(4)	5620(3)	783(4)	25(11)
H(11)	5938(4)	3992(3)	5265(4)	21(10)
H(9)	7956(4)	6260(4)	7804(4)	27(11)
H(10)	6363(4)	4910(4)	7245(4)	14(9)

pg1_120k_rep_0ma

10. References

1. J. Dharaniyedath, V. Kumar, P. Gandeepan, *Eur. J. Org. Chem.* **2024**, *27*, e202400649. doi: 10.1002/ejoc.202400649.
2. J. A. Leitch, C. J. Heron, J. McKnight, G. Kociok-Köhn, Y. Bhonoah, C. G. Frost, *Chem. Commun.* **2017**, *53*, 13039-13042. doi: 10.1039/C7CC07606A.
3. F. Zhao, Z. Zhou, Y. Lu, J. Qiao, X. Zhang, X. Gong, S. Liu, S. Lin, X. Wu, W. Yi, *ACS Catal.* **2021**, *11*, 13921-13934. doi:10.1021/acscatal.1c03846.
4. L. Przypis, K. Z. Walczak, *J. Org. Chem.* **2019**, *84*, 2287-2296. doi: 10.1021/acs.joc.8b02821.
5. C. D. Campbell, M. I. Stewart, *J. Chem. Educ.* **2023**, *100*, 3171-3178. doi: 10.1021/acs.jchemed.3c00283.
6. P. Dinér, G. Proietti, K. J. Prathap, X. Ye, R. T. Olsson, *Synthesis* **2021**, *54*, 133-146. doi: 10.1055/a-1579-2190.
7. K. Kubota, A. Nagao, H. Ito, *RSC Mechanochem.* **2025**, *2*, 389-393. doi: 10.1039/D4MR00138A.
8. G. M. Reddy, N. S. Rao, M. Hariharasarma, *Asian J. Org. Chem.* **2016**, *6*, 59-62. doi: 10.1002/ajoc.201600422.
9. I. Alimi, R. Remy, C. G. Bochet, *Eur. J. Org. Chem.* **2017**, *2017*, 3197-3210. doi: 10.1002/ejoc.201700300.
10. J. R. Hiscock, C. Caltagirone, M. E. Light, M. B. Hursthouse, P. A. Gale, *Org. Biomol. Chem.* **2009**, *7*, 1781-1783. doi: 10.1039/B900178F.
11. B. A. Dalvi, P. D. Lokhande, *Tetrahedron Lett.* **2018**, *59*, 2145-2149. doi: 10.1016/j.tetlet.2018.01.061.

11. NMR spectra

^{1}H and $^{13}C\{^{1}H\}$ NMR spectra of compound **2a**

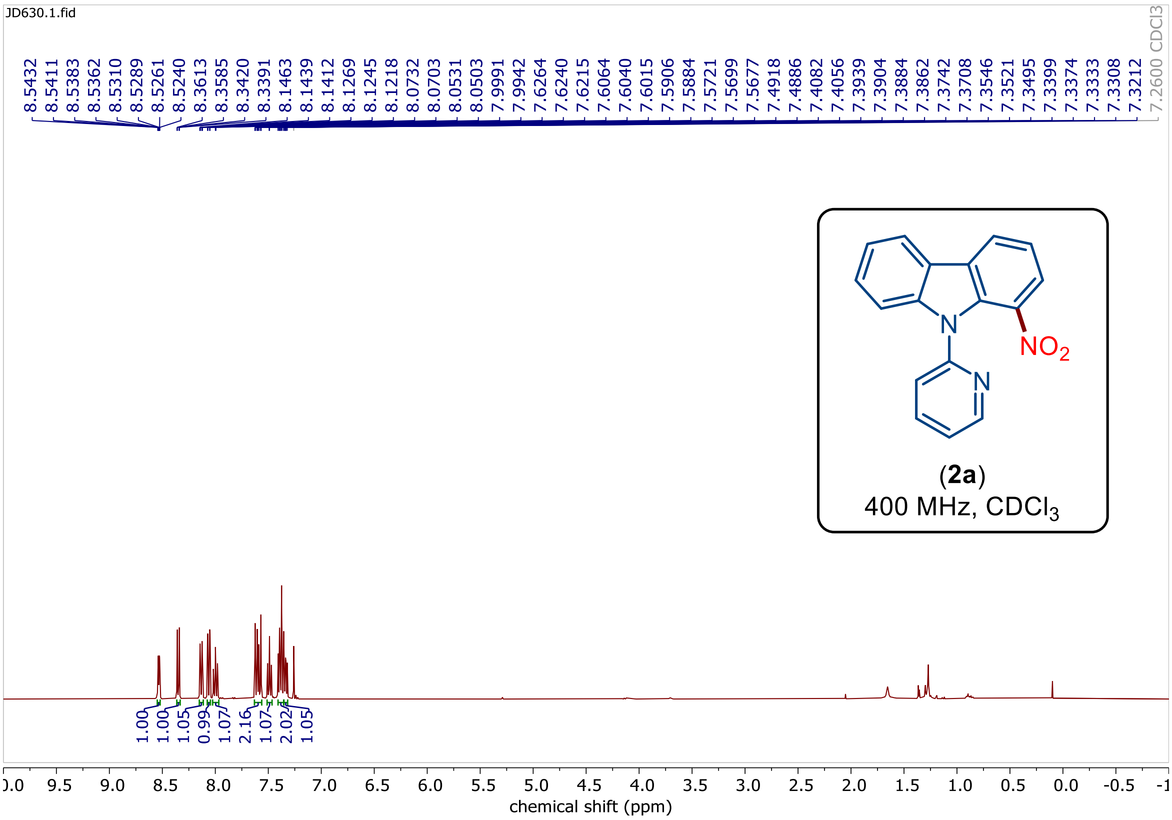

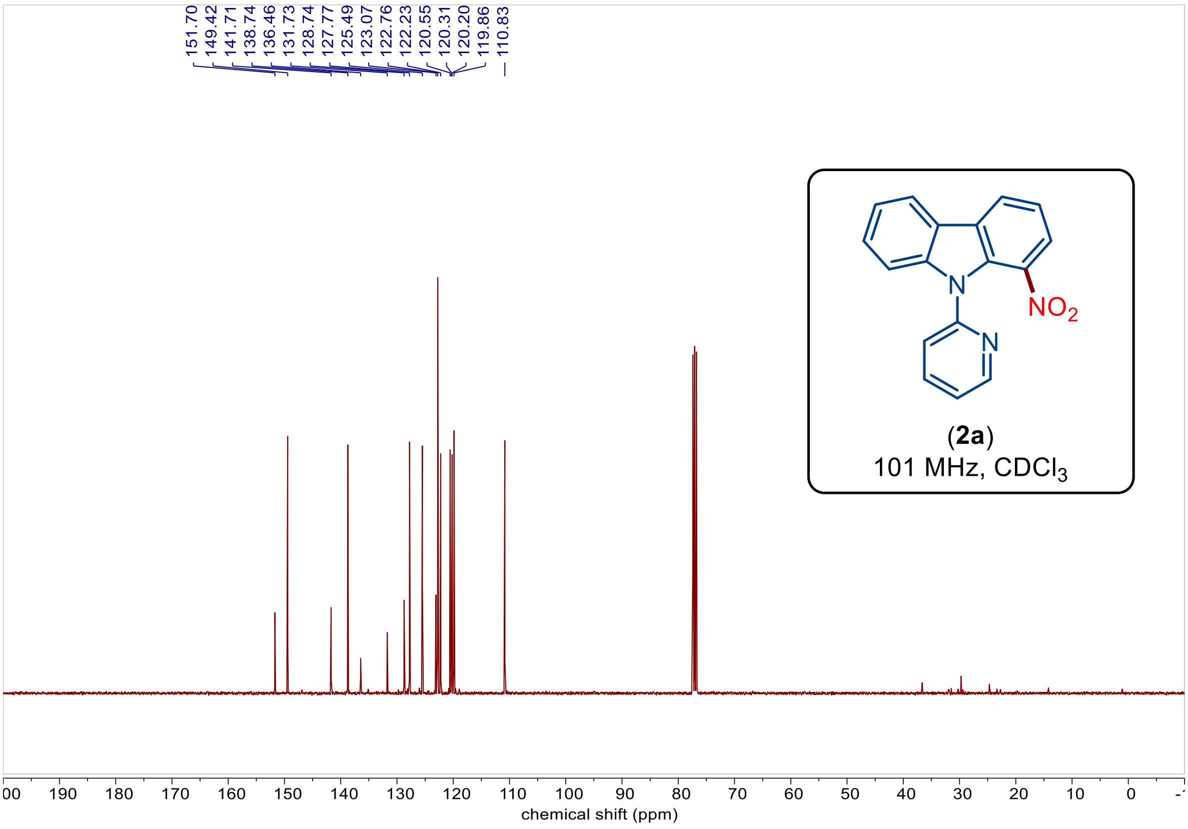

^{1}H and ^{13}C{^{1}H} NMR spectra of compound **2b**

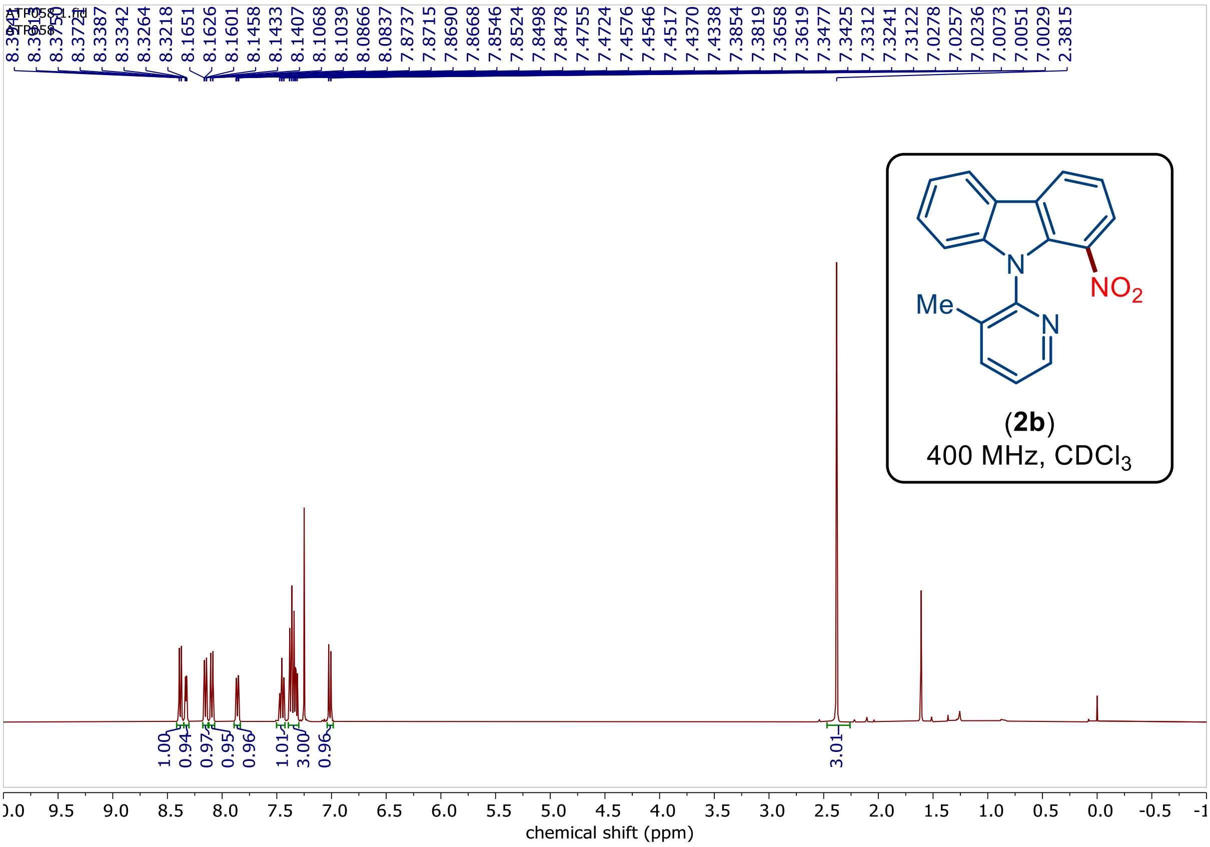

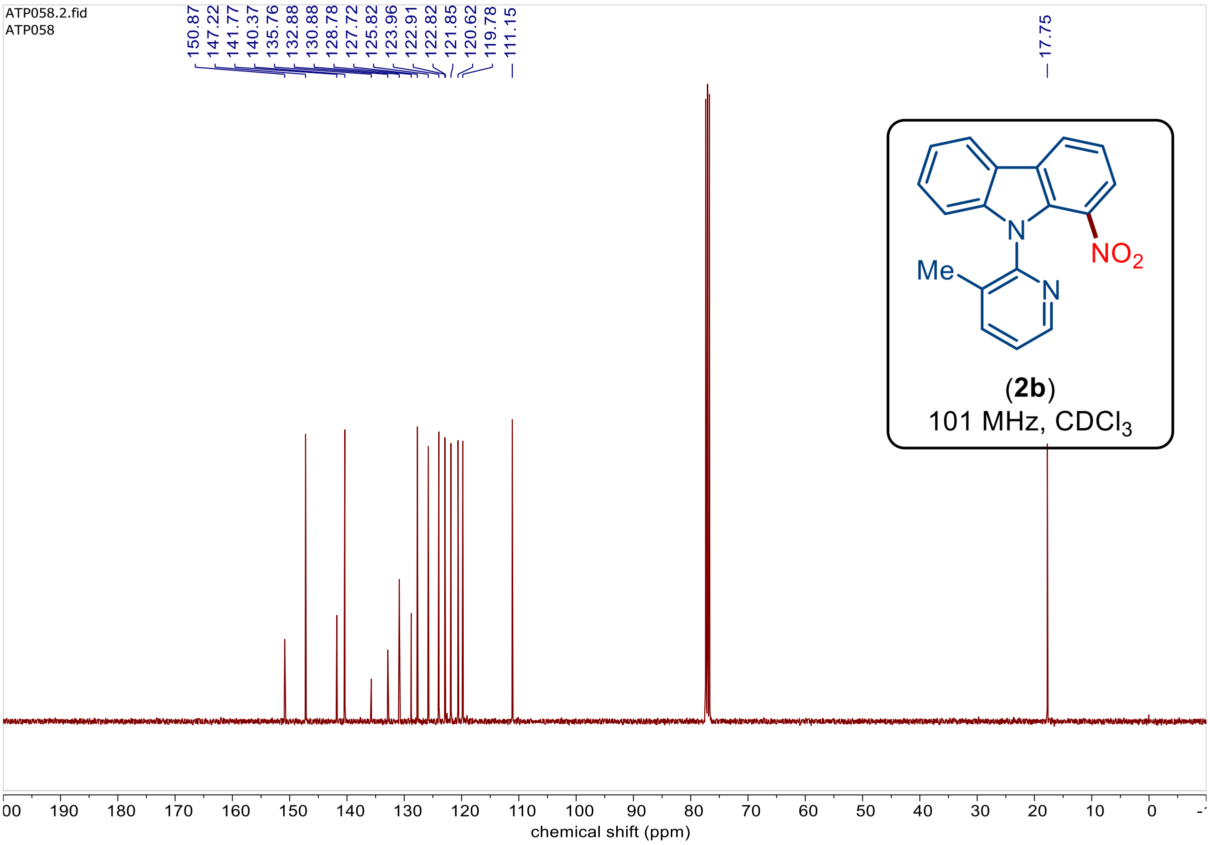

^{1}H and ^{13}C{^{1}H} NMR spectra of compound **2c**

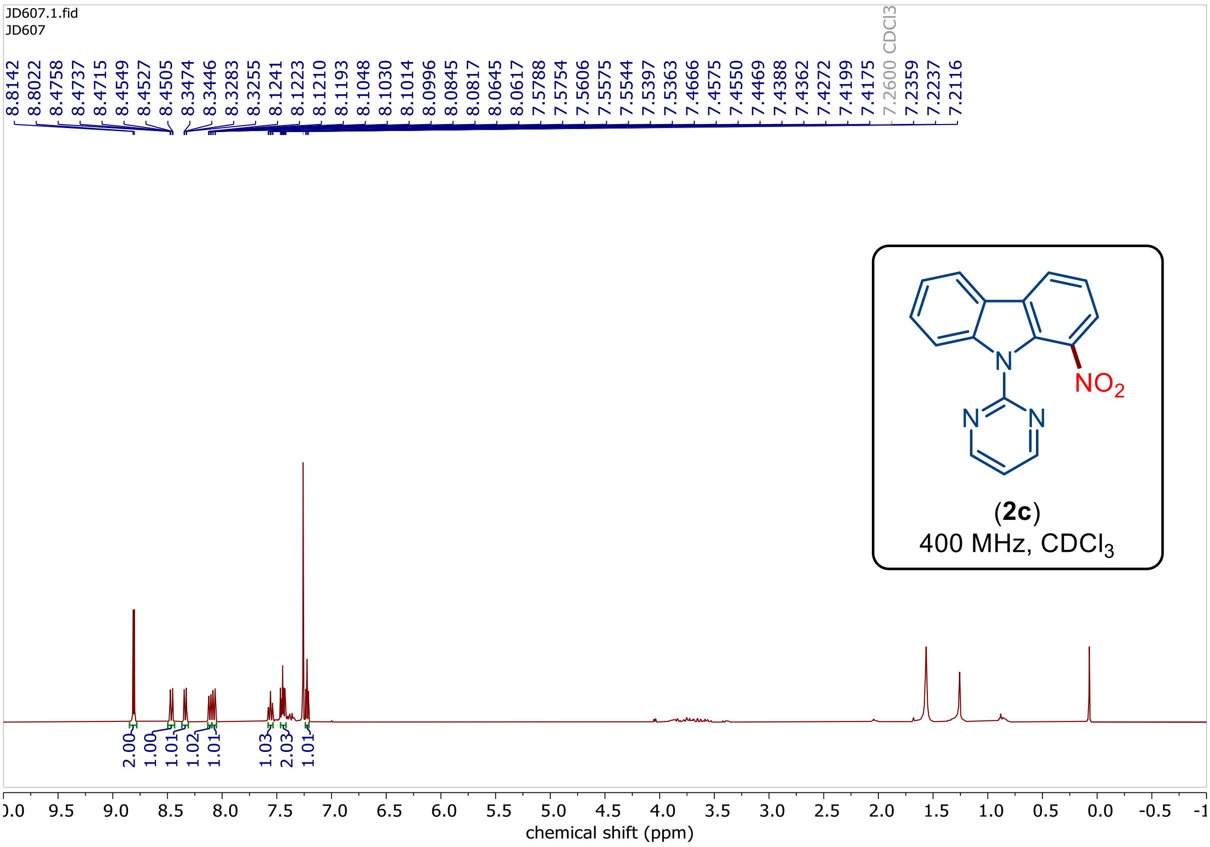

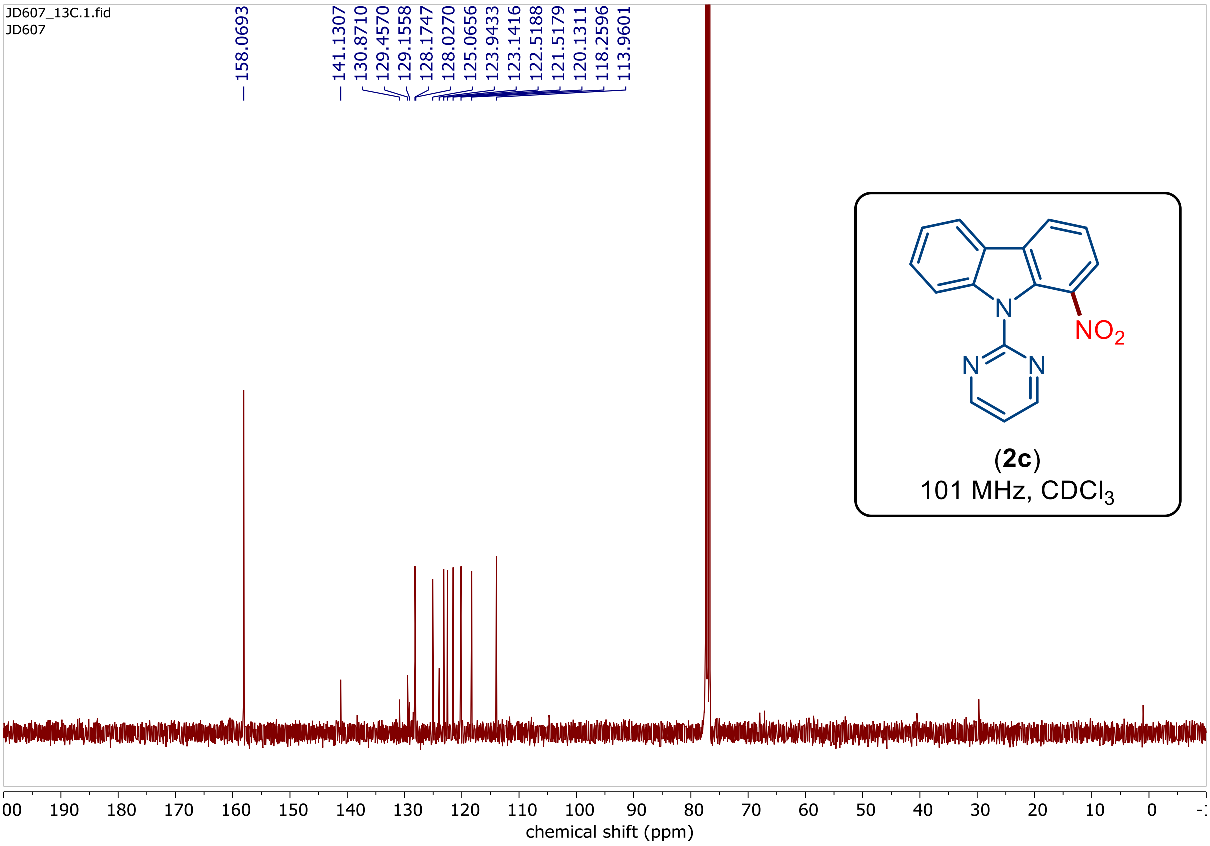

^{1}H and $^{13}C\{^{1}H\}$ NMR spectra of compound **2d**

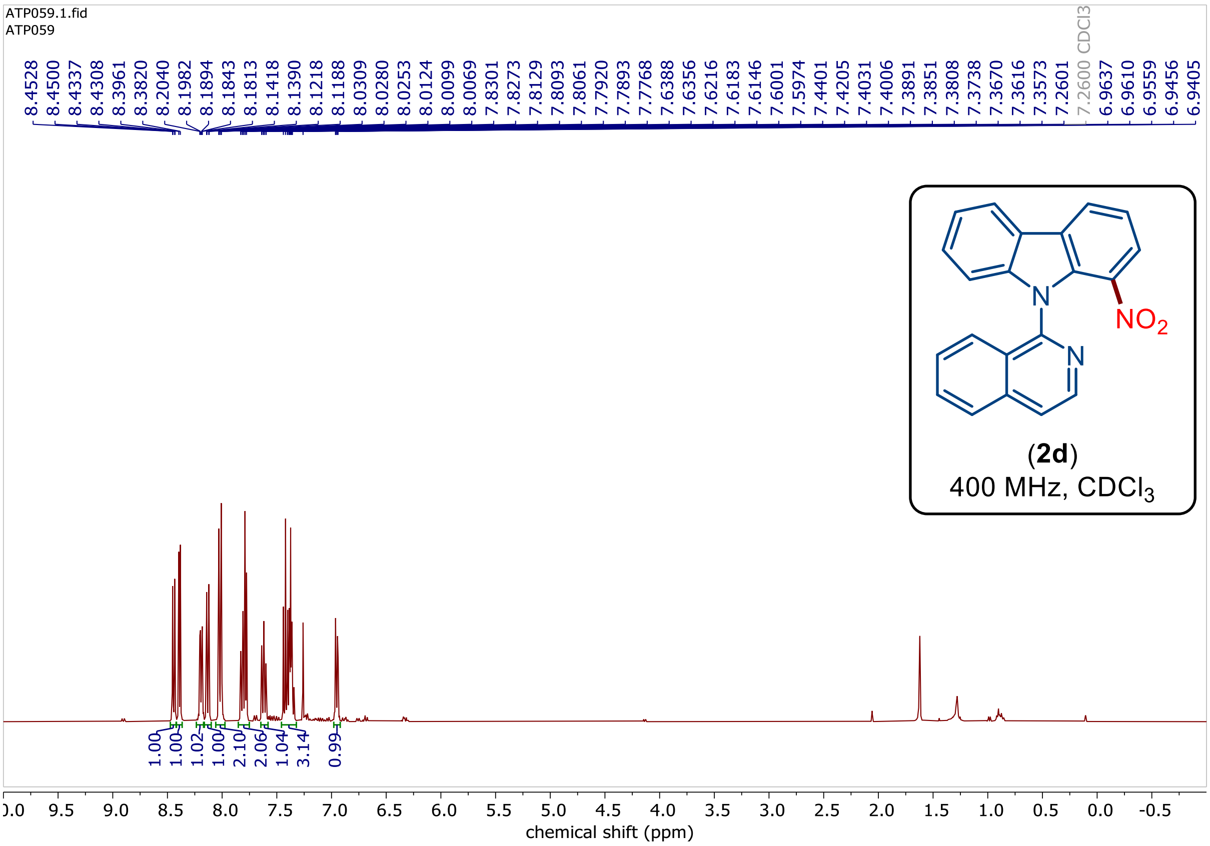

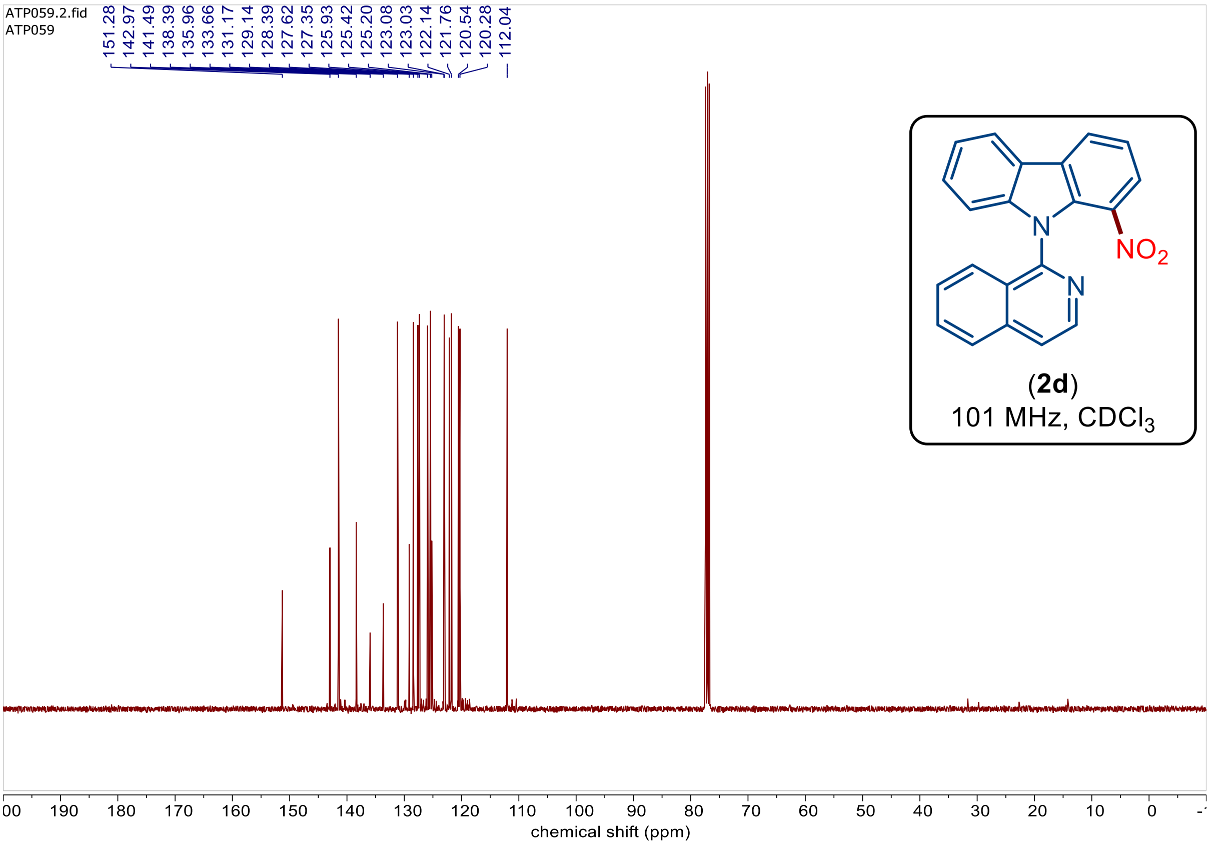

^{1}H and $^{13}C\{^{1}H\}$ NMR spectra of compound **2j**

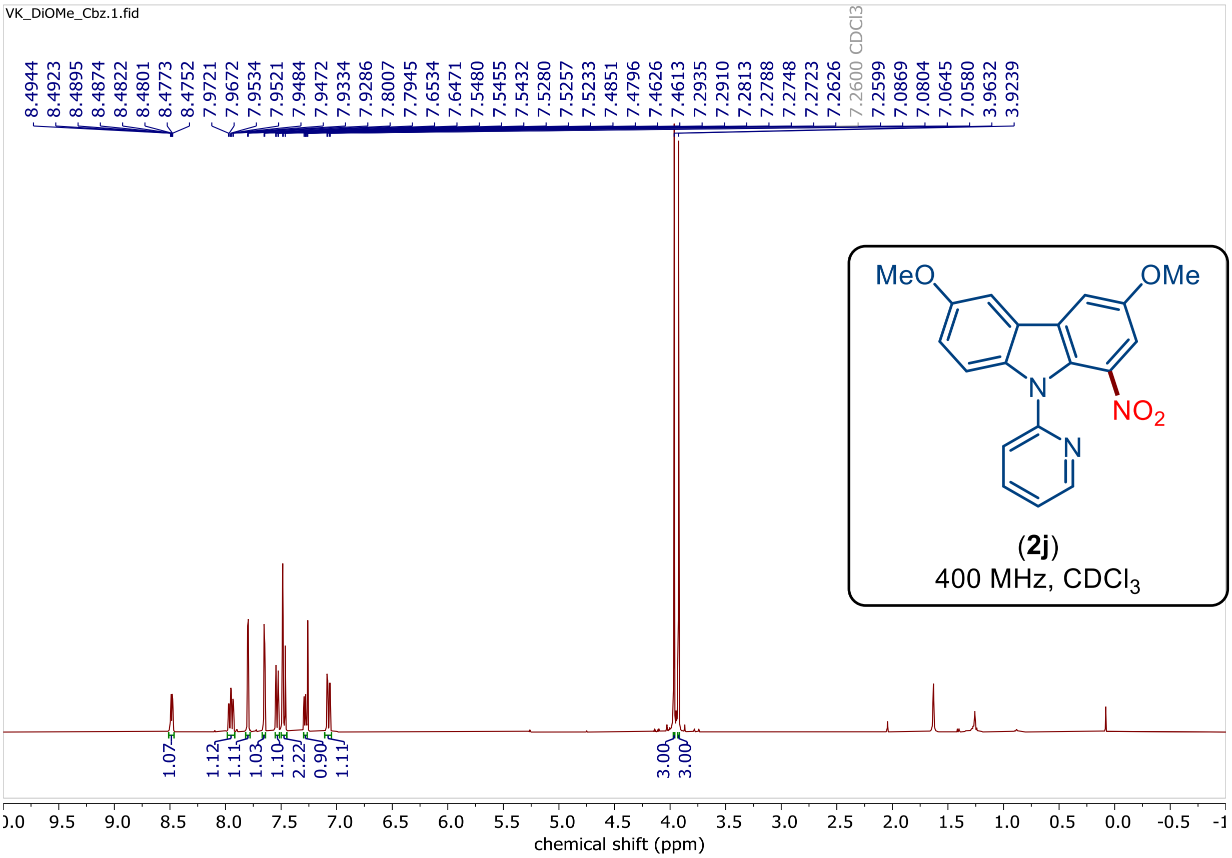

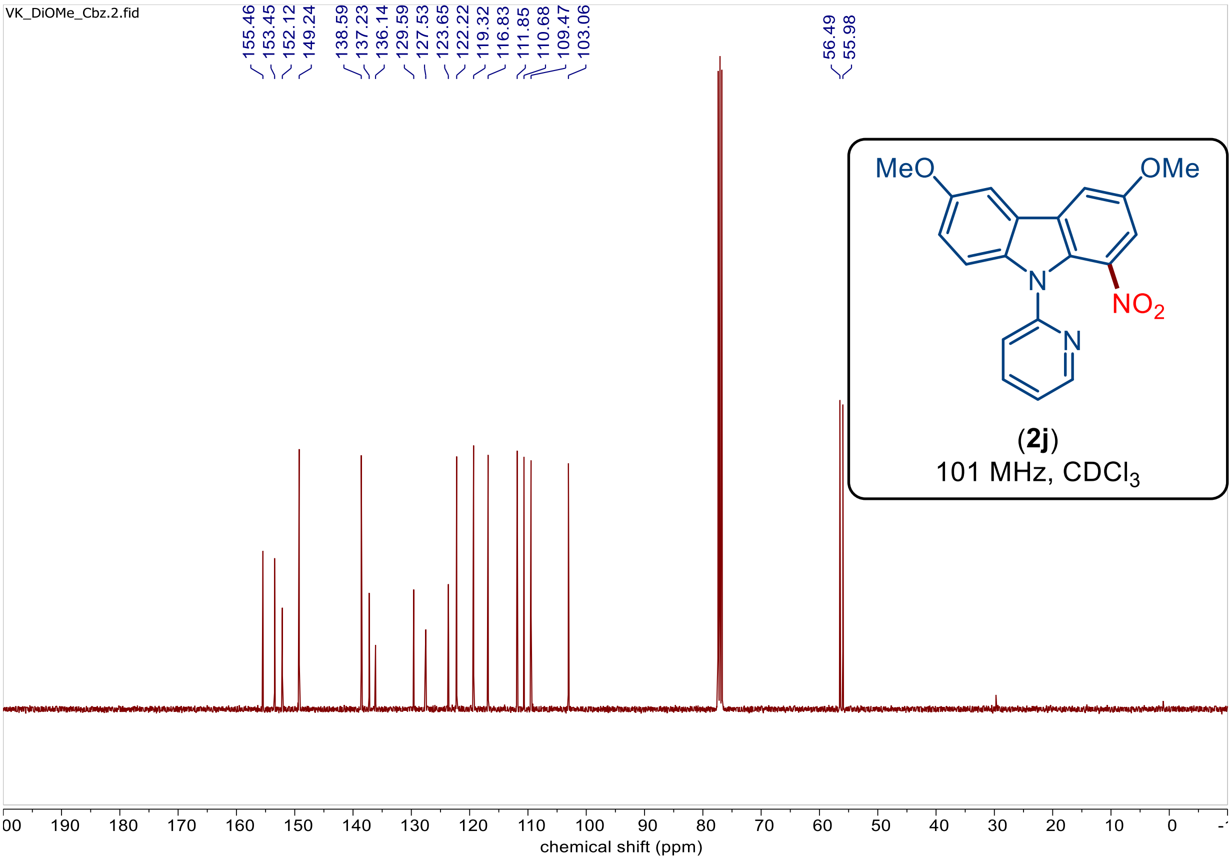

^{1}H and ^{13}C{^{1}H} NMR spectra of compound **2k**

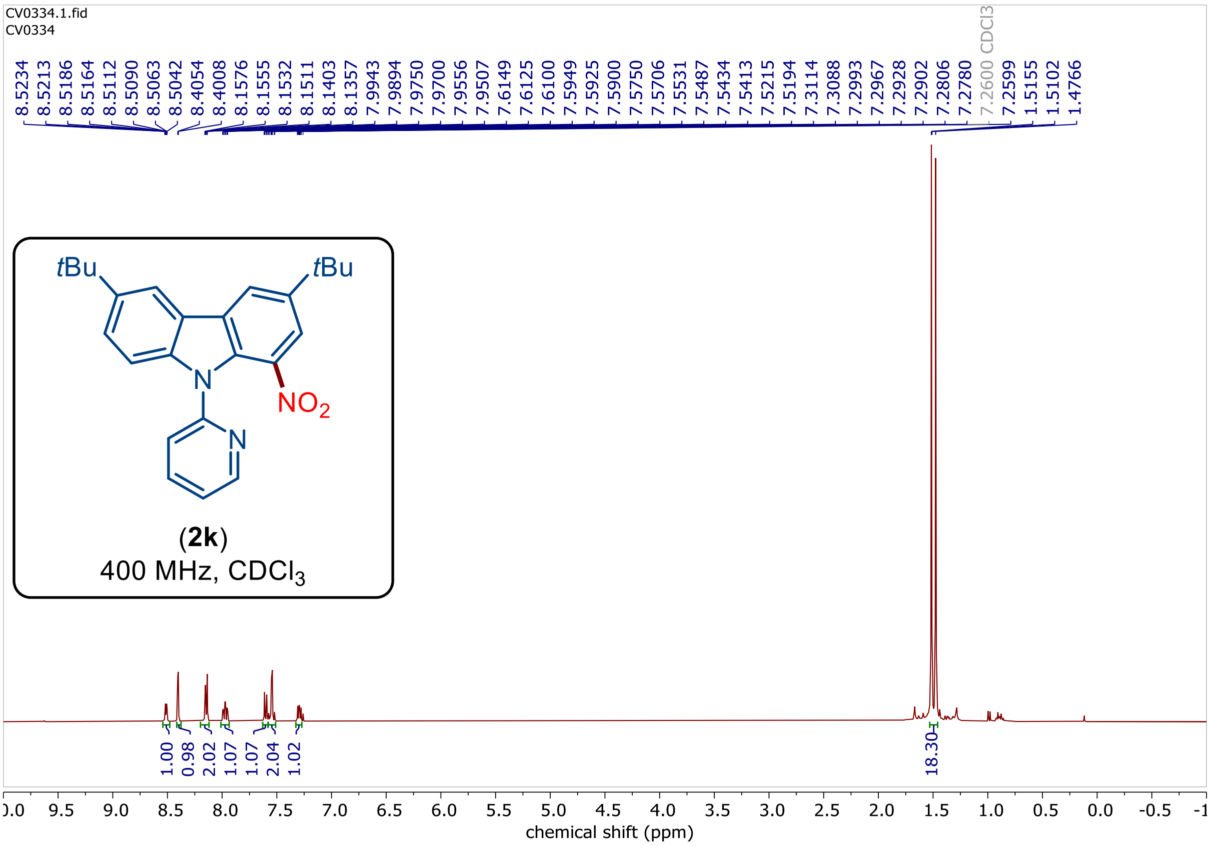

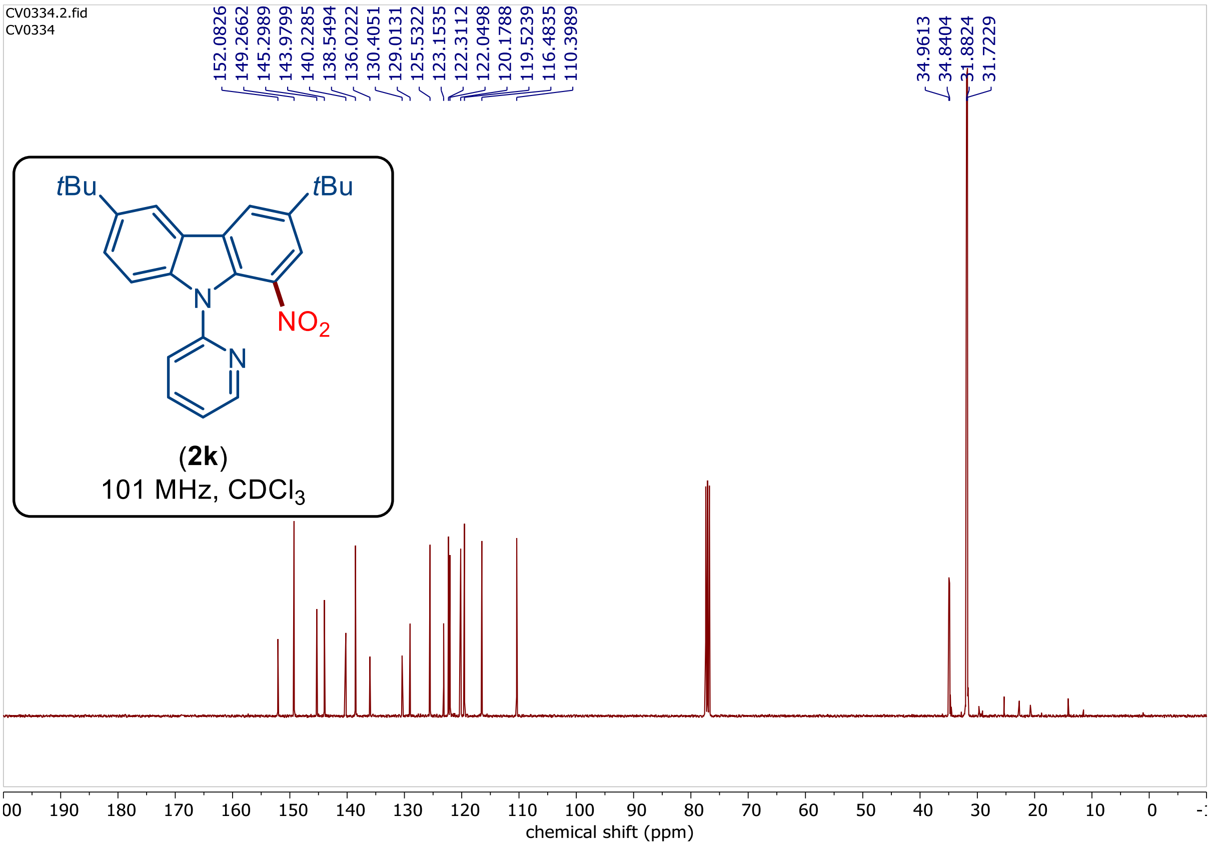

^{1}H and ^{13}C{^{1}H} NMR spectra of compound **2l**

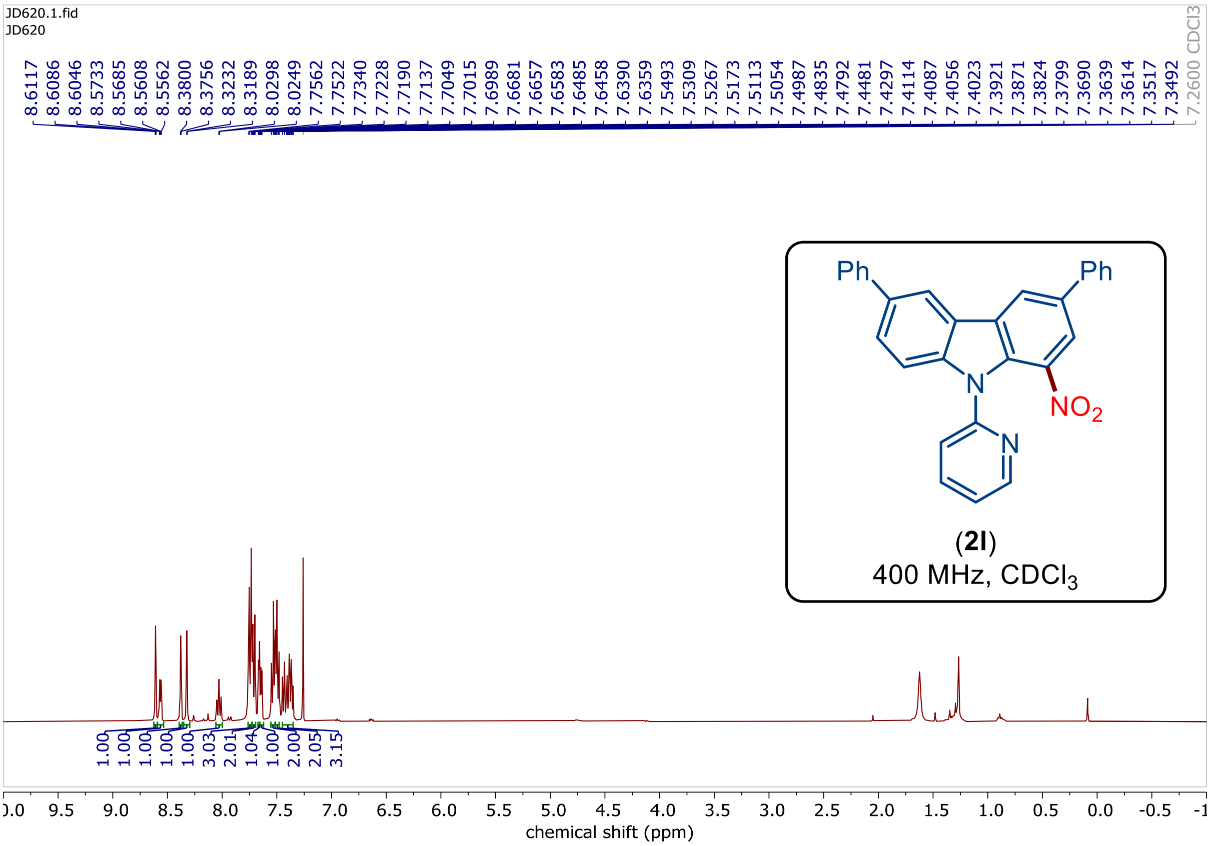

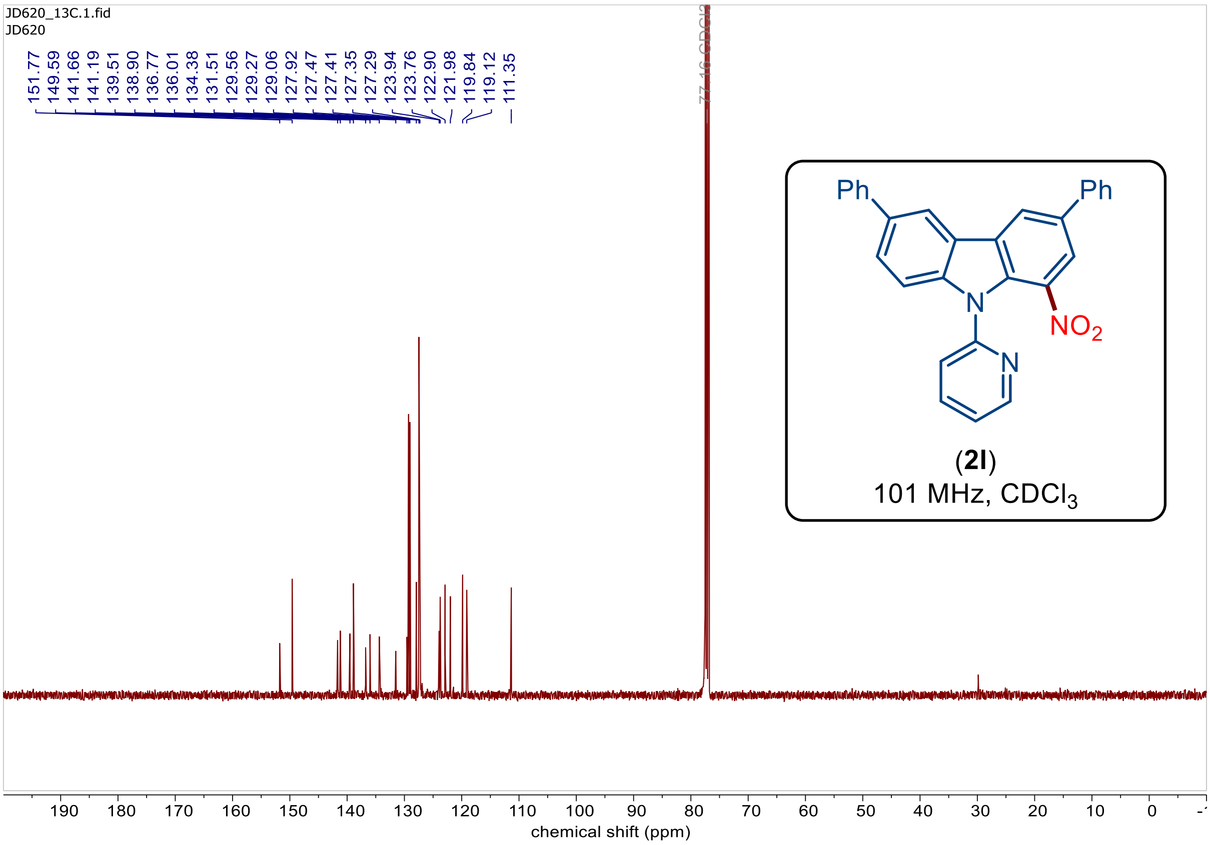

^{1}H and ^{13}C{^{1}H} NMR spectra of compound **2m**

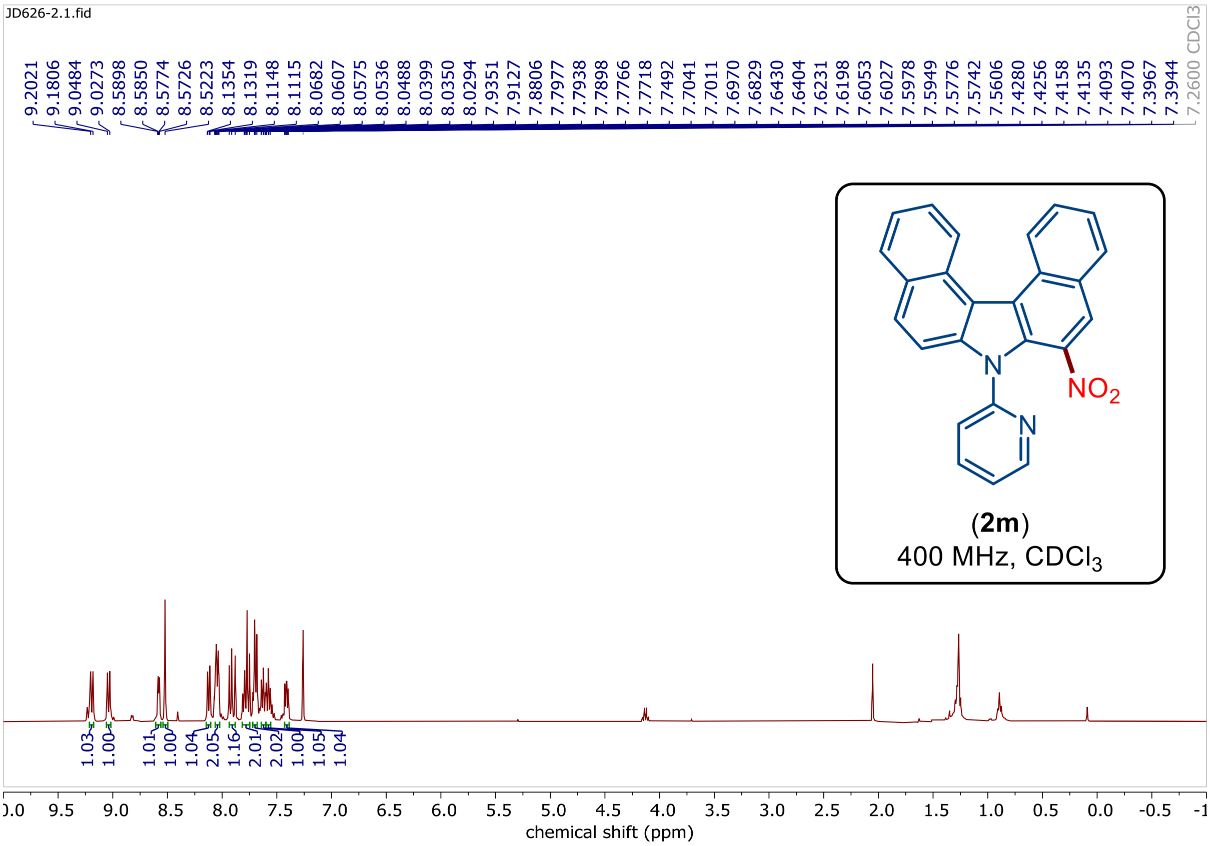

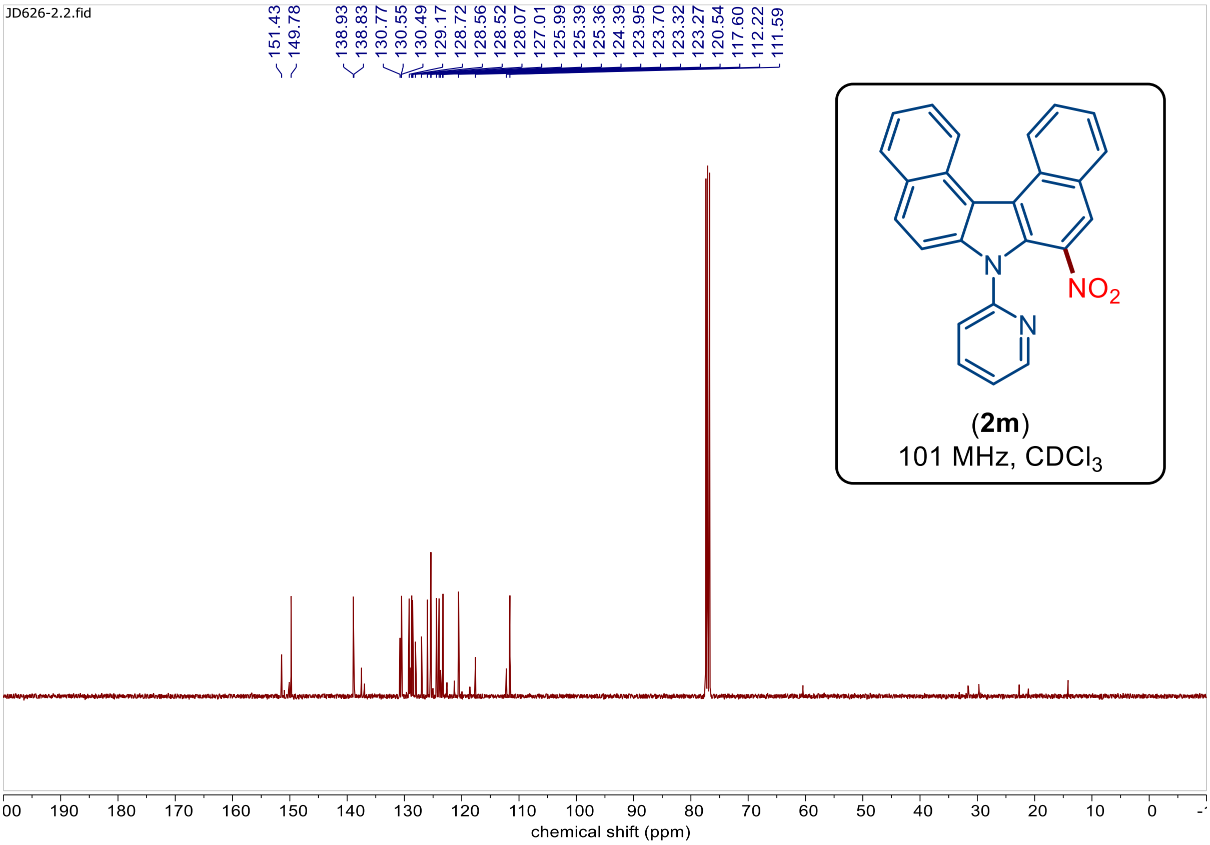

^{1}H and ^{13}C{^{1}H} NMR spectra of compound **2n**

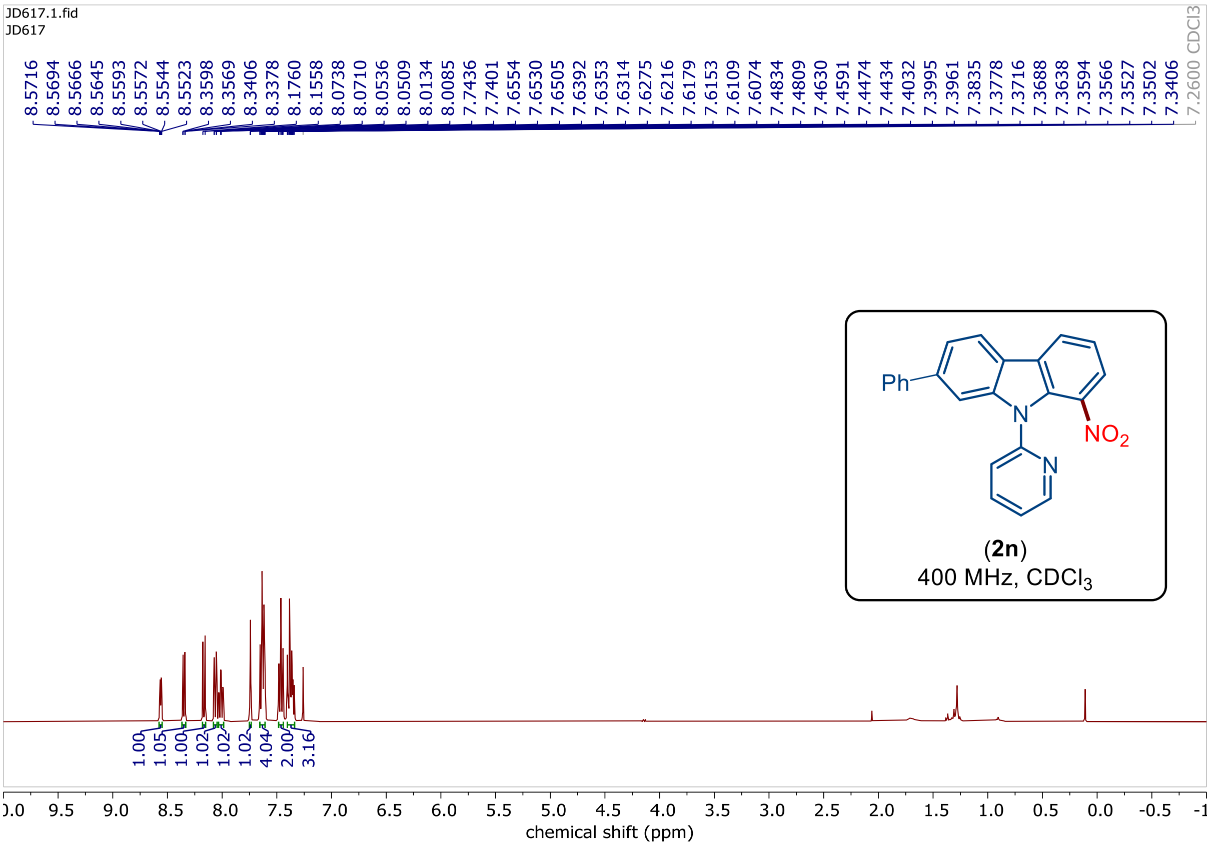

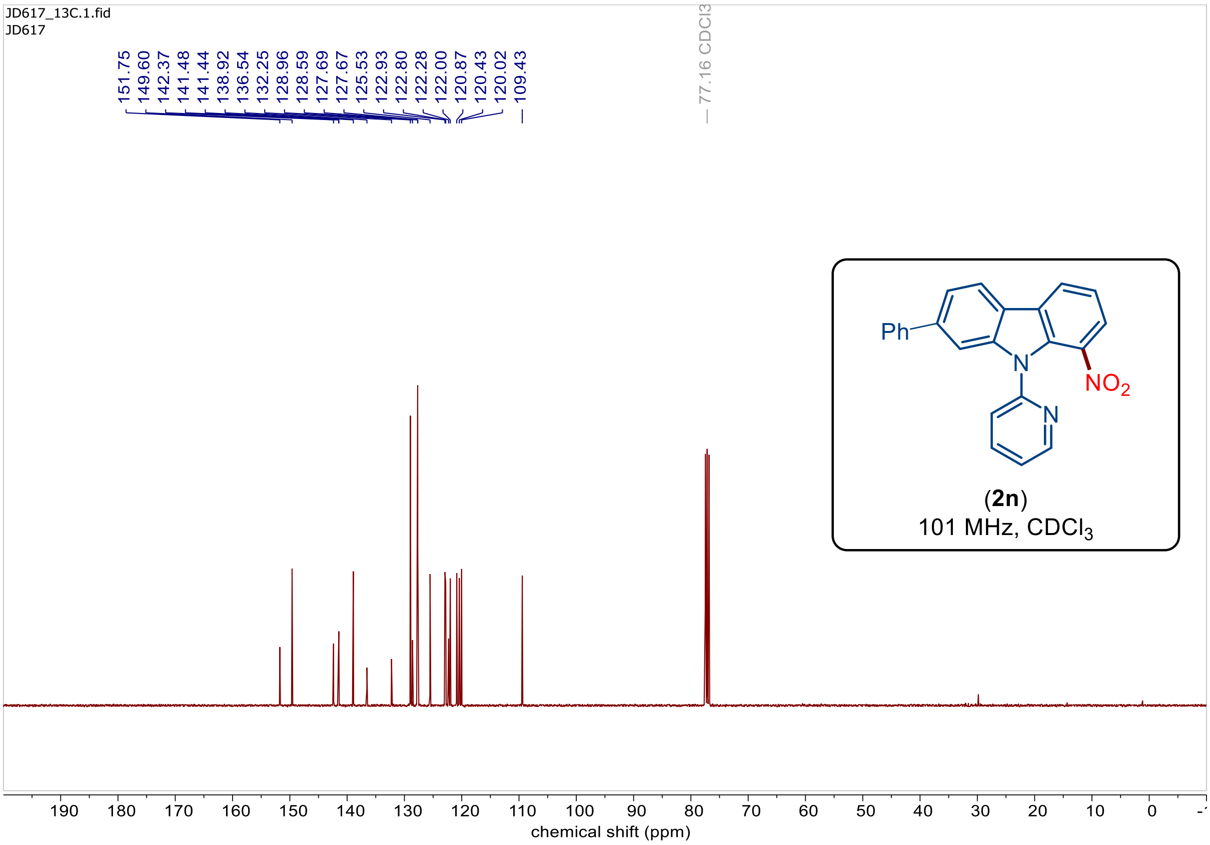

^{1}H and $^{13}C\{^{1}H\}$ NMR spectra of compound **2o**

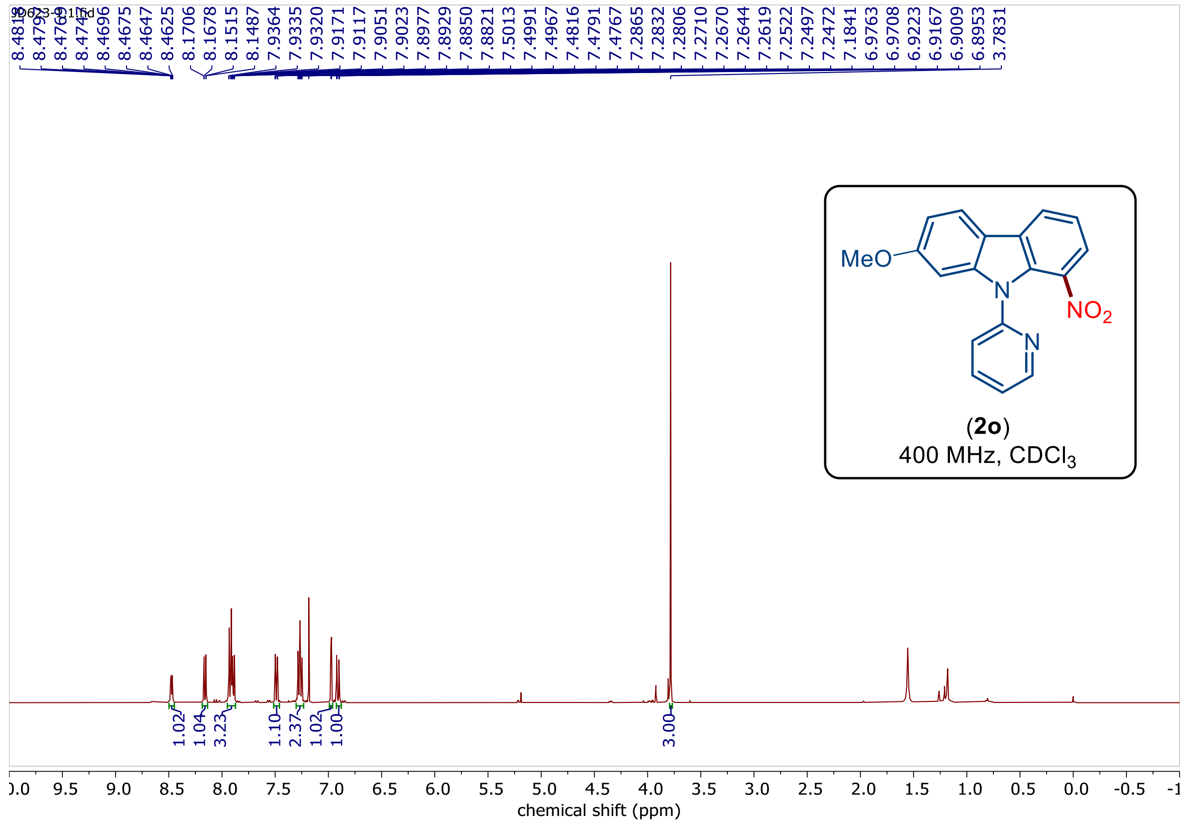

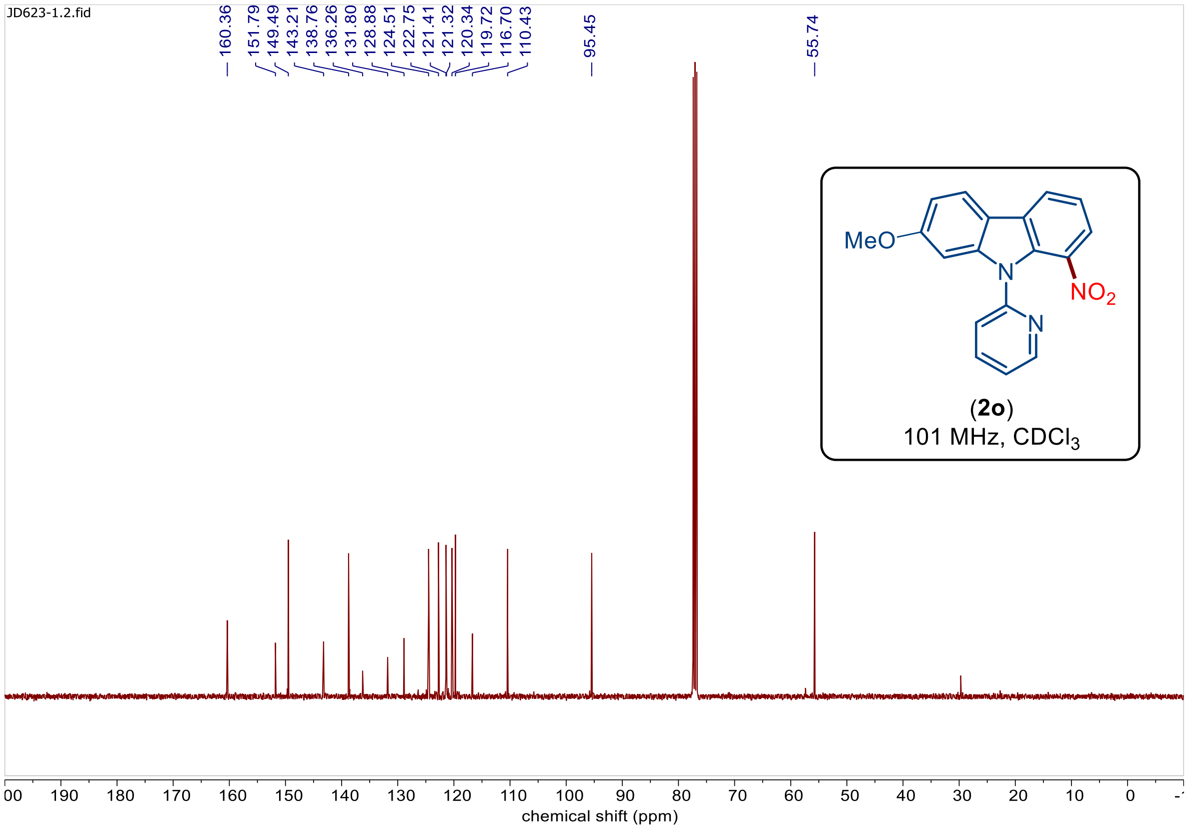

^{1}H and ^{13}C{^{1}H} NMR spectra of compound **2p**

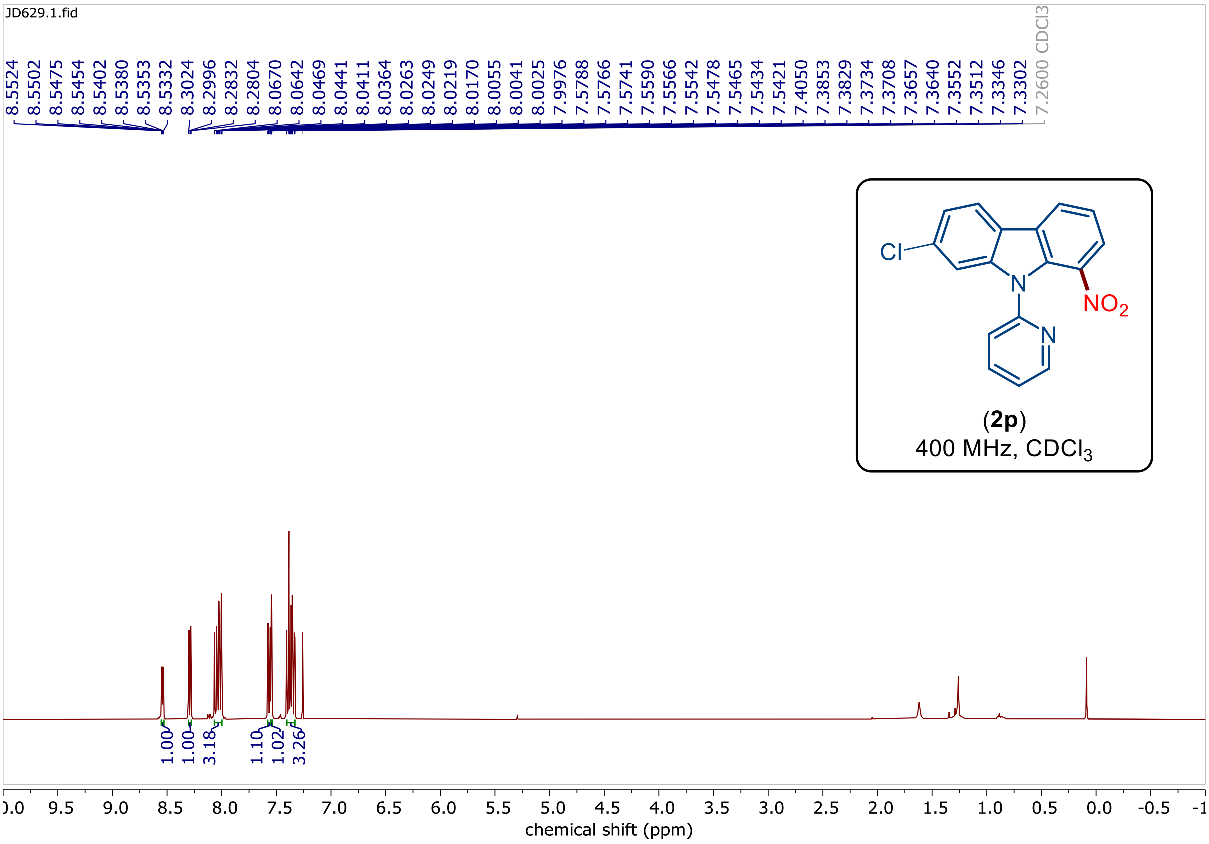

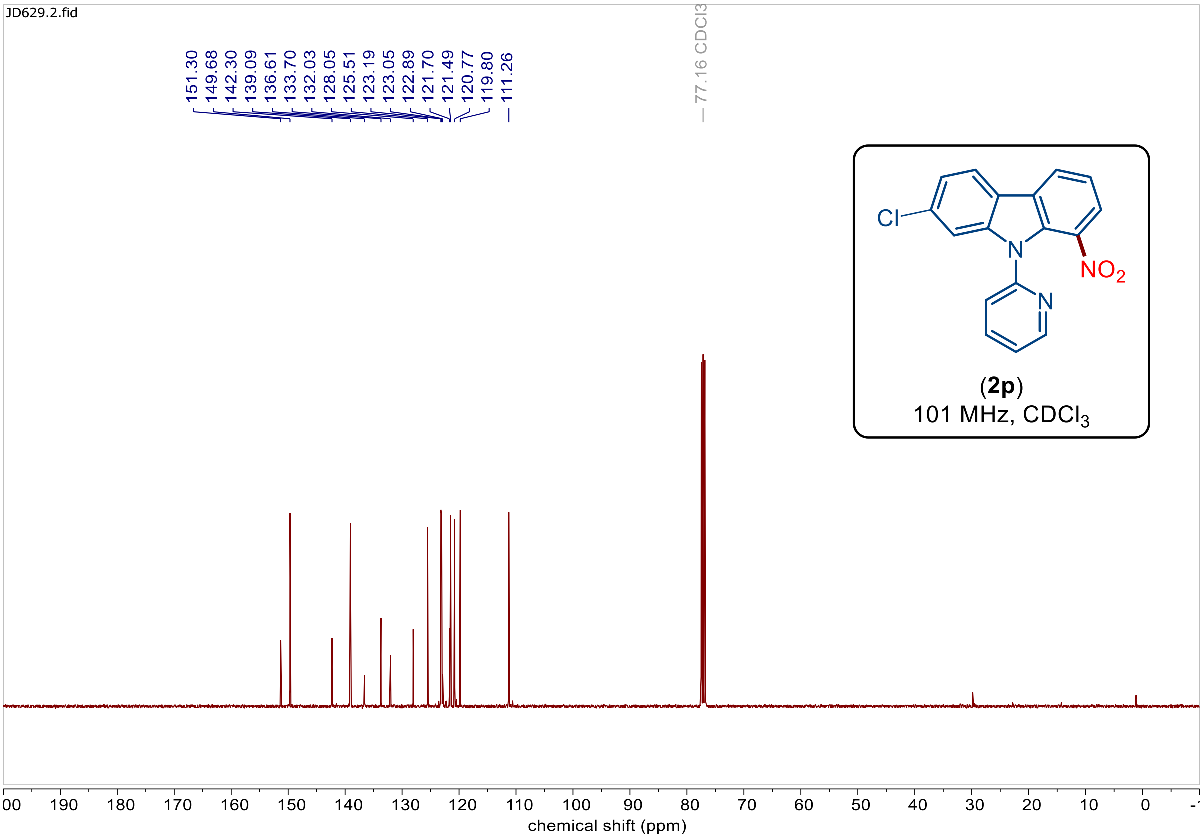

^{1}H and $^{13}C\{^{1}H\}$ NMR spectra of compound **2q**

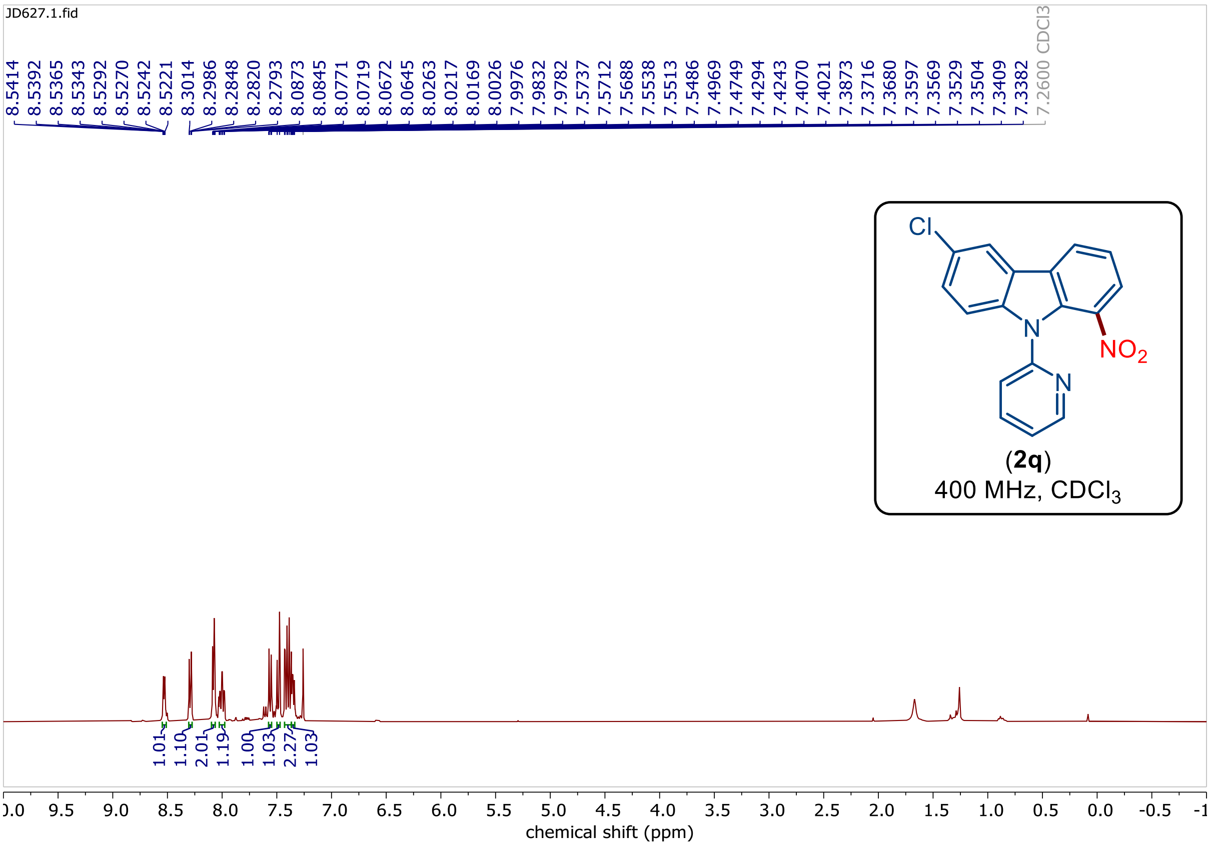

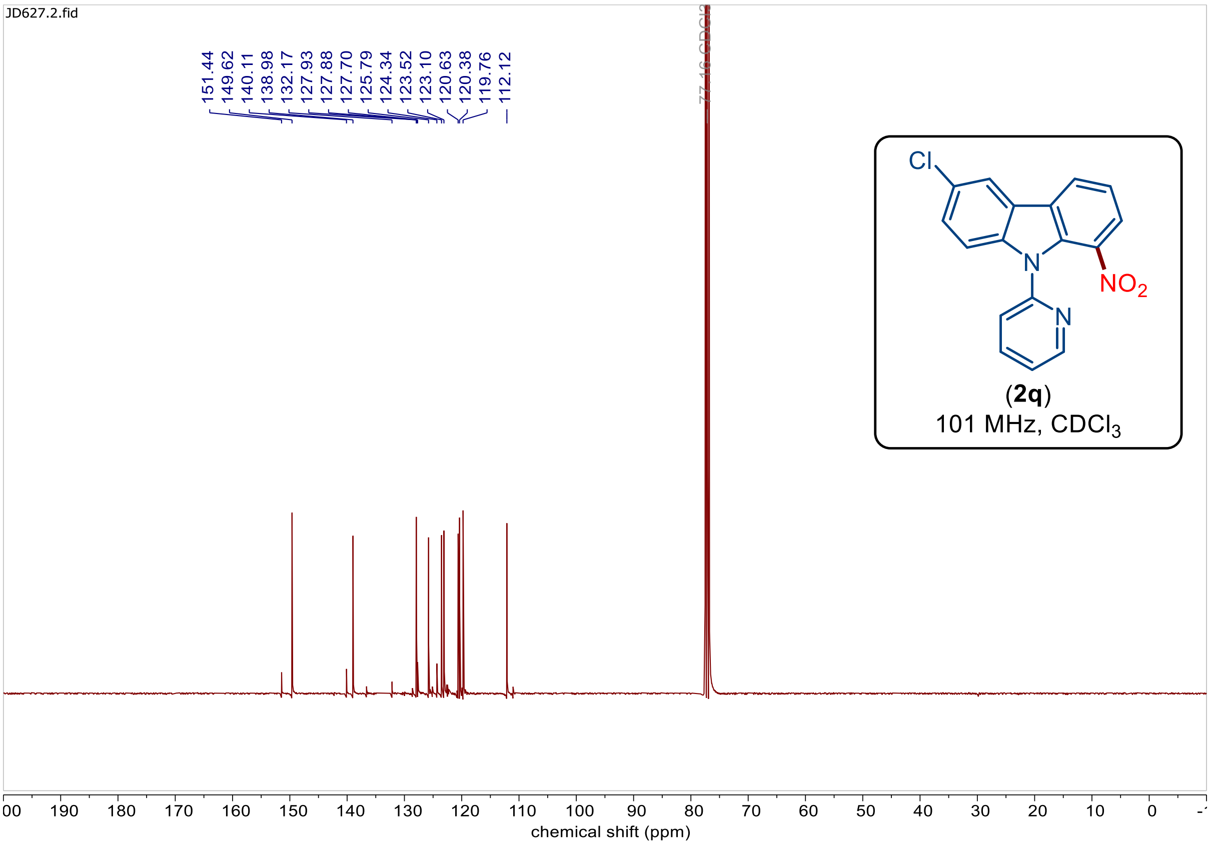

^{1}H and ^{13}C{^{1}H} NMR spectra of compound **2r**

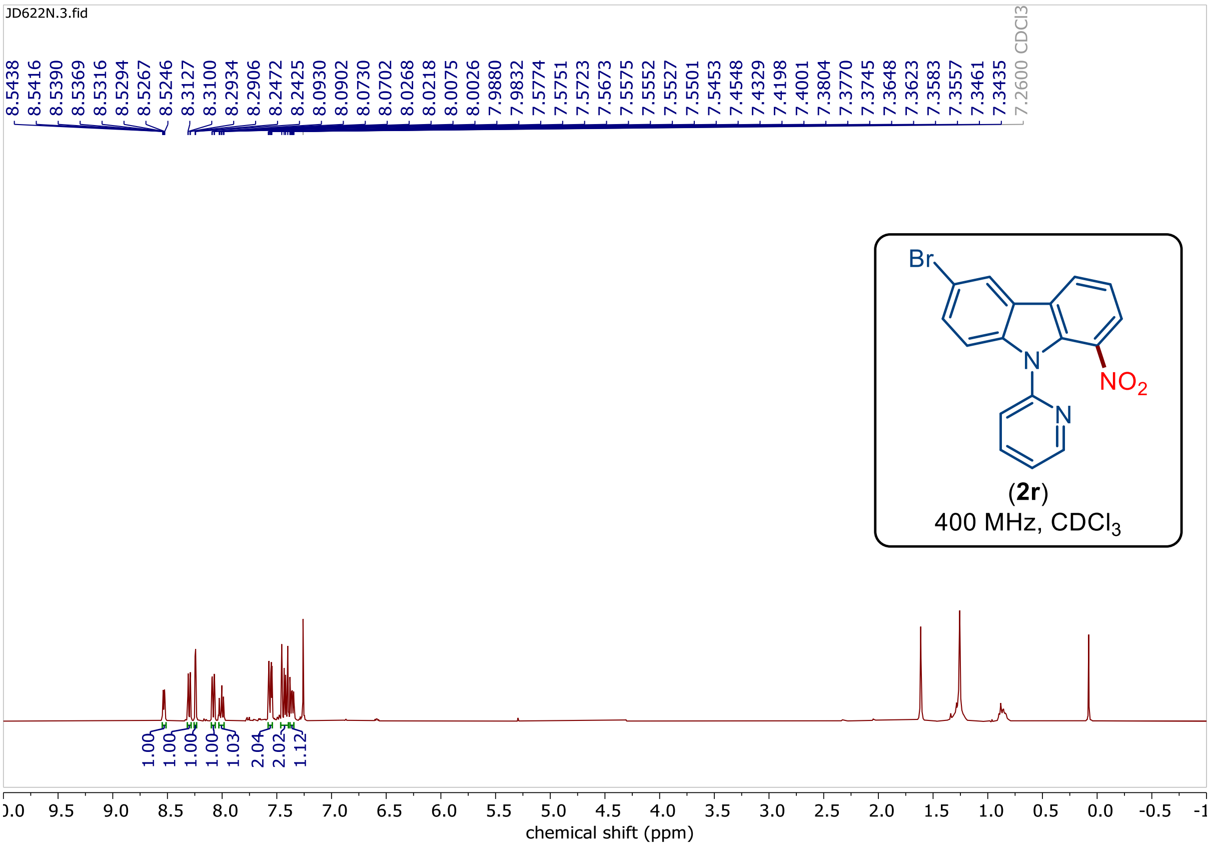

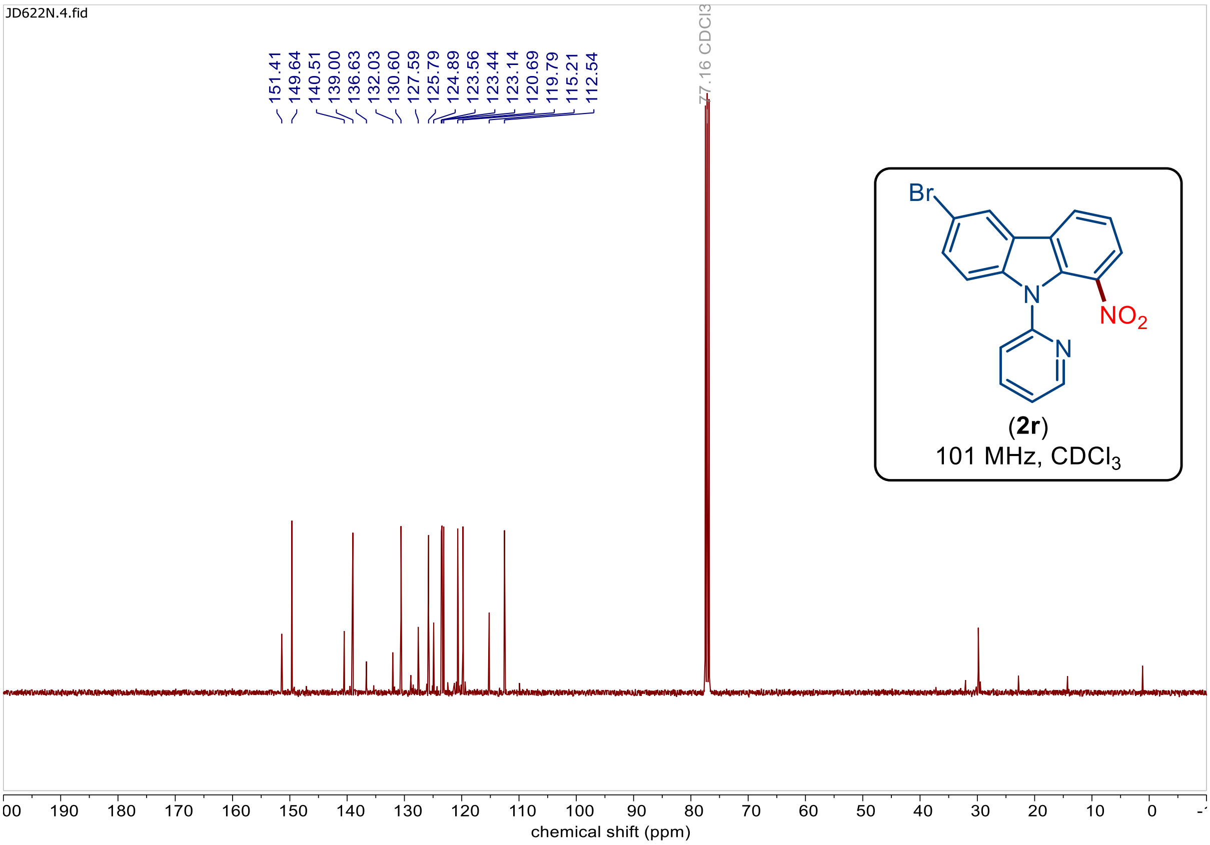

^{1}H and $^{13}C\{^{1}H\}$ NMR spectra of compound **2s**

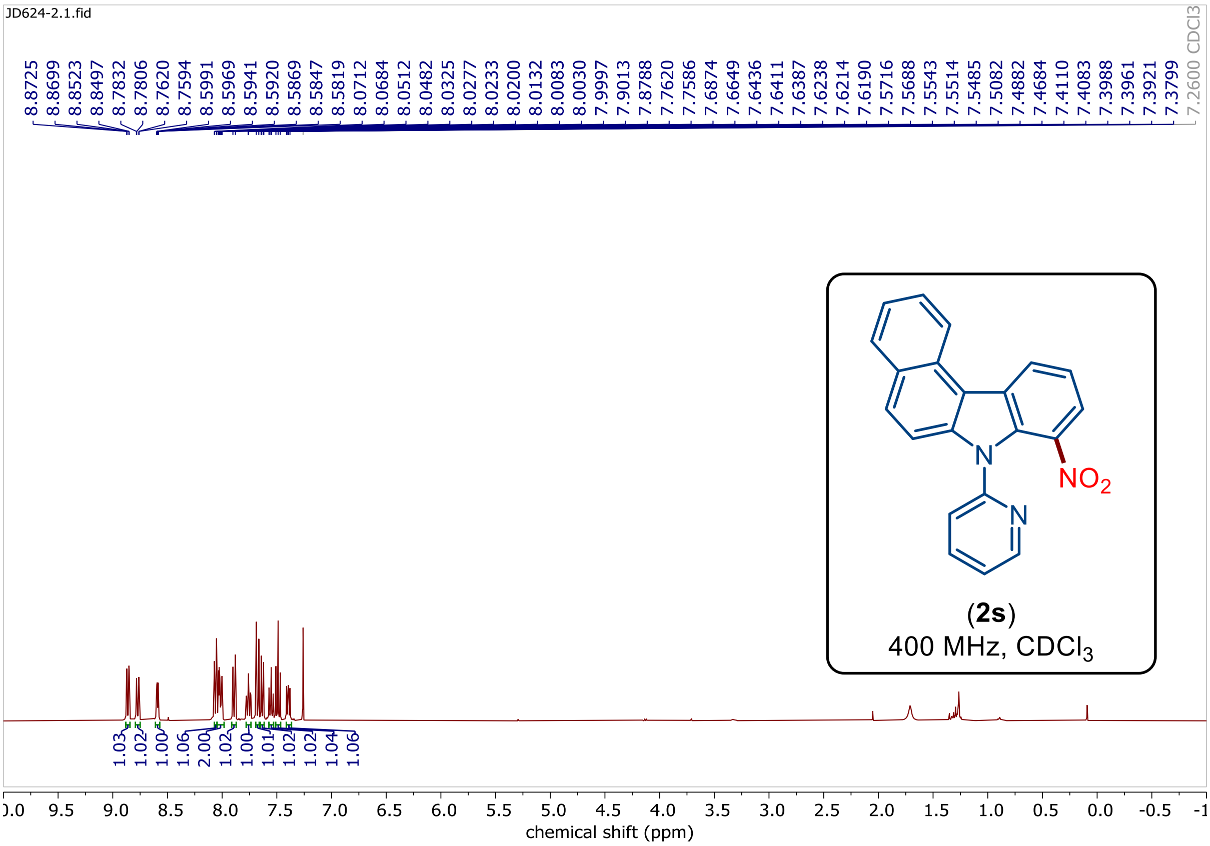

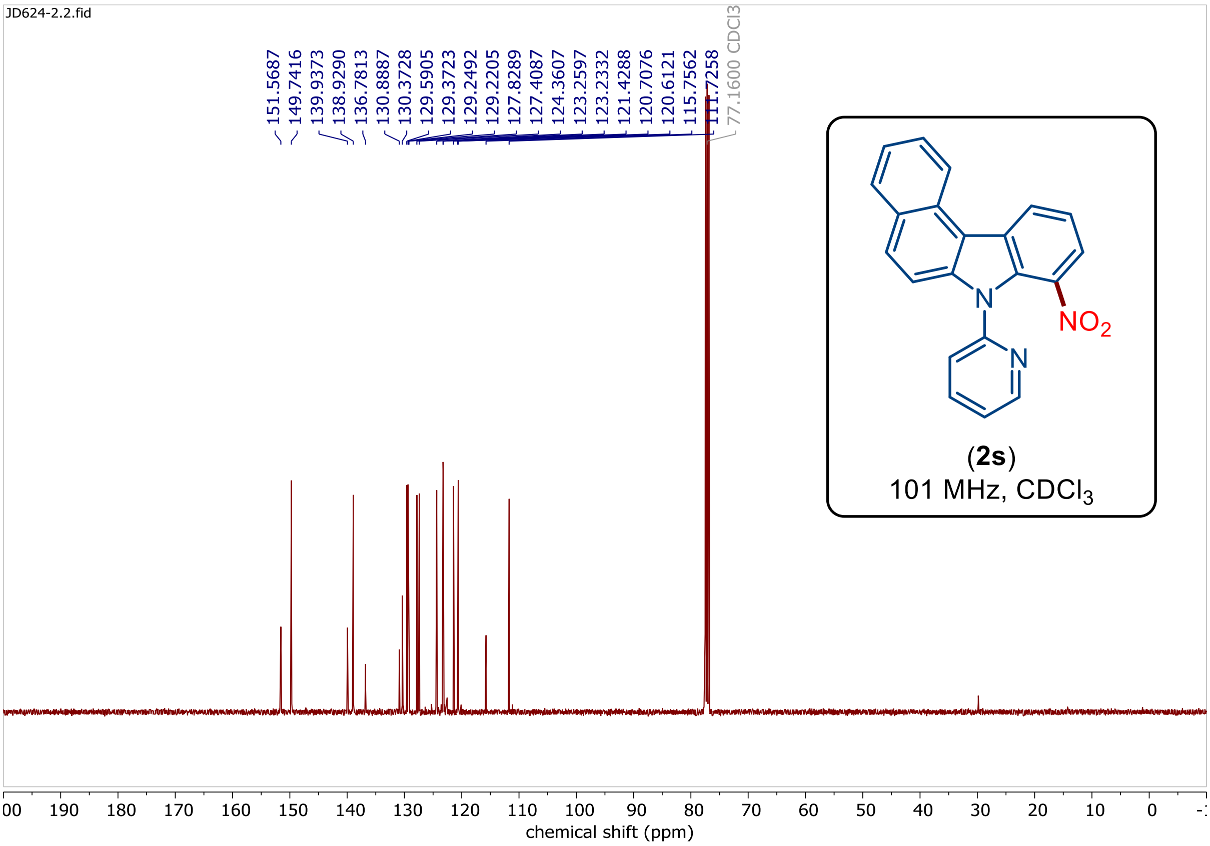

^{1}H and ^{13}C{^{1}H} NMR spectra of compound **2s'**

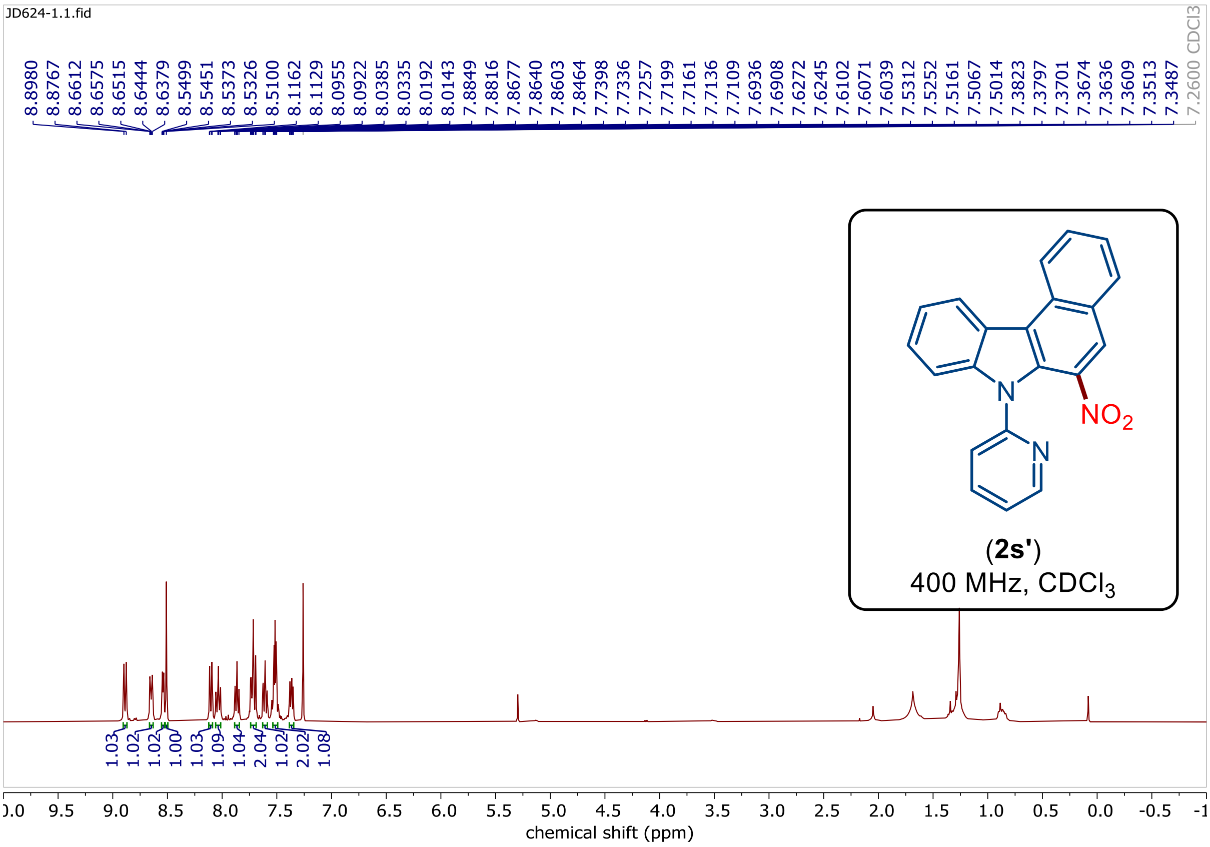

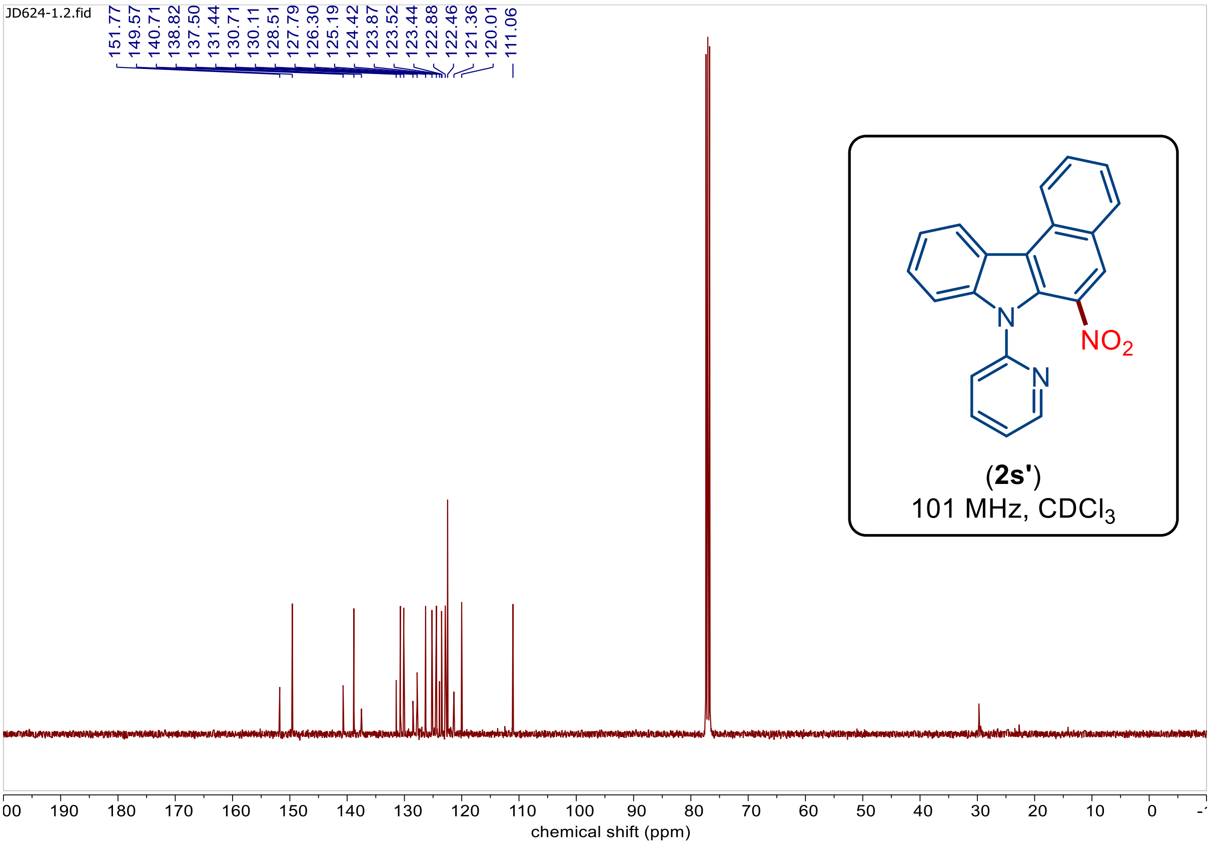

^1H and $^{13}C\{^1H\}$ NMR spectra of compound **3**

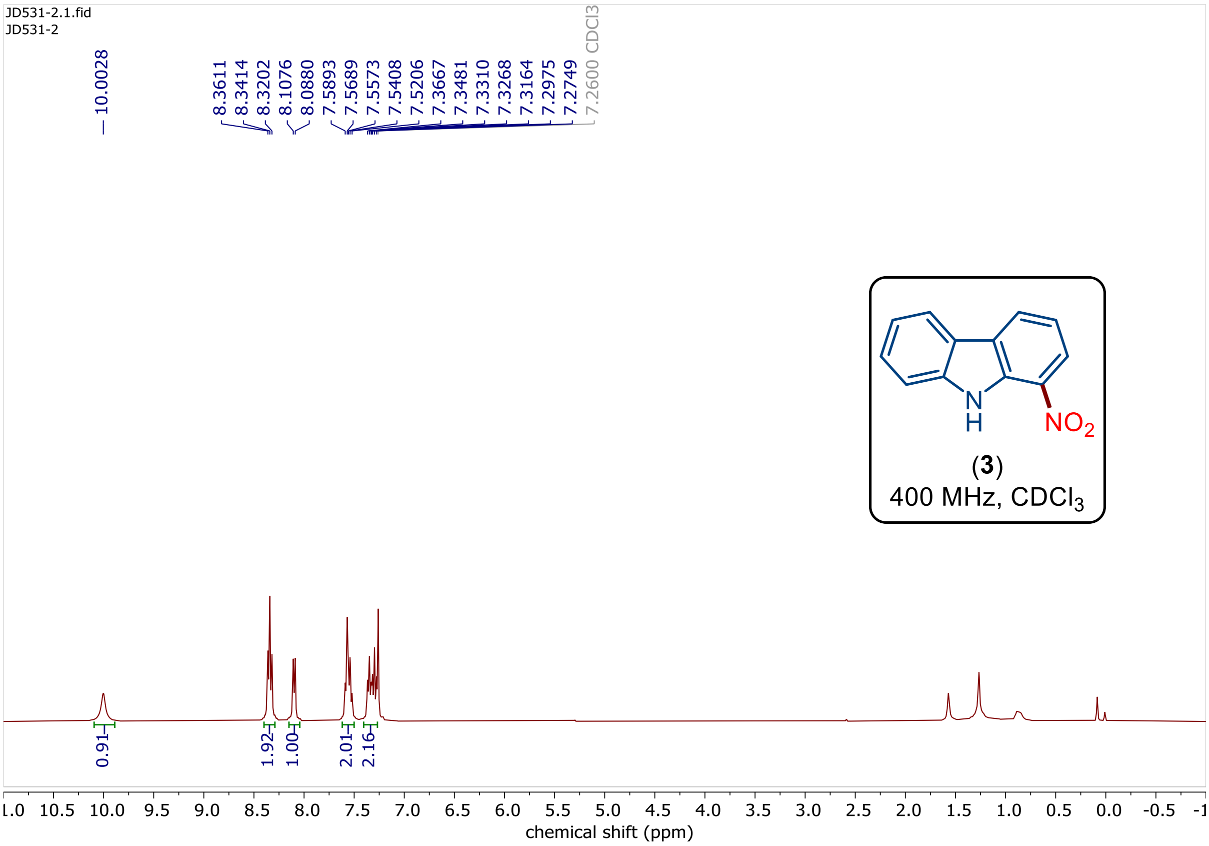

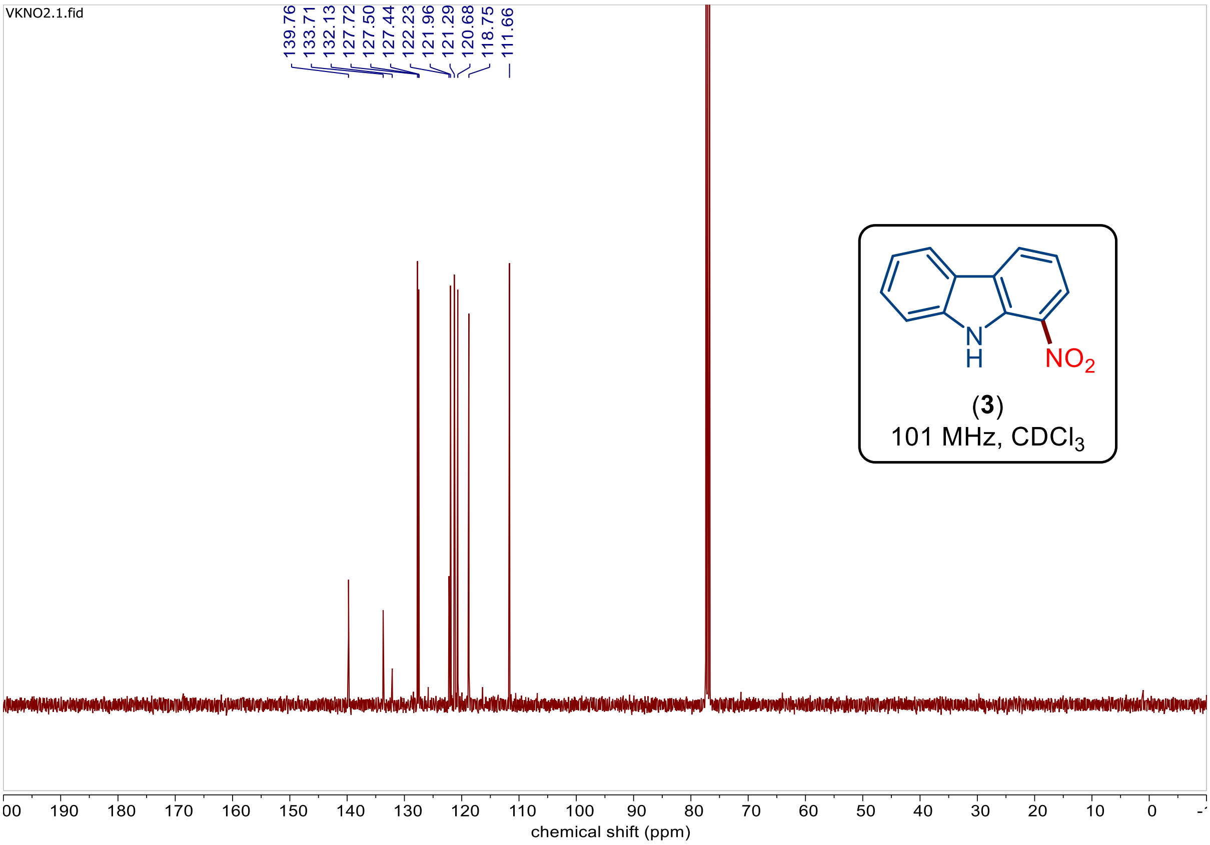

^{1}H and $^{13}C\{^{1}H\}$ NMR spectra of compound **4**

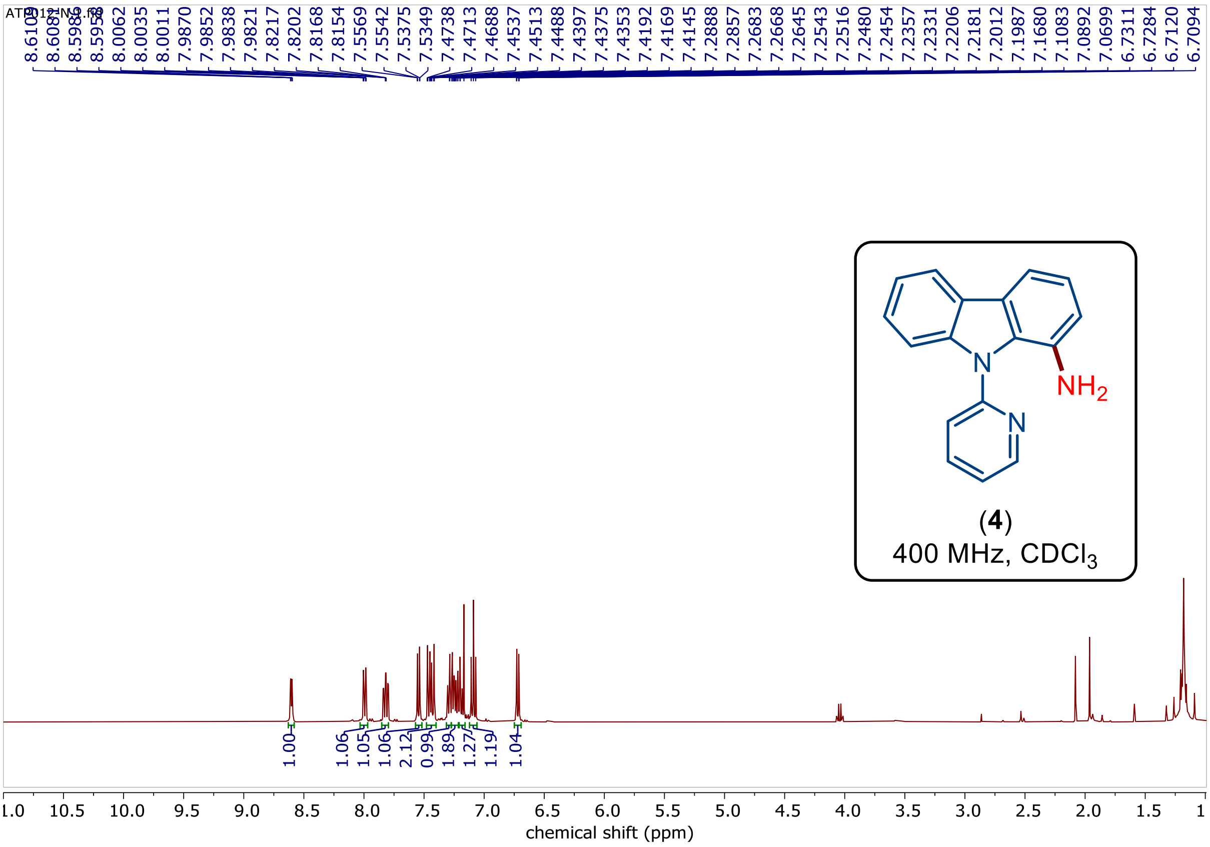

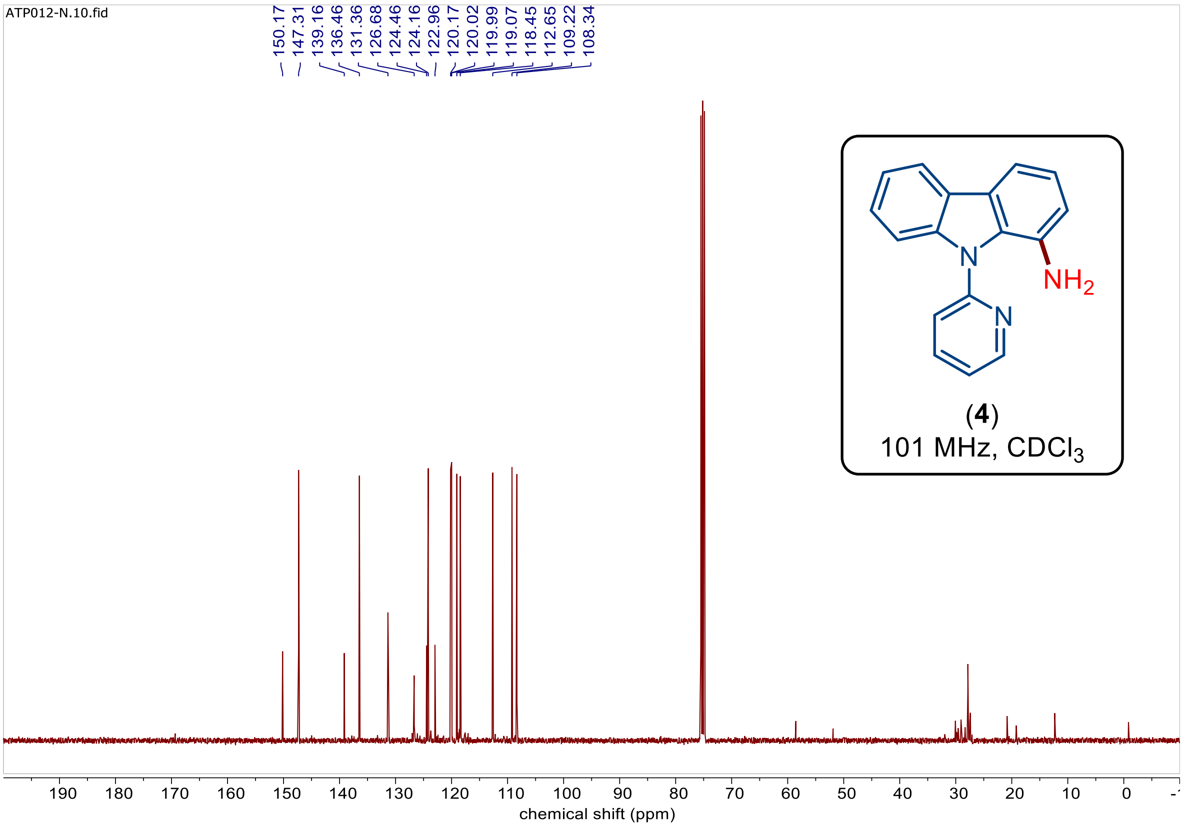

^{1}H and ^{13}C{^{1}H} NMR spectra of compound **5**

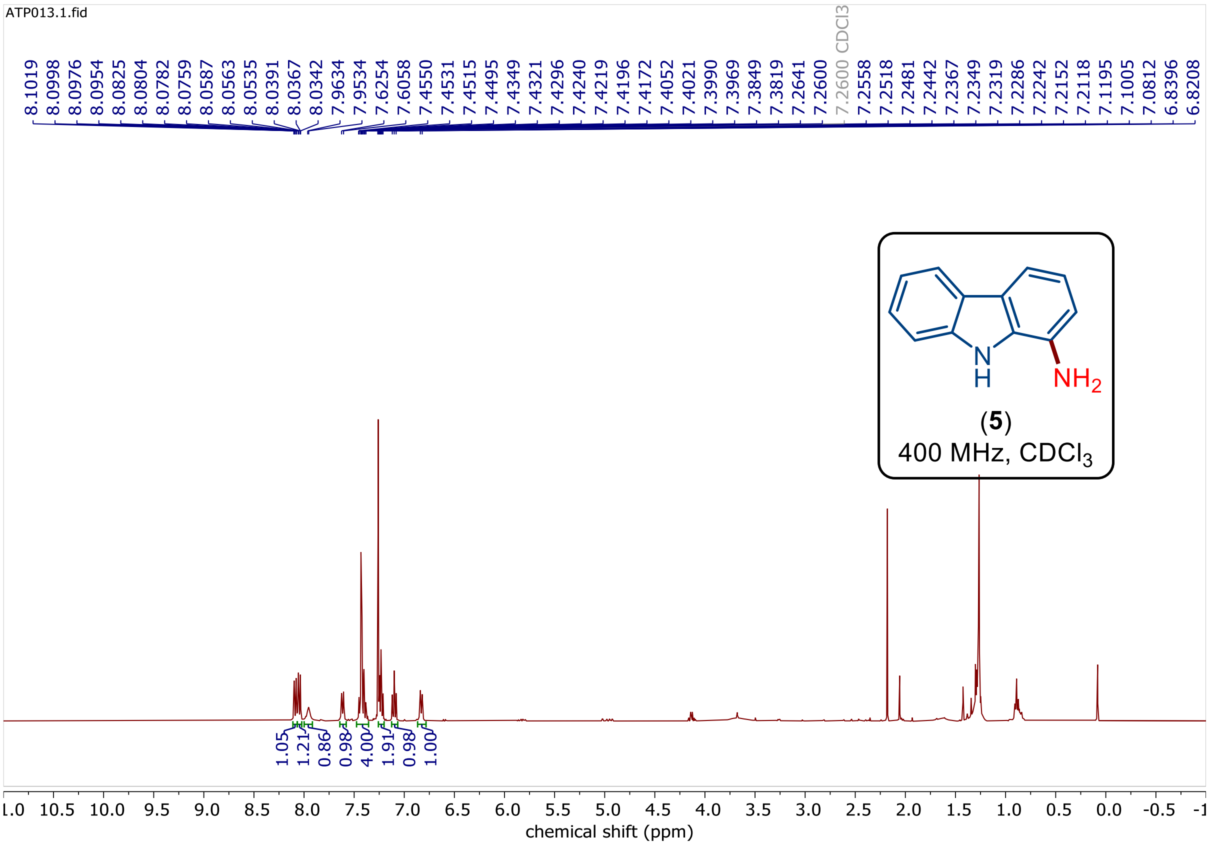

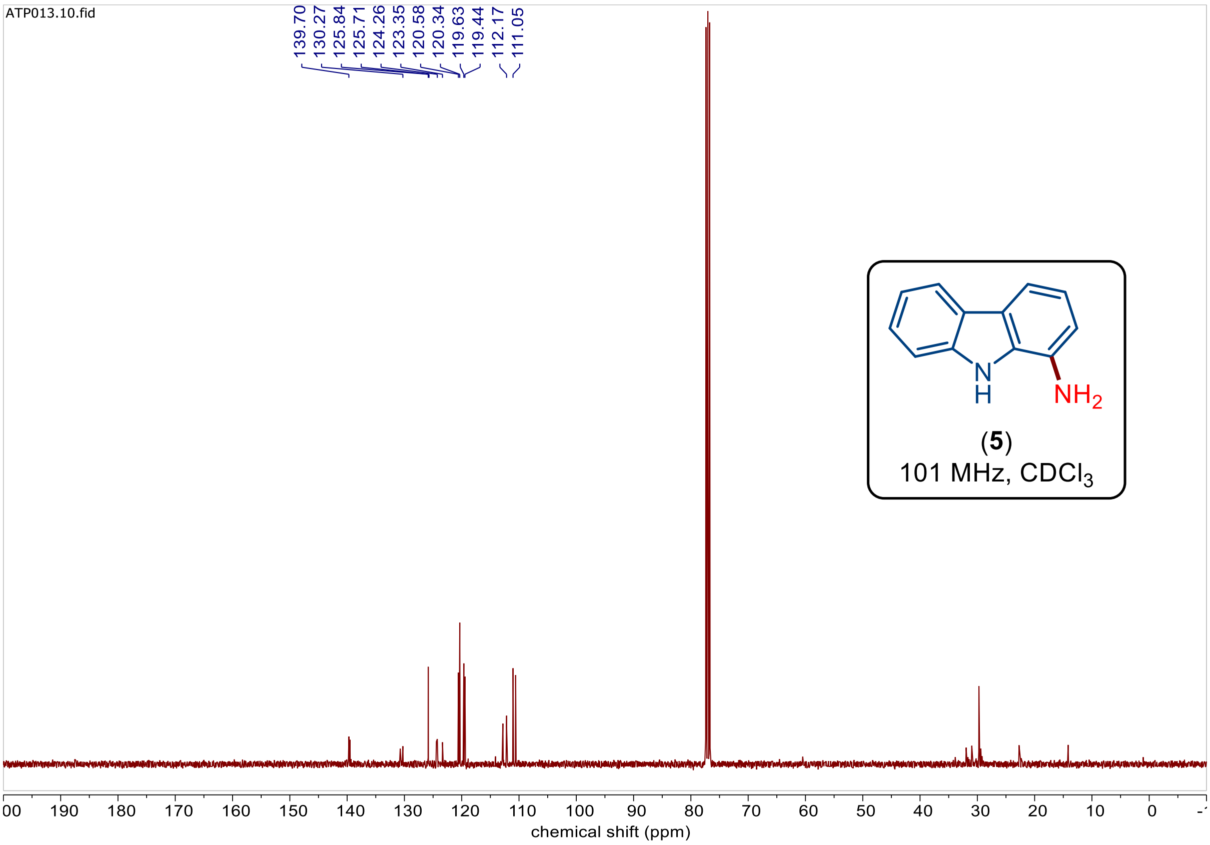

Supplement: File 1 — Experiment details, characterization data, copy of NMR spectra of synthesized compounds, and single-crystal X-ray diffraction data. [file Beilstein_J_Org_Chem-21-2479-s001.pdf]
